# Social capital I: measurement and associations with economic mobility

**DOI:** 10.1038/s41586-022-04996-4

**Published:** 2022-08-01

**Authors:** Raj Chetty, Matthew O. Jackson, Theresa Kuchler, Johannes Stroebel, Nathaniel Hendren, Robert B. Fluegge, Sara Gong, Federico Gonzalez, Armelle Grondin, Matthew Jacob, Drew Johnston, Martin Koenen, Eduardo Laguna-Muggenburg, Florian Mudekereza, Tom Rutter, Nicolaj Thor, Wilbur Townsend, Ruby Zhang, Mike Bailey, Pablo Barberá, Monica Bhole, Nils Wernerfelt

**Affiliations:** 1 Department of Economics, Harvard University, Cambridge, MA USA; 2 Department of Economics, Stanford University, Stanford, CA USA; 3 NYU Stern School of Business, New York, NY USA; 4 Opportunity Insights, Harvard University, Cambridge, MA USA; 5 Grammarly, San Francisco, CA USA; 6 Meta Platforms, Menlo Park, CA USA

**Keywords:** Economics, Sociology

## Abstract

Social capital—the strength of an individual’s social network and community—has been identified as a potential determinant of outcomes ranging from education to health^1–8^. However, efforts to understand what types of social capital matter for these outcomes have been hindered by a lack of social network data. Here, in the first of a pair of papers^9^, we use data on 21 billion friendships from Facebook to study social capital. We measure and analyse three types of social capital by ZIP (postal) code in the United States: (1) connectedness between different types of people, such as those with low versus high socioeconomic status (SES); (2) social cohesion, such as the extent of cliques in friendship networks; and (3) civic engagement, such as rates of volunteering. These measures vary substantially across areas, but are not highly correlated with each other. We demonstrate the importance of distinguishing these forms of social capital by analysing their associations with economic mobility across areas. The share of high-SES friends among individuals with low SES—which we term economic connectedness—is among the strongest predictors of upward income mobility identified to date^10,11^. Other social capital measures are not strongly associated with economic mobility. If children with low-SES parents were to grow up in counties with economic connectedness comparable to that of the average child with high-SES parents, their incomes in adulthood would increase by 20% on average. Differences in economic connectedness can explain well-known relationships between upward income mobility and racial segregation, poverty rates, and inequality^12–14^. To support further research and policy interventions, we publicly release privacy-protected statistics on social capital by ZIP code at https://www.socialcapital.org.

## Abstract (web summary)

Analyses of data on 21 billion friendships from Facebook in the United States reveal associations between social capital and economic mobility.

## Main

Recent work has argued that social capital may play a central role in shaping important social phenomena such as income inequality and economic opportunity^15,16^. However, a lack of large-scale data on social networks has limited the ability of researchers to understand what types of social capital matter for such outcomes and how we can increase effective forms of social capital. For example, the most widely used dataset to study social networks—the National Longitudinal Study of Adolescent to Adult Health (Add Health)—covers approximately 20,000 students at 132 schools in the United States and, owing to small sample sizes, cannot be disaggregated by school. More recent studies have used large-scale mobile phone data to measure ‘experienced segregation’^1,17–21^ but do not directly observe social interactions between different types of people, a distinction that we show is empirically important.

Here, we use data on the social networks of 72.2 million users of Facebook aged between 25 and 44 years to construct and publicly release (https://www.socialcapital.org) new measures of social capital for each ZIP code in the United States. In a companion paper^9^, we also release data on social capital for each high school (secondary school) and college (university). As in previous research using Facebook data^22–26^ (Supplementary Information C.1), we use social network data as a proxy for real-world friendships rather than online interactions per se. As a result, our analysis does not shed light on the effects of online social networks themselves.

We correlate our new measures of social capital with data on economic mobility—children’s chances of rising up the income distribution—across areas and analyse the mechanisms through which social capital and economic mobility are related. We find that the degree to which people with low and high SES are friends with each other (which we term economic connectedness (EC)) is strongly associated with upward income mobility, whereas other forms of social capital are not.

## Measuring social capital

Building on previous work^27–29^, we organize measures of social capital into three categories: (1) cross-type connectedness, which is the extent to which different types of people (for example, high income versus low income) are friends with each other^15,30–32^; (2) network cohesiveness, which is the degree to which friendship networks are clustered into cliques and whether friendships tend to be supported by mutual friends^33^; and (3) civic engagement, which we measure using indices of trust or participation in civic organizations^34,35^.

Cross-type connectedness can be viewed as a form of ‘bridging’ capital, whereas network cohesiveness is more in line with the concept of ‘bonding’ capital^36^. In addition to measuring distinct concepts, these categories of social capital differ in terms of the data they use as inputs. Measures of cross-type connectedness combine data on networks (friendship links) with data on individual characteristics. By contrast, measures of cohesiveness use only data on network links, with no characteristics. Finally, measures of civic engagement do not use data on networks at all and are instead based purely on individual or community-level characteristics (Supplementary Table 6).

We measure these concepts, which are defined more precisely below, using privacy-protected data from Facebook (Methods: ‘Sample construction’ and ‘Privacy and ethics’). We focus on Facebook users with the following attributes: aged between 25 and 44 years who reside in the United States; active on the Facebook platform at least once in the previous 30 days; have at least 100 US-based Facebook friends; and have a non-missing residential ZIP code. We focus on the 25–44-year age range because its Facebook usage rate is greater than 80% (ref. ^37^). On the basis of comparisons to nationally representative surveys and other supplementary analyses, our Facebook analysis sample is reasonably representative of the national population (Methods: ‘Benchmarking’). We use the Facebook data to obtain information on friendships, locations (ZIP code and county), and individuals' SES and their parents' SES. These variables are described in detail in the Methods (‘Variable definitions’).

## Economic connectedness

Many theoretical studies have shown how connections to more educated or affluent individuals can be valuable for transferring information, shaping aspirations and providing mentorship or job referrals^15,30,31,38–44^. Consistent with these models, empirical studies have documented that social ties to well-resourced individuals can materially affect economic and labour market outcomes^3–5,45^. Motivated by this literature, we begin by measuring connectedness across different types of people, focusing on economic connectedness: the extent to which people with low and high SES are friends with each other.

Social scientists have measured SES using many different variables, ranging from income and wealth to educational attainment, occupation, family background, neighbourhood and consumption^46^. To capture these varied definitions, we compute the SES for each individual in our analysis sample by combining several measures of SES, such as average incomes in the individual’s neighbourhood and self-reported educational attainment (see the ‘Privacy and ethics’ section of the Methods for a discussion of how user privacy was protected during this project). We combine these measures of SES into a single SES index using a machine-learning algorithm (Methods (‘Variable definitions’) and Supplementary Information B.1). We then calculate each individual’s percentile rank in the national SES distribution relative to others in their birth cohort. Although we do not observe individuals’ incomes directly, we show that our SES rankings are highly correlated with external, publicly available measures of income across groups (for example, ZIP codes, high schools, and colleges). We also show that using simpler measures of SES, such as median household income in an individual’s ZIP code, produces very similar results to those reported below.

**Fig. 1:**
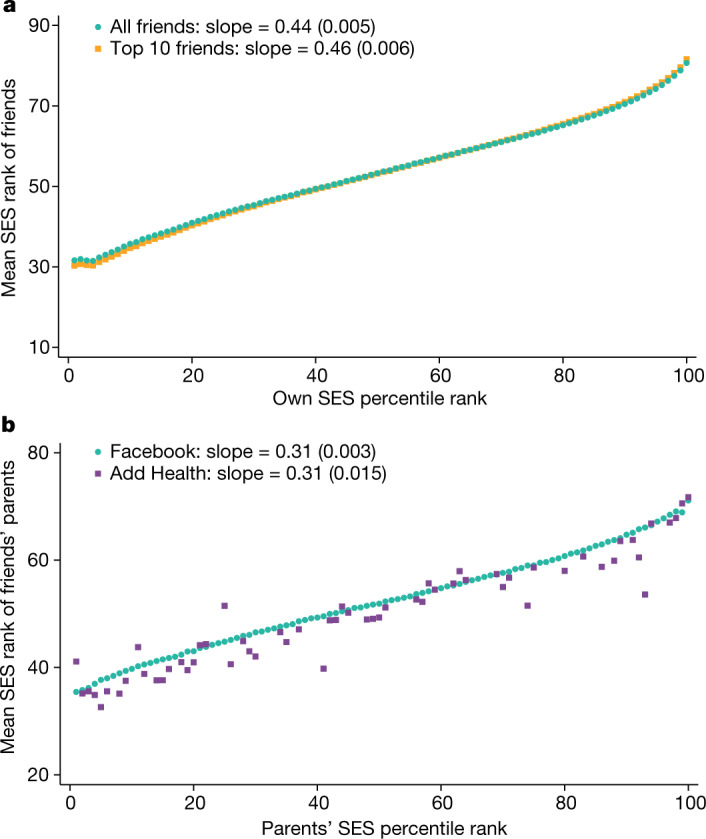
Relationship between an individual’s SES and friends’ SES. **a**, The mean SES rank of individuals’ friends versus their own SES percentile ranks. The series in green circles is calculated using the entire friendship network for each individual. The series in orange squares is constructed using each individual’s ten closest friends based on the frequency of public interactions such as likes, tags, wall posts and comments. SES is constructed by combining information on 22 variables to predict median household incomes in individuals’ residential block groups and then ranking individuals relative to others in the same birth cohort (Methods: ‘Variable definitions’). **b**, Comparison of estimates of homophily in the Facebook data and the Add Health survey. The series in purple squares plots the mean parental income rank of children’s friends against their own parents’ income percentile rank in the Add Health data. The series in green circles presents the analogous relationship in the Facebook data using our SES proxies, restricting the sample to individuals born in 1989–1994 and using their five closest friends from high school to match the Add Health sample as closely as possible (Supplementary Information A.5.2). For each series, we report slopes estimated from a linear regression on the plotted points, with heteroskedasticity-robust standard errors in parentheses.

Figure 1a plots the mean SES rank of individuals’ friends against their own SES ranks. There is strong homophily, whereby individuals with higher SES are friends with higher-SES people. A one percentile point increase in one’s own SES rank is associated with a 0.44 percentile point increase in the SES rank of one’s friends on average. The relationship is almost linear between the 10th and 90th percentiles of the SES distribution, with a slope of 0.41 in that range. The slope rises to 0.98 between the 90th and 100th percentiles, which shows that the highest-SES individuals tend to have particularly high-SES friends. These estimates of homophily are similar (slope of 0.46 for full range, 1.02 between the 90th and 100th percentiles) when we restrict the analysis to an individual’s ten closest friends (defined on basis of the frequency of public interactions such as likes, tags, wall posts and comments). This result shows that our estimates are not significantly affected by the strength of friendships or the number of Facebook friends that people have.

For our analyses below, it is useful to measure connections between individuals in different parts of the SES distribution. For simplicity, in our main analysis, we separate individuals into two groups on the basis of their SES: below-median and above-median SES (which we refer to as low SES and high SES, respectively, below). On average, 38.8% of the friends of below-median-SES individuals have above-median SES, whereas 70.6% of the friends of above-median-SES individuals have above-median SES. As 50% of individuals have above-median SES by definition, high-SES friends are under-represented by 22.4% ($1-\frac{0.388}{0.5}=0.224$) among low-SES individuals relative to their share in the population. By contrast, high-SES friends are over-represented by 41.2% among high-SES individuals ($\frac{0.706}{0.5}-1=0.412$). Note that the share of high-SES friends for low-SES and high-SES individuals averages to 54.7% rather than 50% because high-SES people have more friends than low-SES people on average (Extended Data Table 1).

If high-SES and low-SES individuals were to make friendships independent of SES (that is, there were no homophily by SES) and also were to make the same number of friends on average, then 50% of low-SES individuals’ friends would have high SES. In practice, above-median-SES individuals have 25.4% more friends than below-median-SES individuals on average (Extended Data Table 1). If high-SES people continue to make 25.4% more friends than low-SES people, but friendships were formed independent of SES, the share of high-SES friends among low-SES individuals would be $\frac{1.254}{1+1.254}=55.6 \%$. Relative to that benchmark, low-SES individuals make 30.2% fewer high-SES friends than they would in the absence of homophily.

We go beyond the two-group median split by examining connections between individuals in different deciles of the SES distribution. Extended Data Table 1 presents a matrix of intradecile friendship rates, which shows the likelihood of friendship formation for people from different deciles of the SES distribution. Connectedness is lower between deciles that are further apart. For instance, top-decile friends are under-represented among people in the bottom decile by 75% relative to their population share ($1-\frac{0.025}{0.1}=0.75$). This value is more than three times larger than the corresponding 22.4% under-representation of above-median friends among below-median individuals.

### Childhood economic connectedness

In addition to measuring economic connectedness among adults, we use parent–child linkages to analyse EC based on the childhood friendships of individuals from different family backgrounds. Social capital during individuals’ formative years may be particularly relevant for intergenerational income mobility^30^.

We measure childhood EC by analysing homophily in friendships made in high school by parents’ SES (Methods: ‘Measuring connectedness’). Figure 1b plots the mean parental SES rank of a given individual’s five closest friends in high school against the SES rank of the individual’s own parents. There is less homophily by parental SES during childhood than by own SES in adulthood, with a slope of 0.31 instead of 0.44. Much of this difference in slopes arises from the fact that SES in adulthood among friends from high school is more similar than their parents' SES, perhaps because children who befriend each other tend to follow similar trajectories^9^.

The series represented by squares in Fig. 1b shows analogous estimates of homophily by parental SES rank among high school students using data from Add Health, a representative survey of students that contains self-reported information on close friendships (Supplementary Information A.5.2). We obtain highly similar point estimates of homophily (slope = 0.31) by parental SES rank among high school friends in the Facebook and Add Health data. This comparison suggests that selection biases in Facebook usage or measurement error in friendship links and SES ranks do not substantially distort our estimates of homophily.

### Economic connectedness across areas

The Facebook dataset, which is about 3,500 times larger than the Add Health sample, offers adequate precision and information to allow us to measure EC not just at the national level but also within specific communities, such as a given neighbourhood or school. We define the level of economic connectedness in a community as the average share of above-median-SES friends among below-median-SES members of that community divided by 50% to quantify the average degree of under-representation of high-SES friends among low-SES people (an algebraic definition is provided in the Methods: ‘Measuring connectedness’). A value of 0 for EC implies that a network has no connections between low-SES and high-SES people, whereas a value of 1 implies that low-SES people have an equal number of low-SES and high-SES friends. Although we focus on economic connectedness among low-SES individuals in particular, which we refer to simply as EC, we also construct and release analogous measures of community-level economic connectedness for high-SES individuals.

**Fig. 2:**
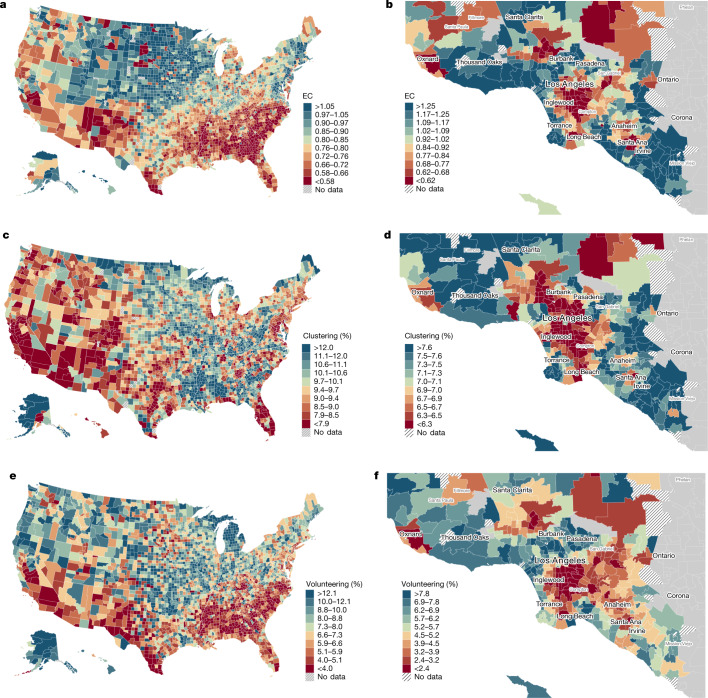
The geography of social capital in the United States. **a**, County-level map of EC, defined as twice the share of friends with above-median SES among people with below-median SES. **b**, ZIP-code-level map of EC in Los Angeles. **c**, County-level map of average clustering, defined as the share of an individual’s friend pairs who are friends with each other. **d**, ZIP-code-level map of average clustering in Los Angeles. **e**, County-level map of volunteering rates, defined as the percentage of individuals who are members of volunteering or activism groups as classified by Facebook. **f**, ZIP-code-level map of volunteering rates in Los Angeles. We omit counties and ZIP codes where statistics are estimated on fewer than 100 Facebook users with below-median SES. These maps must be viewed in colour to be interpretable. Analogous maps for all ZIP codes in the United States are available at https://www.socialcapital.org. Extended Data Fig. 1 presents county-level maps of other social capital measures. Maps were made with the QGIS software package.

Figure 2a maps EC by county in the United States. EC varies significantly across areas. Counties in the bottom decile of connectedness have EC values less than 0.58. That is, below-median-SES individuals have about 42% fewer above-median-SES friends than one would expect in the absence of homophily. Counties in the top decile have EC values of 1.05 or higher, approximately commensurate to what one would expect on the basis of random sampling of friends from the national distribution, adjusting for the fact that high-SES people make more friends, as discussed above. This geographical variation in connectedness is partially driven by differences in the share of high-SES individuals in an area and partly by differences in the rates at which low-SES individuals befriend high-SES individuals in their area. We decompose the relative contributions of these two factors, which we refer to as exposure and friending bias, in the companion paper^9^.

EC is generally lowest in the Southeast, the Southwest and industrial cities in the Midwest. It is highest in the rural Midwest and on the East Coast. The mean standard error of the county-level EC estimates is 0.004 (Methods (‘Measuring connectedness’) and Supplementary Information B.3), which implies that nearly all of the variation in Fig. 2a reflects true differences in EC across areas rather than sampling error.

EC varies not just across counties but also across neighbourhoods within counties: 42% of the variation in EC across ZIP codes is within counties. Figure 2b illustrates this local variation by mapping EC by ZIP code (formally, ZIP code tabulation areas) in the Los Angeles metropolitan area (analogous maps for all ZIP codes in the United States are available at https://www.socialcapital.org). EC ranges from 0.62 to 1.25 between ZIP codes at the 10th and 90th percentiles of the EC distribution within the Los Angeles metro area (Los Angeles, Orange and Ventura counties). EC is lowest in the lowest-income neighbourhoods of Los Angeles, such as Watts in central Los Angeles, where EC is 0.45. EC is generally higher in higher-income areas, but there is significant variation in EC even within those areas, with some places (such as Echo Park) having relatively low EC despite having many high-SES residents.

More broadly, looking outside Los Angeles, almost none of the lowest-income ZIP codes in the United States exhibit high levels of EC. It may be that there is little scope for people with low SES to connect with individuals with higher SES if there are few such people in the vicinity, echoing Blau's observation that “persons cannot associate without having opportunities for contact”^47^. In our analysis, this point is an empirical result rather than a mechanical consequence of contact because low-SES individuals in low-income areas could in principle befriend high-SES people outside their neighbourhoods. In practice, such connections appear to be relatively rare. However, the presence of high-SES neighbours does not guarantee that low-SES people connect with those individuals, as many higher-income neighbourhoods still have EC substantially below 1.

The spatial patterns documented above are robust to the way in which economic connectedness is measured. For example, Supplementary Table 1 shows that similar spatial patterns for EC are obtained when restricting attention to individuals' ten closest friends (correlation = 0.99 across counties). Similarly, the mean friend rank of individuals at the 25th percentile of the SES distribution, a measure that controls for differences in the SES distributions within the below-median and above-median groups, has an across-county correlation of 0.98 with our baseline EC measure. The share of top-quintile-SES friends among bottom-quintile-SES individuals in a county has a correlation of 0.74 with our baseline below- versus above-median EC measure across counties. Childhood EC also exhibits broadly similar spatial patterns. We analyse two measures of childhood EC: one constructed for Facebook users from the SES of parents of high school friends and the other constructed for a sample of current 13–17 year olds who use Instagram (Methods: ‘Measuring connectedness’).  We obtain across-county correlations of 0.61 for the Facebook childhood EC measure and 0.82 for the Instagram measure with our baseline EC measure (Supplementary Table 1). The high correlation with EC measured using Instagram for recent birth cohorts suggests that differences in economic connectedness across areas are relatively stable over time, which is consistent with the high degree of serial correlation in our baseline county-level EC measure across birth cohorts (Supplementary Fig. 1).

### Connectedness by other attributes

**Extended Data Fig. 1:**
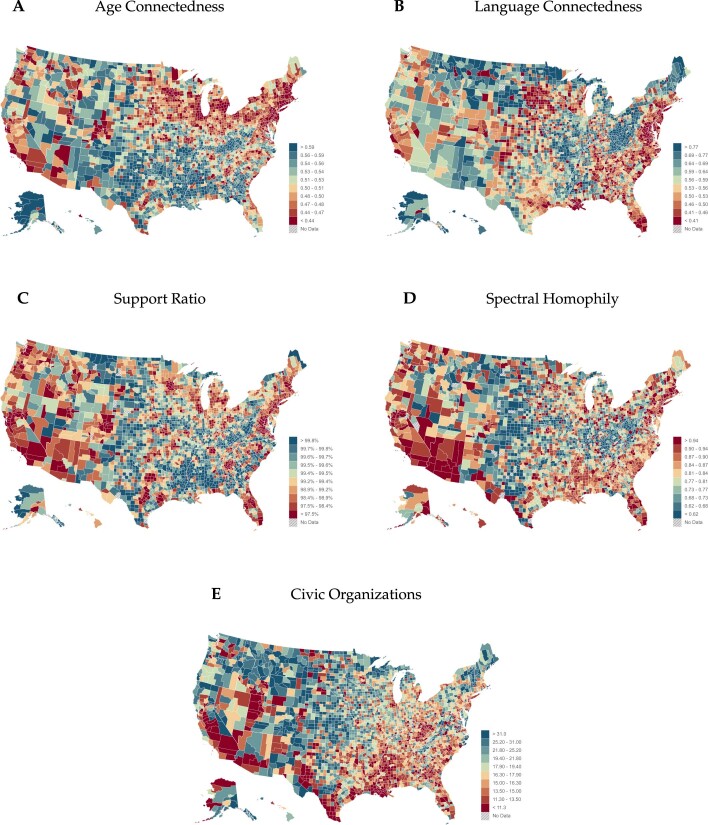
Geographical Variation in Other Social Capital Measures. This figure presents county-level maps analogous to those in Fig. 2 for other measures of social capital. These maps must be viewed in color to be interpretable. Age connectedness (Panel A) is the average share of friends who are 35 to 44 among users who are 25 to 34, normalized by the share of individuals who are 35 to 44. Language connectedness (Panel B) is the average share of friends who set their Facebook language to English among individuals who do not set their Facebook language to English, normalized by the share of people who set their Facebook language to English. Support ratio (Panel C) is the share of friendships between people in the county who have at least one other friend in the county in common. Spectral homophily (Panel D) is the second largest eigenvalue of the row-stochasticized network adjacency matrix, a measure of the extent to which the county-level friendship network is fragmented into separate groups. Civic organizations (Panel E) is the number of civic organizations with Facebook pages per 1,000 Facebook users in the county. See the Economic connectedness, Cohesiveness, and Civic engagement sections of Main Text and Methods for details on the definitions and construction of these measures.

We also measure connectedness between individuals who use English as their primary language versus those who do not, and individuals between the ages of 25 and 34 years versus individuals between the ages of 35 and 44 years. Language and age connectedness exhibit different spatial patterns from EC (Extended Data Fig. 1). For example, the across-county correlation between language connectedness and EC is only 0.10 (Table 1). Hence, it is not simply that some areas exhibit high levels of connectedness across all types of individuals; rather, the degree of connectedness varies across different characteristics.

**Table 1:** Correlation matrix for social capital measures across counties

	(1)	(2)	(3)	(4)	(5)	(6)	(7)	(8)	(9)
(1) Economic connectedness	1.00	–	–	–	–	–	–	–	–
(2) Language connectedness	0.10	1.00	–	–	–	–	–	–	–
(3) Age connectedness	–0.45	0.17	1.00	–	–	–	–	–	–
(4) Clustering	0.01	0.38	0.51	1.00	–	–	–	–	–
(5) Support ratio	-0.25	0.30	0.50	0.64	1.00	–	–	–	–
(6) Spectral homophily	–0.09	–0.37	–0.49	-0.61	–0.51	1.00	–	–	–
(7) Penn State index	0.31	0.08	–0.04	0.39	0.28	–0.25	1.00	–	–
(8) Civic organizations	0.27	0.16	0.05	0.37	0.23	–0.33	0.67	1.00	–
(9) Volunteering rate	0.46	0.28	–0.04	0.30	0.23	–0.35	0.44	0.46	1.00

This table reports county-level pairwise correlations of the primary social capital measures that we analyse, weighted by the number of children with below-median parental income in each county as calculated in the Opportunity Atlas^72^ using Census data. Economic connectedness is twice the share of above-median-SES friends among below-median-SES people. Language connectedness is the share of friends who set their Facebook language to English among users who do not set their language to English, divided by the national share of users who set their language to English. Age connectedness is the share of friends who are aged 35–44 years among users who are aged 25–34 years, divided by the national share of users aged 35–44 years. Clustering is the share of an individual’s friend pairs who are also friends with each other, averaged over all individuals in the county. Support ratio is the share of friendships between people in the county with at least one other mutual friend in the county. Spectral homophily is the second largest eigenvalue of the row-stochasticized network adjacency matrix, a measure of the extent to which the county-level friendship network is fragmented into separate groups. The Penn State index^63^ is an index of participation in civic organizations and other measures of civic engagement. Civic organizations is the number of civic organizations with Facebook pages per 1,000 Facebook users in the county. Volunteering rate is the percentage of Facebook users in the county who are members of volunteering or activism groups. See Supplementary Table 1 for an expanded version of this correlation table that includes all social capital measures that we construct. Further details on all the social capital measures are provided in the Methods (‘Variable definitions’).

## Cohesiveness

Many theoretical studies have shown how the structure of social networks can shape a variety of outcomes, from the formation of human capital to the degree of adherence to social norms^33,48,49^. These studies of social capital conceptualize the cohesiveness of networks in two ways: (1) the cohesiveness of a given individual’s personal network (measured, for example, by the extent to which their friends are in turn friends with each other), and (2) the cohesiveness of the whole community (measured by the degree of fragmentation into subcommunities). Empirical studies have shown that these measures are associated with a range of outcomes, including the dynamics of various types of contagion^50–57^. Motivated by this literature, we construct three measures of social capital that characterize the structure of friendship links in a community.

The first measure is clustering, which is the rate at which two friends of a given person are in turn friends with each other. The logic underlying clustering as a measure of social capital is that if a person’s friends are friends with each other, they can act together to pressure or sanction that person, which enforces norms and induces pro-social behaviour and investment. Clustering ranges from 0 to 1, with a value of 0 meaning that all of a person’s friends are isolated from each other and 1 meaning that all of a person’s friends are friends with each other. We measure the degree of clustering in a community as the average rate of clustering in friendships for people living in that community (Methods: ‘Measuring cohesiveness’).

A related, but distinct, measure of cohesiveness is the support ratio, which captures the rate at which pairs of friends in a community have other friends in common. The potential role of this measure of social capital can be microfounded in game theoretic models of the extent to which cooperative behaviour between two individuals can be sustained. Specifically, when two people have friends in common, their mutual friends can witness their behaviour and react to it by enforcing norms^58^. We say that a friendship between two people is supported if they have at least one other friend in common. We measure the support ratio in a given community as the share of friendships among its members that are supported (Methods: ‘Measuring cohesiveness’). The support ratio of a community varies from 0 to 1, with 0 implying that none of the friendships between members of a community are supported, and 1 implying that all such friendships are supported.

The third measure of network cohesiveness we consider is spectral homophily, which captures the extent to which a network is fragmented into separate groups (a formal definition is provided in the Methods: ‘Measuring cohesiveness’)^59^. Spectral homophily also ranges from 0 to 1. A value of 0 implies that there is no homophily, such that individuals are equally likely to be friends with any other member of the community. By contrast, a value of 1 implies that the network fragments into two or more distinct groups across which no one interacts.

All three of these measures of network cohesiveness exhibit broadly similar spatial patterns, with absolute pairwise correlations of 0.51–0.64 with each other across counties (Table 1). In general, clustering and support ratios are highest in the South, Appalachia and rural Midwest (Fig. 2c and Extended Data Fig. 1c). Spectral homophily tends to be lowest in these areas and highest in the Southwest (Extended Data Fig. 1d). Dense urban centres often exhibit high levels of spectral homophily and low levels of clustering, consistent with Coleman's prediction^33^ that areas with greater levels of geographical mobility will have less clustered networks.

The network cohesiveness measures exhibit different geographical patterns from economic connectedness, with correlations ranging from –0.25 to 0.01 with EC across counties (Table 1 and Fig. 2a,c). These differences emerge not just across counties but across neighbourhoods within counties, as illustrated by the ZIP-code-level maps of the Los Angeles metro area (Fig. 2b,d).

## Civic engagement

A third widely discussed concept of social capital is based on levels of civic engagement and pro-social behaviour rather than on the structure of networks^35,60,61^. This form of social capital has been measured using self-reported levels of trust, rates of volunteering or rates of membership in local organizations^62–64^. Such measures are often associated with various outcomes across regions and countries, ranging from economic growth to political accountability^36,65–68^.

Because they do not rely on network data, state-level and county-level indices of civic engagement based on survey data are widely available. Here, we build on previous efforts by constructing measures of civic engagement at the more granular ZIP-code level, taking advantage of the large sample sizes available in the Facebook data.

A common way to measure civic engagement is on the basis of rates of volunteering^64^. Building on previous work^69^, we construct a proxy for the rate of volunteering in an area based on the share of Facebook users in that area who are members of at least one volunteering or activism group as classified based on their titles. Such groups include, for example, Neighbors Helping Neighbors or Adopt a Senior (Methods: ‘Measuring civic engagement’). This measure has a population-weighted correlation of 0.58 with survey-based measures of volunteering rates across states from the Social Capital Project^64^, which suggests that it captures a similar concept.

Another prominent measure of civic engagement is the density of civic organizations in a county^63^. We construct a granular measure of the density of civic organizations (for example, non-profits) based on the number of Facebook pages for such organizations in an area divided by its population (Methods: ‘Measuring civic engagement’). Our index has a population-weighted correlation of 0.67 with the Penn State index^63^ across counties (Table 1).

Our two measures of civic engagement vary substantially across areas and exhibit similar geographical patterns, with a population-weighted correlation of 0.46 across counties. Rates of volunteering are highest in the Pacific Northwest and lowest in the Southeast (Fig. 2e). Civic organizations are most common in the Rocky Mountains, Pacific Northwest and New England, and least common in parts of the South (Extended Data Fig. 1e). Both measures of civic engagement also vary substantially across ZIP codes within counties (Fig. 2f).

Civic engagement is positively correlated with both measures of network cohesiveness and measures of economic connectedness (Table 1). Most notably, volunteering rates have a correlation of 0.46 with EC across counties.

In summary, the new measures of social capital constructed here underscore the importance of specifying a particular notion of social capital when assessing the level of social capital in a community. This result is in line with previous observations based on ethnographic and theoretical analyses^27–29^ that have illustrated how a single community can exhibit different levels of social capital depending on the concept being measured. For example, one study^27^ noted that “since the publication of Stack^70^, sociologists know that everyday survival in less wealthy urban communities frequently depends on close interaction with kin and friends in similar situations. The problem is that such ties seldom reach beyond the inner city, thus depriving their inhabitants of sources of information about employment opportunities elsewhere and ways to attain them”. Our quantitative measures confirm these ethnographic observations in specific communities on a national scale, showing, for example, that high-poverty urban communities with highly cohesive networks often do not provide connections to individuals with high SES.

The benefit of having measures of social capital for all communities in the United States is that they can be used to study which types of social capital matter for various outcomes of interest. In the next section, we investigate which forms of social capital are associated with one prominent outcome that many have hypothesized to rely on social capital: upward economic mobility.

## Social capital and upward income mobility

Rates of upward income mobility—children’s chances of rising up the income distribution conditional on growing up in low-income families—vary substantially across areas in the United States^10^. A large body of literature has sought to understand and explain these differences. A widely discussed hypothesis, based on indirect proxies and ethnographic evidence, is that differences in economic mobility across areas may be related to differences in social capital^15,16,71^.

In this section, we study this hypothesis by analysing the associations between the measures of social capital constructed above and economic mobility across areas. We obtain statistics on intergenerational income mobility and other related outcomes, such as high school graduation rates and teenage birth rates, from the publicly available Opportunity Atlas^72^, which constructs these statistics on the basis of Census and tax data covering all children born in the United States between 1978 and 1983. We focus on correlations between upward mobility and social capital across areas rather than individuals because area-level variation is arguably more likely to be driven by institutional, policy-relevant factors than individual-level variation. Furthermore, we have precise measures of economic mobility (constructed using tax data) at the area level. At the individual level, estimates of income mobility using Facebook data have greater measurement error, which could inflate correlations between one’s own outcomes and friends’ SES.

We begin by examining correlations between social capital and economic mobility across counties and then turn to a more granular ZIP-code-level analysis.

### County-level correlations

**Fig. 3:**
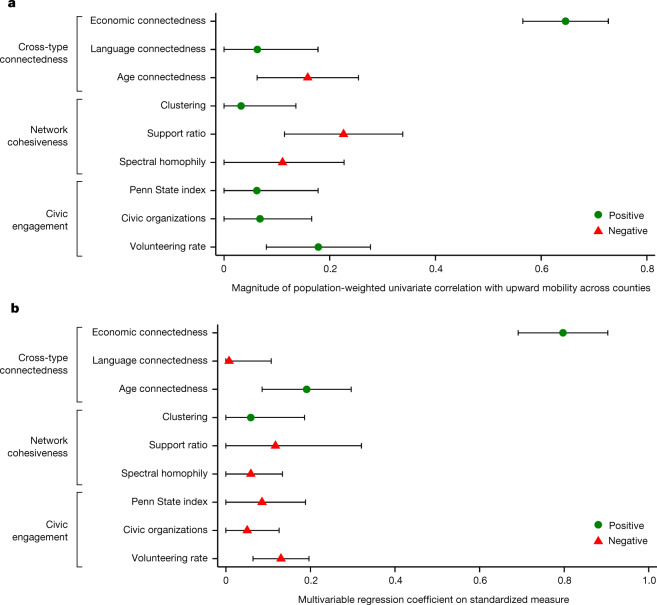
County-level correlations between upward income mobility and measures of social capital. **a**, County-level univariate correlations of upward income mobility with social capital measures. Extended Data Table 2 lists the correlation coefficients plotted here. **b**, Estimates from a multivariable regression of upward income mobility on all variables in **a** together, standardizing the outcome and dependent variables to have a mean of zero and a standard deviation of one. Upward income mobility is obtained from the Opportunity Atlas^72^ and is measured as the predicted household income rank in adulthood for children in the 1978–1983 birth cohorts with parents at the 25th percentile of the national income distribution. Economic connectedness (EC) is twice the share of above-median-SES friends among below-median-SES people. Language connectedness is the share of friends who set their Facebook language to English among users who do not set their language to English, divided by the national share of users who set their language to English. Age connectedness is the share of friends who are aged 35–44 years among users who are aged 25– 34 years, divided by the national share of users aged 35-44 years. Clustering is the share of an individual’s friend pairs who are also friends with each other, averaged over all individuals in the county. Support ratio is the share of friendships between people in the county with at least one other mutual friend in the county. Spectral homophily is the second largest eigenvalue of the row-stochasticized network adjacency matrix, a measure of the extent to which the county-level friendship network is fragmented into separate groups. The Penn State index^63^ is an index of participation in civic organizations and other measures of civic engagement. Civic organizations is the number of civic organizations with Facebook pages per 1,000 Facebook users in the county. Volunteering rate is the percentage of Facebook users in the county who are members of volunteering or activism groups. All correlations and regressions are weighted by the number of children in each county whose parents have below-national-median income. Intervals represent 95% confidence intervals calculated using standard errors clustered by commuting zone.

Figure 3a reports univariate correlations (weighted by the number of children with below-national-median parental income) across counties between each measure of social capital constructed above and upward income mobility (Extended Data Table 2). We define upward income mobility in each county as the average income percentile rank in adulthood of children who grew up in that county with parents at the 25th percentile of the national parental household income distribution^72^. EC is strongly positively correlated with upward income mobility (correlation = 0.65, s.e. = 0.04), whereas all the other measures of social capital are not strongly related to mobility.

**Fig. 4:**
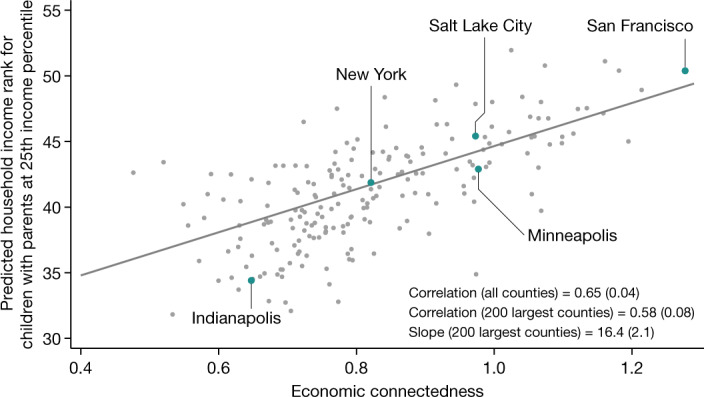
Association between upward income mobility and EC across counties. Scatter plot of upward income mobility against economic connectedness (EC) for the 200 most populous US counties. EC is defined as twice the share of above-median-SES friends among below-median-SES individuals living in the county. Upward income mobility is obtained from the Opportunity Atlas^72^ and is measured as the predicted household income rank in adulthood for children in the 1978–1983 birth cohorts with parents at the 25th percentile of the national income distribution. We report a slope estimated using an ordinary least squares (OLS) regression on the 200 largest US counties by population, with standard errors clustered by commuting zone in parentheses. We also report the population-weighted correlation between upward mobility and EC across both the 200 largest counties as well as all counties, with standard errors (clustered by commuting zone) in parentheses. The correlations and regression are weighted by the number of children in each county whose parents have below-national-median income.

Figure 4 shows the relationship between EC and mobility non-parametrically by presenting a scatter plot of upward income mobility versus EC for the 200 most populous counties. Children who grow up in counties where low-SES individuals have more high-SES friends tend to have much higher rates of upward mobility. As an example, low-SES individuals have a much larger share of high-SES friends in Minneapolis (49%, corresponding to an EC of 0.98) compared with Indianapolis (32%, EC of 0.65). Correspondingly, children who grow up in low-income families have much higher incomes in adulthood in Minneapolis than in Indianapolis. In Minneapolis, children reach the 43rd percentile of the household income distribution on average at age 35 years (roughly US$34,300 in 2015), compared with the 34th percentile ($24,700) in Indianapolis.

**Extended Data Fig. 2:**
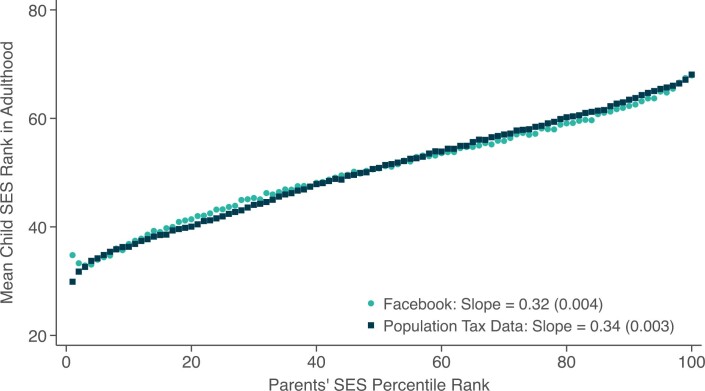
Intergenerational Persistence of Socioeconomic Status in Facebook and Tax Data. This figure shows binned scatter plots of children’s mean SES ranks in adulthood against their own parents’ SES ranks. Each point plots the mean SES rank of children who have parents at a given percentile of the SES distribution. The series in circles is based on data from Facebook, with SES rank calculated as described in the Variable Definitions section of Methods. The series in squares is based on administrative tax data analysed in prior work^90^, with SES ranks corresponding to household income ranks. The sample for both series is children born between 1980 and 1982. In both samples, children’s SES ranks are based on their ranks within their birth cohort among children linked to parents, while parents’ SES ranks are based on their ranks relative to other parents in the same group of parents linked to children born between 1980–82. We report a slope estimated using a linear regression for each series, with heteroskedasticity-robust standard errors in parentheses.

**Extended Data Fig. 3:**
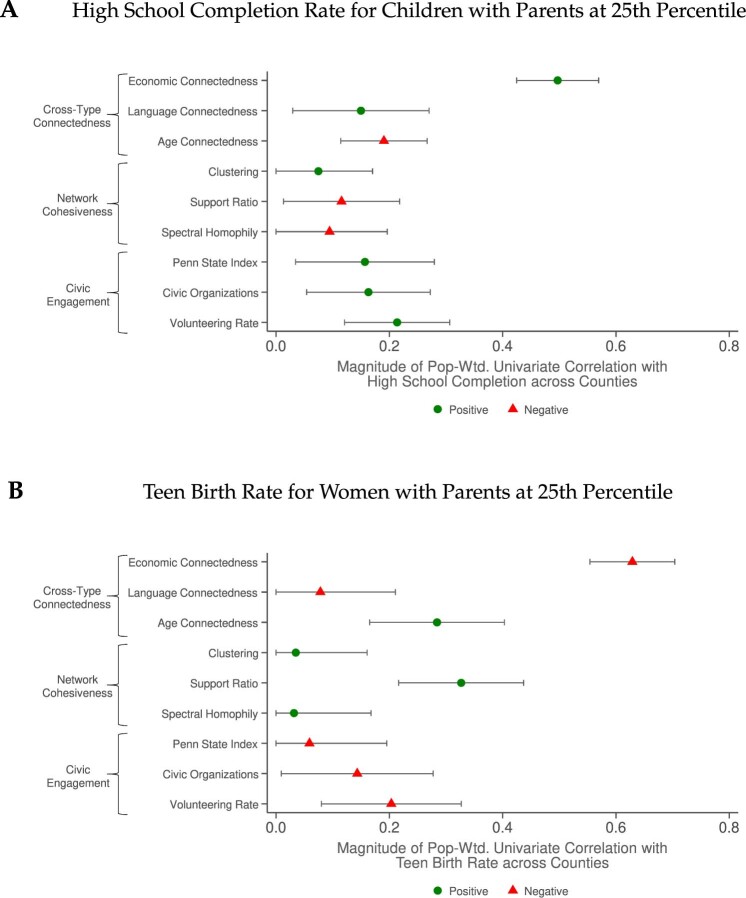
County-Level Univariate Correlations between Other Outcomes and Measures of Social Capital. This figure replicates the across-county correlations shown in Fig. 3a with two different outcome variables: high school completion rates (Panel A) and teen birth rates (Panel B) for children with parents at the 25th percentile of the national income distribution. These outcome variables are obtained from the Opportunity Atlas^72^. See notes to Fig. 3 for further details.

On average, an increase in EC of 0.5 units (equivalent to raising the share of high-SES friends among low-SES people from 25% to 50%, and approximately equal to the difference in EC between the 10th and 90th percentile counties) is associated with an 8.2 percentile increase in children’s incomes in adulthood. This is a large difference: for context, note that children with high-income (above-median) parents end up 17 percentiles higher in the household income distribution on average than children with low-income (below-median) parents (Extended Data Fig. 2). There are similarly strong associations between EC and many other outcomes related to social mobility, such as high school completion rates and teenage birth rates (Extended Data Fig. 3).

Returning to Fig. 3a, other measures of connectedness across groups—between non-English and English speakers or between younger and older individuals—are less strongly associated with upward mobility. Communities with greater connectedness across groups in general do not necessarily have higher levels of upward mobility. Instead, connections across class lines are what appear to matter.

Measures of network cohesion (for example, clustering and support ratios) also do not strongly correlate with observational measures of upward income mobility. This is because there are many areas that exhibit highly cohesive networks—and thus might be thought of as tightly knit communities—but that nevertheless have low levels of EC and correspondingly low levels of upward mobility. A potential explanation for this pattern is that although those communities have strong social connections among their predominantly low-income residents (bonding social capital), they are not well connected to individuals from higher-SES backgrounds who can provide the types of resources, opportunities and information^30,31^ needed to rise economically (bridging social capital).

Finally, we examine associations between economic mobility and measures of civic engagement. The widely used Penn State index^63^ of participation in civic organizations has a correlation of 0.06 across counties with upward mobility. There are similarly weak associations of upward mobility with our measures of the density of civic organizations and volunteering rates. The difference between these findings and previous work that has found stronger associations between civic engagement and economic mobility is primarily because we weight our correlations by the number of children with below-national-median parental income. As a result, rural areas—where civic engagement is more strongly correlated with mobility—receive lower weight in our correlations (Supplementary Information C.2).

When we regress measures of upward mobility on standardized versions of all of the social capital measures together, EC remains the strongest predictor of upward mobility by a significant margin. By contrast, measures of civic engagement and network cohesiveness have coefficients near zero (Fig. 3b). Furthermore, a Lasso regression selects EC as the first social capital measure to include and places greater weight on EC than on other measures (Supplementary Fig. 2a). Moreover, the incremental *R*^2^ of including EC conditional on all the other social capital measures is an order of magnitude larger than the incremental *R*^2^ of including any of the other measures (Supplementary Fig. 2c).

### ZIP-code-level correlations

When studying variation across ZIP codes instead of counties in the United States, we find very similar correlations between upward income mobility and social capital measures (Extended Data Table 2 and Supplementary Fig. 3). In particular, upward mobility is highly correlated (0.69) with EC across ZIP codes (Supplementary Fig. 4), but more weakly correlated with all the other social capital measures. Going from the 10th to the 90th percentile ZIP code in the United States in terms of EC is associated with an 11 percentile increase in the mean adult income rank of children growing up in low-income families. This value is comparable to the 12.6 percentile difference in mean income ranks between Black children and white children with low-income parents^73^.

**Extended Data Fig. 4:**
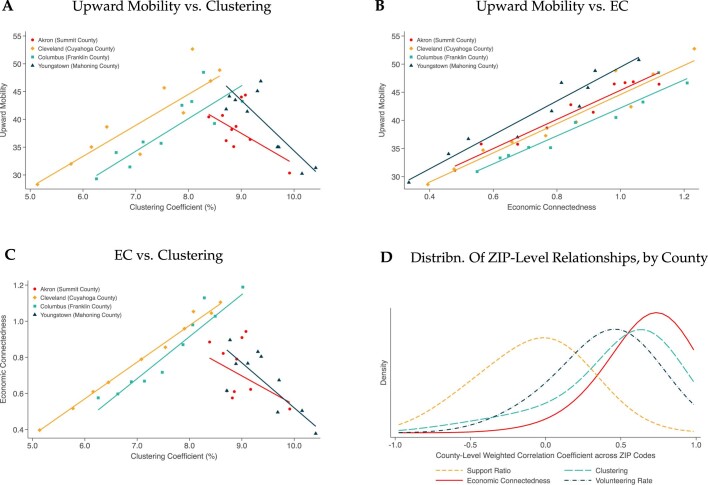
Heterogeneity in Relationships between Upward Income Mobility and Social Capital Measures across Counties. Panel A presents binned scatter plots of upward income mobility against the degree of clustering in networks across ZIP codes in four counties in Ohio: Summit County (Akron), Cuyahoga County (Cleveland), Franklin County (Columbus), and Mahoning County (Youngstown). Clustering is defined as the share of an individual’s friend pairs who are also friends with each other, averaged over all individuals in a ZIP code. Panel B presents analogous ZIP code-level binned scatter plots of upward mobility against economic connectedness. Panel C presents ZIP code-level binned scatter plots of economic connectedness against clustering coefficients. To construct these binned scatter plots, we group ZIP codes within each county into ten (population-weighted) bins based on the relevant social capital measure shown on the horizontal axis and plot the mean (population-weighted) level of the outcome variable against the social capital measure within each bin. Panel D presents kernel density plots of the distribution of ZIP-code-level correlations between upward mobility and several social capital measures across counties for the 250 most populous counties. To construct these distributions, we first estimate correlations between upward income mobility and the social capital measure of interest at the ZIP code level in each county, and then plot the distribution of these correlations. All correlations and distributions are weighted by the number of children whose parents earn less than the national median household income in each ZIP code and county, respectively.

Next, we examine the association between social capital measures and upward mobility across ZIP codes within the same county to assess whether the ZIP-code-level relationships differ across counties. The ZIP-code-level correlation between EC and mobility is strongly positive within nearly all counties. By contrast, there is substantial heterogeneity in the ZIP-code-level relationships between other measures of social capital and mobility across counties. Extended Data Fig. 4a illustrates this by presenting binned scatter plots of the relationship between upward mobility and clustering coefficients by ZIP code across four cities in Ohio: Akron, Cleveland, Columbus and Youngstown. In Cleveland and Columbus, where baseline levels of clustering are relatively low, neighbourhoods with higher clustering coefficients have significantly higher levels of upward income mobility. But in Akron and Youngstown, which generally have higher levels of clustering, clustering and upward mobility are negatively correlated. Hence, it is not that clustering coefficients have no signal in predicting economic mobility; instead, their relationship with mobility varies across places, in part depending on their average levels of clustering.

The relationship between EC and mobility is much more stable across the same four cities, as shown in Extended Data Fig. 4b. The relationships between clustering coefficients and EC closely match those for mobility. In Cleveland and Columbus, clustering coefficients and EC are positively related, whereas in Akron and Youngstown, they are negatively related (Extended Data Fig. 4c). Building on these examples, we find that clustering is often positively correlated with EC and mobility when clustering is low, whereas it is often negatively correlated with both EC and mobility when levels of clustering are high. These patterns suggest that EC may mediate the relationship between  other social capital measures and mobility. That is, the links between other social capital measures and mobility might run through economic connectedness.

Extended Data Fig. 4d generalizes the four examples by plotting the distributions of the correlations between upward mobility and various measures of social capital across ZIP codes within the 250 most populous counties in the United States. For EC, the distribution sharply peaks around 0.7, showing that economic connectedness and mobility are positively correlated across ZIP codes in nearly all counties. By contrast, the other social capital measures exhibit more diffuse distributions across counties. Notably, these differences are not just due to sampling error in the correlations. Adjusting for noise by calculating the reliability of the estimates and the standard deviation of the latent signal distribution produces similar conclusions (Supplementary Table 2).

To summarize, measures of social capital that are based solely on the structure of the network graph (network cohesion) or purely on individuals’ civic behaviours (civic engagement) do not have robust associations with observational measures of economic mobility across areas. Measures that combine data on networks with information on SES have stronger and more stable relationships with economic mobility.

Having established that EC stands out among social capital measures as a strong predictor of economic mobility, in the remainder of the paper we focus on understanding the source of this correlation; that is, why more economically connected areas tend to have higher rates of economic mobility.

## Why EC is related to economic  mobility

There are many theories for why economic connectedness could have a positive causal effect on upward income mobility. For example, economic mobility might be facilitated by connections to people who can shape aspirations or provide access to information and job opportunities^30,32^. This interpretation is consistent with the argument that bridging capital—a concept that encompasses EC—is particularly valuable for ‘getting ahead’^36^. However, there are also many alternative explanations for the correlation between EC and mobility that do not rely on a causal effect of connectedness on mobility. We evaluate three such possibilities in turn—reverse causality, selection effects, and omitted variables—with the broader aim of better understanding the channels through which connectedness and mobility are related.

### Reverse causality

The first alternative explanation for the correlation between connectedness and mobility we consider is reverse causality, whereby greater economic mobility could lead to greater EC. Specifically, in our baseline analysis, we correlated rates of upward income mobility with EC measured among adults. Because friendships and SES are measured in adulthood, economic connectedness may itself be influenced by rates of intergenerational mobility. For example, in places with high upward mobility, many children from low-SES families have high incomes as adults and may retain friendships with individuals who remain at a low SES. This would lead to high-mobility areas having a high rate of friendships among people with different SES in adulthood, even in the absence of any effect of economic connectedness on mobility.

To assess the importance of reverse causality, we examine the association between economic mobility and childhood EC, on the basis of childhood friendships and parental SES. Because childhood friendships are made before people start working, they cannot be directly influenced by rates of economic mobility. We measure childhood EC using two sources of data, each of which has benefits and drawbacks (Methods: ‘Measuring connectedness’). The first is based on the high school friends and parental SES of individuals in our primary Facebook analysis sample. The second uses data from Instagram for individuals aged 13–17 years in 2022, measuring parental SES based on the teenagers’ residential ZIP codes and mobile phone models.

The correlation between upward mobility and childhood EC across counties remains high with both of these measures: 0.44 using parental SES in the Facebook data and 0.62 using the Instagram data (Extended Data Table 2). Since upward mobility remains strongly correlated with childhood EC, any causal effects of mobility on connectedness must account for, at most, a small share of the correlation between the two variables.

### Causal effects of place versus selection

A second potential non-causal explanation for the link between economic connectedness and mobility is selection. Specifically, one might be concerned that the types of families who live in high-EC areas may inherently have higher rates of mobility (for example, because they have more education or wealth), independent of where they live. For example, the types of low-income families who choose to live in high-EC areas may have demographic characteristics or make other choices that increase their children’s rates of upward mobility even in the absence of any causal effect of EC on outcomes.

One of the most salient forms of residential sorting in the United States is segregation by race and ethnicity. Such segregation could lead to a correlation between EC and mobility. For example, areas with larger Black populations tend to have lower levels of EC (Supplementary Table 3). Because Black Americans have lower rates of upward mobility than white Americans^73^—which could be due to factors such as discrimination that are unrelated to differences in EC—differences in racial composition across neighbourhoods could induce a spurious association between EC and mobility when pooling across races.

The simplest way of assessing the importance of differences by race would be to replicate our baseline correlations conditioning on race, for instance by correlating upward mobility and connectedness among Black individuals. As a feasible alternative in the absence of individual-level data on race, we focus on counties or ZIP codes where most of the residents are of the same race (based on publicly available data from the Census). We then correlate race-specific measures of economic mobility^72^ with EC (pooling all racial groups) within these areas.

Extended Data Table 3 reports the results of this analysis. Column 1 shows that the correlation between upward mobility for white individuals and overall EC is 0.68 in counties where at least 80% of residents are white (which have a mean white share of 90%). The correlation is similar (0.69) in counties where at least 90% of residents are white, and the mean white share is 95% (column 2). Columns 3 and 4 show that results are similar at the ZIP-code level. In ZIP codes where at least 90% of residents are white, the correlation between upward mobility and EC is 0.69. Columns 5 and 6 show similarly strong correlations between upward mobility for Black people and EC in ZIP codes in which residents are predominantly Black. Columns 7 and 8 show smaller (although not statistically distinguishable) correlations between upward mobility for Hispanic people and EC in the few ZIP codes in which residents are predominantly Hispanic. Note that we can only perform this analysis at the ZIP-code level for Hispanic and Black individuals because there are very few counties that have more than 80% Black or Hispanic residents.

The results in Extended Data Table 3 show that economic connectedness remains highly correlated with economic mobility even conditional on race, which implies that segregation by race is unlikely to be the primary driver of the observed correlation between EC and mobility overall. Relationships between mobility and other measures of social capital also remain similar when restricting the sample to areas in which one race forms an overwhelming share of the population (Supplementary Fig. 5).

Of course, there are many dimensions beyond race on which families may sort across neighbourhoods, such as their underlying human capital or their propensity to invest in their children’s education. To test for sorting on such dimensions, many of which are unobservable, one would ideally randomly assign families to low-EC and high-EC areas—thereby ensuring that families in high-EC and low-EC areas are comparable—and examine whether their children’s outcomes differ in adulthood. We approximate this experiment using quasi-experimental estimates of the causal effect of growing up for an additional year in each county in the United States on household incomes in adulthood from Chetty and Hendren^74^. That study used variation in the age at which children move across counties to identify the causal effect of growing up in each county for children with parents at the 25th percentile of the income distribution. Under the identification assumption that the timing of moves is unrelated to children’s potential outcomes—an assumption validated in a series of experimental and quasi-experimental studies^75–78^—differences in adult incomes for children who move at younger versus older ages to a given county reveal its causal effect on economic mobility.

**Extended Data Fig. 5:**
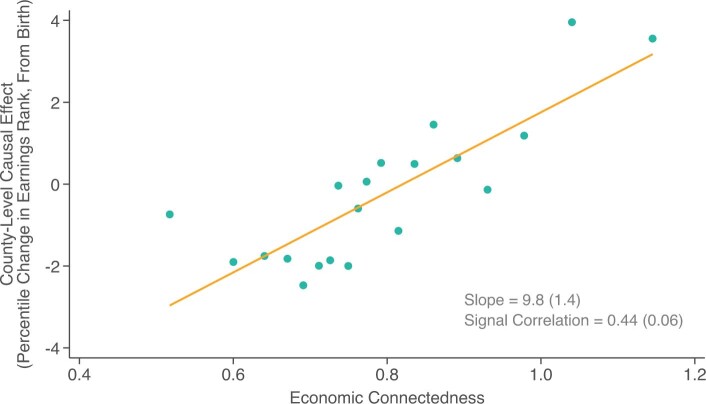
Association between Economic Connectedness and Counties’ Causal Effects on Upward Income Mobility. This figure presents a binned scatter plot of counties’ causal effects on upward mobility against economic connectedness. The binned scatter plot is constructed in the same way as described in the notes to Extended Data Figure 4, using 20 bins of Economic Connectedness instead of 10 and weighting by the precision (inverse of standard error squared) of the causal effect estimates. Causal effects on upward mobility are the annual exposure effect estimates constructed by Chetty and Hendren^74^ by analyzing cross-county movers. These annual exposure effects are multiplied by 20 so that they can be interpreted as the causal effect of growing up in a given location from birth to age 20 on an individual’s household income percentile rank in adulthood. The slope is estimated using an OLS regression of the causal effect estimates on EC, weighting by the precision of the causal effect estimates. The signal correlation is calculated by dividing the raw (precision-weighted) correlation between the causal effects and EC by the square root of the precision-weighted reliability of the estimated causal effects.

We use Chetty and Hendren's estimates to analyse the relationship between the causal effects of counties on upward mobility and EC. We measure the causal effect of each county as the mean change in an individual’s percentile income rank from growing up from birth (for 20 years) in that county instead of the average county in the United States^75^. Extended Data Fig. 5 presents a binned scatter plot of the causal effects of counties on upward mobility against their EC. Higher EC counties have larger causal effects on upward mobility, with a correlation of 0.44 (s.e. = 0.06) after correcting for sampling error in the causal effect estimates (Methods: ‘Correlations’). In a multivariable regression of counties' causal mobility effects on all our social capital measures, EC remains highly correlated with causal effects on mobility. By contrast, most other social capital measures do not exhibit significant associations (Supplementary Fig. 6).

The slope of the relationship shown in Extended Data Fig. 5 implies that growing up from birth in a county with 1 unit higher EC increases income in adulthood by 9.8 percentiles (a 30.7% increase relative to mean income ranks) for children of parents with low income. This estimate implies that moving at birth from the 10th to 90th percentile ZIP code in terms of EC—a move associated with an increase in EC of 0.57—would increase children’s household income in adulthood by 17.5% on average. As another benchmark, note that the average difference in EC between low- and high-SES individuals is 0.636. If low-SES children were to grow up in counties with EC comparable to the average high-SES child, their incomes would increase on average by 0.636 × 30.7 = 19.5% (equivalent to 6.23 percentiles). This increase in income would close about 37% of the current 17 percentile gap in income in adulthood between children with parents at the 25th and 75th percentiles of the income distribution.

We conclude that the correlation between EC and mobility is not driven simply by differences in the types of families who live in high EC areas. Instead, growing up in an area with higher EC causes significantly higher rates of upward mobility.

### Connectedness versus other factors

Higher EC areas may generate higher levels of mobility for two reasons: either economic connectedness itself has a causal effect on mobility or high-EC places have other characteristics (for example, better schools) that generate higher levels of mobility. As a step towards distinguishing these two explanations, we compare the relative explanatory power of EC and the strongest neighbourhood-level predictors of economic mobility identified in previous work.

We begin by analysing incomes across neighbourhoods. Several studies have shown that areas with lower incomes and more highly concentrated poverty have lower rates of economic mobility^11,79^. Motivated by such findings, many place-based policies use high poverty rates as a marker to identify low-opportunity neighbourhoods that are eligible for special tax credits and resources, and recent work has sought to help families move to lower-poverty neighbourhoods to improve their economic prospects^80^.

**Fig. 5:**
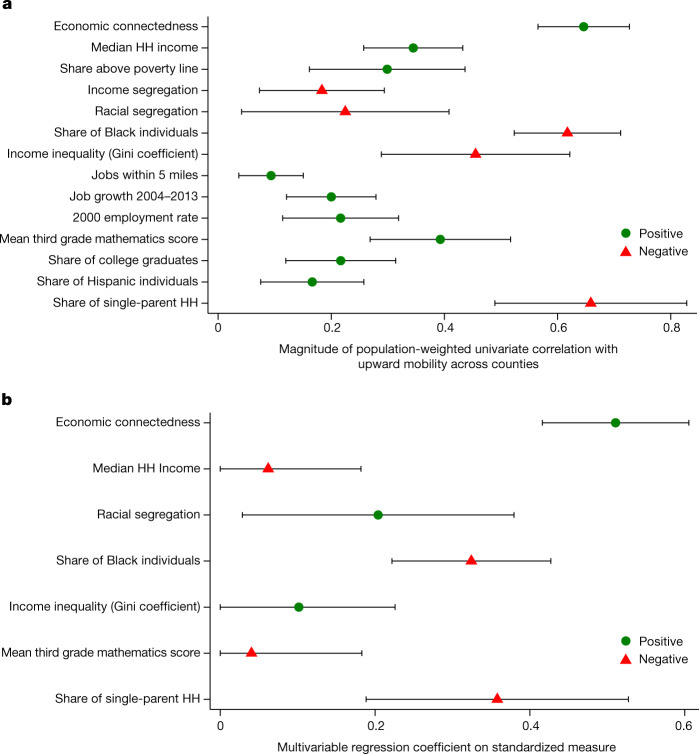
County-level correlations between upward income mobility and neighbourhood characteristics. **a**, County-level univariate correlations of upward income mobility with economic connectedness (EC) and other county characteristics obtained from external datasets (see Supplementary Information A.5 for details). Upward income mobility is obtained from the Opportunity Atlas^72^ and is measured as the predicted household (HH) income rank in adulthood for children in the 1978–1983 birth cohorts with parents at the 25th percentile of the national income distribution. Income segregation is defined using a Theil (entropy) index^81^. Racial segregation is defined using Theil's H-index across four groups (white, Black, Hispanic, other). See Supplementary Information A.5.1 for details. The Gini coefficient is defined as the raw Gini coefficient estimated using tax data minus the income share of the top 1% to obtain a measure of inequality among the bottom 99% in each county^10^. The rest of the variables are all obtained from the Opportunity Atlas^72^. Test scores are measured in third grade, which includes children who are 8 to 9 years old. **b**, Estimates from a single multivariable regression of upward mobility on a subset of variables from **a**, with both the outcome and dependent variables standardized to have a mean of zero and a standard deviation of one. The variables used in **b** are the seven variables from **a** that have the largest univariate correlations with upward mobility (except the share of households above the poverty line, which is highly correlated with median household incomes), which include all of the strongest predictors of mobility identified in prior work^10^. All correlations and regressions are weighted by the number of children in each county whose parents have below-national-median income. Intervals represent 95% confidence intervals calculated using standard errors clustered by commuting zone.

Figure 5a shows univariate county-level correlations between upward mobility and measures of income and various other neighbourhood characteristics (results at the ZIP-code level, shown in Supplementary Fig. 4b, are similar). The share of individuals above the poverty line and median household incomes have correlations of 0.3–0.35 with  upward mobility across counties. When we regress upward income mobility on both EC and measures of local income levels (poverty rates or median household incomes), connectedness remains a strong predictor of upward mobility. By contrast, measures of local income levels lose much of their predictive power at both the county and ZIP code levels (Table 2 (EC versus median income and poverty rates) and Supplementary Figs. 7 and 8).

**Table 2:** Associations between upward income mobility, EC and other neighbourhood characteristics

EC versus median income and poverty rates								
Dependent variable	Upward income mobility							
	Counties				ZIP codes			
	(1)	(2)	(3)	(4)	(5)	(6)	(7)	(8)
Median income	0.345***	–0.006	–	–	0.574***	0.209***	–	–
	(0.045)	(0.069)	–	–	(0.023)	(0.029)	–	–
Poverty rate	–	–	-0.299***	0.142**	–	–	–0.543***	-0.195***
	–	–	(0.070)	(0.069)	–	–	(0.052)	(0.054)
Economic connectedness	–	0.649***	–	0.732***	–	0.548***	–	0.568***
	–	(0.058)	–	(0.043)	–	(0.038)	–	(0.033)
Observations	2,984	2,984	2,984	2,984	24,165	24,165	24,165	24,165
*R*^2^	0.119	0.418	0.089	0.430	0.330	0.496	0.295	0.496

EC versus segregation and inequality						
Dependent variable	Upward income mobility					
	(1)	(2)	(3)	(4)	(5)	(6)
Income segregation	–0.173***	-0.071	–	–	–	–
	(0.053)	(0.054)	–	–	–	–
Racial segregation	–	–	–0.212**	-0.027	–	–
	–	–	(0.088)	(0.086)	–	–
Income inequality (Gini coefficient)	–	–	–	–	–0.449***	-0.103
	–	–	–	–	(0.084)	(0.091)
Economic connectedness	–	0.601***	–	0.604***	–	0.577***
	–	(0.044)	–	(0.054)	–	(0.063)
Observations	1,820	1,820	1,821	1,821	2,741	2,741
*R*^2^	0.034	0.413	0.051	0.408	0.207	0.424

EC versus share of Black residents								
Dependent variable	Upward income mobility for Black individuals				Upward income mobility for white individuals			
	Counties		ZIP codes		Counties		ZIP codes	
	(1)	(2)	(3)	(4)	(5)	(6)	(7)	(8)
Share of Black individuals	–0.158**	0.078	–0.204***	–0.014	–0.128**	0.151**	–0.250***	0.035*
	(0.068)	(0.076)	(0.057)	(0.071)	(0.057)	(0.067)	(0.018)	(0.018)
Economic connectedness	–	0.502***	–	0.468***	–	0.582***	–	0.631***
	–	(0.095)	–	(0.083)	–	(0.051)	–	(0.027)
Observations	1,885	1,885	11,147	11,147	2,982	2,982	24,020	24,020
*R*^2^	0.025	0.222	0.042	0.224	0.016	0.277	0.063	0.380

This table presents estimates from ordinary least squares (OLS) regressions of upward income mobility on economic connectedness (EC) and other area-level characteristics. Upward income mobility is obtained from the Opportunity Atlas^72^ and is measured as the predicted household income rank in adulthood for children in the 1978–1983 birth cohorts with parents at the 25th percentile of the national income distribution. EC is twice the share of above-median-SES friends among below-median-SES people. We standardize every dependent and independent variable to have a mean of zero and variance of one (weighted by the number of children with below-median parental income in the county). For ‘EC versus median income and poverty rates’ and ‘EC versus segregation and inequality’, the dependent variables are upward mobility pooling all racial and ethnic groups^72^, and regressions are weighted by the number of children with below-median parental income. ‘EC versus median income and poverty rates’ presents regressions at both the county and ZIP code levels, with median household income and poverty rates by county and ZIP code obtained from the 2000 Census. In ‘EC versus segregation and inequality’, all regressions are estimated at the county level. Income segregation is defined using a Theil (entropy) index^81^. Racial segregation is defined using Theil’s H-index across four groups (white, Black, Hispanic, other); see Supplementary Information A.5.1 for details. Gini coefficients are defined as the raw Gini coefficient estimated using tax data minus the income share of the top 1% to obtain a measure of inequality among the bottom 99% in each county^10^. ‘EC versus share of Black residents’ presents regressions at both the county and ZIP code levels. The dependent variables are upward mobility estimates for Black and white individuals separately^74^. ‘Share of Black individuals’ is from the 2000 Census. All regressions in this section are weighted by the race-specific number of children with below-median parental income in the county. See Supplementary Information A.5 for further details on data sources for neighbourhood-level characteristics. Standard errors (reported in parentheses) are clustered at the commuting zone level. Asterisks indicate the level of significance: *10%, **5%, ***1%.

**Fig. 6:**
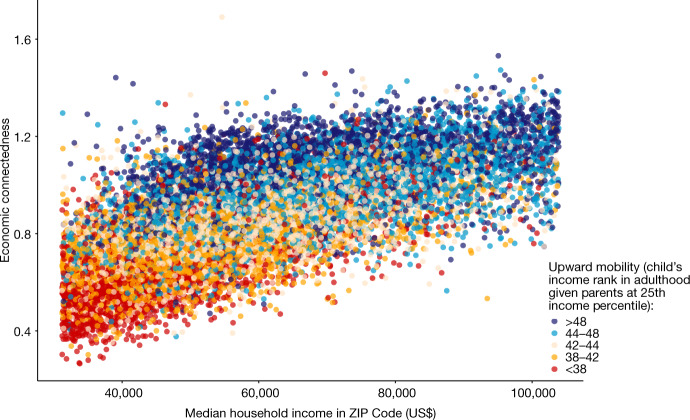
Associations between upward income mobility, EC and median household income by ZIP code. Scatter plot of economic connectedness (EC) against median household income (based on the 2014–2018 ACS) by ZIP code. EC is defined as twice the share of above-median-SES friends among below-median-SES individuals. The points are coloured by the level of upward income mobility for children who grew up in the ZIP code. Upward income mobility is obtained from the Opportunity Atlas^72^ and is measured as the predicted household income rank in adulthood for children in the 1978–1983 birth cohorts with parents at the 25th percentile of the national income distribution.

These findings suggest that EC may be a mediator through which concentrated poverty affects upward mobility. That is, living in a lower income neighbourhood may inhibit upward mobility insofar as it reduces interaction with higher SES people, but does not appear to have a strong influence beyond its effect on EC. Figure 6 demonstrates this point more directly by presenting a scatter plot of EC against median household income by ZIP code. The dots are coloured according to the level of upward income mobility for children who grew up in low-income families in that ZIP code, with blue representing areas with higher levels of upward mobility and red representing areas with lower levels of mobility. Horizontal slices of the graph—neighbourhoods with different levels of median income but comparable levels of EC—tend to have similar levels of economic mobility. By contrast, vertical slices of the graph—areas with comparable incomes but different levels of EC—transition from low to high economic mobility as EC rises. These results imply that it is growing up in an area with high EC—rather than just around high-income people—that leads to better prospects for upward mobility.

**Extended Data Fig. 6:**
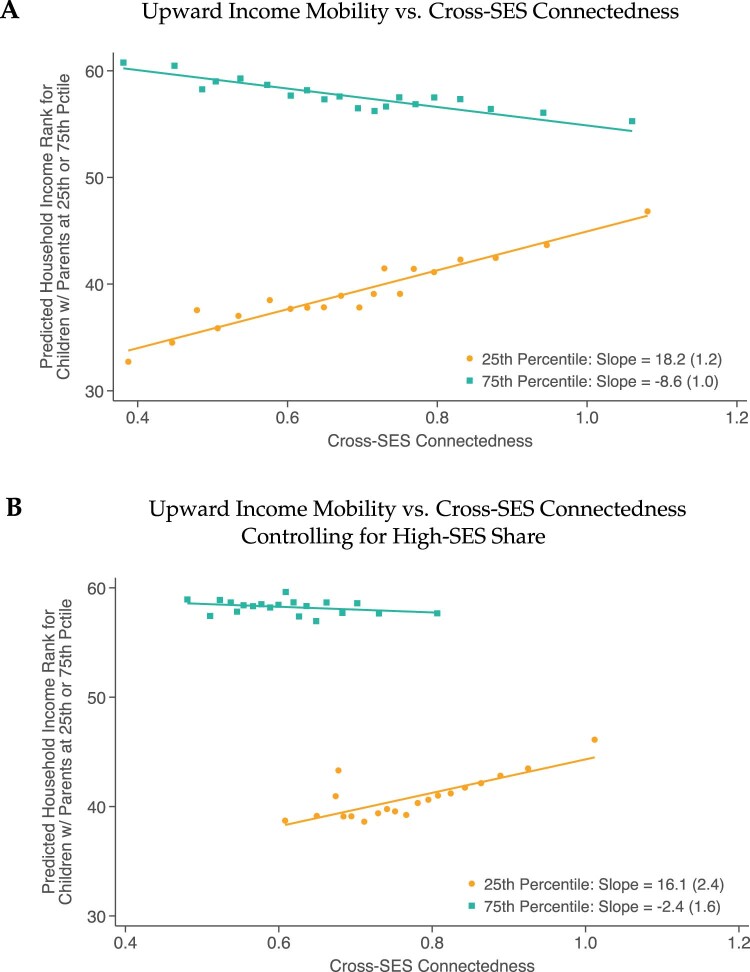
Associations between Upward Income Mobility and Economic Connectedness for Low-SES and High-SES Individuals. This figure presents binned scatter plots of children’s predicted income ranks in adulthood against cross-SES connectedness by county, separately for children with low-income (25th percentile) parents and high-income (75th percentile) parents. Data on children’s outcomes are obtained from the Opportunity Atlas^72^. We define cross-SES connectedness as the normalized share of friends for an individual in one SES group who belong to the other SES group. For below-median SES individuals, cross-SES connectedness is the same as our baseline definition of economic connectedness. Hence, the series in orange circles in Panel A is a binned scatter plot analog of Fig. 4, pooling data from all counties (see notes to Extended Data Figure 4 for details on construction of binned scatter plots). For above-median-SES individuals, cross-SES connectedness is twice the share of their friends who are low-SES. Panel B replicates Panel A, controlling for the share of high-SES individuals in each county. The series in Panel B are constructed by first residualizing predicted household income ranks and cross-SES connectedness on the share of high-SES people using univariate OLS regressions, and then constructing a binned scatter plot of the residuals after adding back the means of each variable for scaling purposes. We report estimates of the slope of each series based on OLS regressions with standard errors, clustered by commuting zone, in parentheses.

Although local income levels explain little of the relationship between EC and outcomes for children starting out in low-income (25th percentile) families, they do appear to mediate the relationship between connectedness and outcomes for children from high-income (75th percentile) families. We illustrate this result in Extended Data Fig. 6a. As a reference, the series in orange circles presents a county-level binned scatter plot of upward mobility against EC for individuals with low SES. This series is similar to the scatter plot in Fig. 4, except that we include all counties and group them into 20 equal-sized bins on the basis of their level of EC to show the conditional expectation of upward mobility given EC non-parametrically. Consistent with the pattern in Fig. 4, there is a strong positive slope of 18.2.

Now consider the relationship between the average income ranks in adulthood of children with parents at the 75th percentile and the share of low-SES friends that high-SES individuals have. This relationship (plotted in blue circles) is flatter than that for low-SES individuals. A 1 unit increase in cross-group connectedness—defined here as twice the share of low-SES friends among high-SES individuals—is associated with an 8.6 percentile reduction in mean income rank for children with parents at the 75th percentile. Notably, after controlling for the share of high-SES individuals in the county, greater cross-group EC remains strongly positively associated with outcomes for children with parents at the 25th percentile (as established above), but is now uncorrelated with the economic outcomes for children with parents at the 75th percentile (Extended Data Fig. 6b). A potential explanation for this pattern is that greater interaction between low-SES and high-SES households conditional on the income mix in an area benefits lower-income people without harming those with higher incomes; however, greater income mixing (integration) benefits lower-income people partly at the expense of higher-income people by redistributing public goods (for example, local public school funding) from people with higher incomes to people with lower incomes. These results raise the possibility that more economically connected communities can benefit lower-income households with limited adverse impacts on those with higher incomes, particularly if increasing cross-SES connections does not require changing the income mix or resources in an area.

Going beyond average income levels, previous research has also shown that in counties where people of different incomes or racial backgrounds live in separate neighbourhoods, levels of economic mobility are generally lower. Indices of segregation by income and race (constructed from Census data using standard methods^81^; Supplementary Information A.5.1) have negative correlations of 0.17–0.21 with economic mobility across counties, significantly lower than the correlation of 0.65 observed with EC. Hence, using network data to directly measure interaction (rather than using residential location as a proxy) adds considerable explanatory power for understanding economic mobility. Moreover, when we regress upward mobility on both EC and segregation measures, connectedness remains a strong predictor of upward mobility. By contrast, the segregation indices lose their predictive power (Table 2 (EC versus segregation and inequality) and Supplementary Fig. 9).

Previous work has established that Black individuals living in neighbourhoods with a larger Black population have poorer educational and economic outcomes on average^12^. We replicate these results in the odd-numbered columns of Table 2 (EC versus share of Black residents) by regressing upward income mobility for Black people and white people on the share of Black residents in an area (for both counties and ZIP codes). The corresponding even-numbered columns show that controlling for EC eliminates or even reverses the relationship between the share of Black residents and rates of upward mobility (Supplementary Fig. 10). Areas with a larger Black population tend to have lower levels of EC (Supplementary Table 3), and this relationship accounts for the negative correlation between the share of Black residents and rates of mobility.

Research has also found a strong negative correlation between income inequality within a generation (measured, for example, using the Gini coefficient) and upward mobility across generations, coined the ‘Great Gatsby curve’^13,14^. Controlling for EC essentially eliminates this relationship (columns 5 and 6 of Table 2 (EC versus segregation and inequality) and Supplementary Fig. 9). Greater income inequality is associated with less EC, and that relationship largely explains the negative correlation between inequality and mobility. In short, a lack of economic connectedness may be a key reason that upward mobility is lower in areas with larger Black populations and greater inequality^82^. 

Finally, we turn to other factors that have been explored in previous work, ranging from the quality of local schools to job availability to measures of family structure. EC is more strongly correlated with upward economic mobility than almost all of those characteristics in univariate specifications (Fig. 5a). We also estimate a multivariable regression of upward mobility on EC along with other predictors that have the highest univariate correlations with mobility. In this analysis, EC is the strongest predictor of upward mobility (Fig. 5b) and has the largest incremental *R*^2^ (Supplementary Fig. 2d). EC is also among the first variables—along with the share of single parents—that are chosen by a Lasso regression as predictors of economic mobility (Supplementary Fig. 2b).

In summary, places with higher levels of EC generate higher levels of economic mobility, even when controlling for the strongest neighbourhood-level predictors of economic mobility identified in prior research. Moreover, the relationships between these other neighbourhood characteristics and mobility become much weaker once we control for EC, which indicates that the links between those factors and mobility may run through their impacts on EC. These findings suggest that other observable neighbourhood characteristics do not explain why higher EC areas generate higher levels of upward mobility, calling for further focus on causal mechanisms through which economic connectedness itself may affect mobility.

## Discussion

Measuring social capital has proven to be more challenging than measuring other forms of capital, such as financial or human capital. Data from online social networking platforms offer a path to solving this problem. The new measures of social capital constructed here provide a rich picture of how social capital varies across areas in the United States. Different notions of social capital—connectedness across socioeconomic lines, the cohesiveness of a community and civic engagement—exhibit highly different spatial patterns. Many communities are rich in one form of social capital but poor in others.

Distinguishing these forms of social capital is important because some types of social capital are more correlated with certain outcomes than others. For instance, economic connectedness (EC)—the share of high-SES friends among low-SES people—is strongly associated with upward income mobility, whereas other forms of social capital are not. Areas with higher EC have large positive causal effects on children’s prospects for upward mobility. We caution, however, that this finding does not imply that EC is the best or most important measure of social capital in general. EC may be the best predictor of economic mobility because mobility is essentially a measure of the degree to which individuals can increase their own SES, making it natural that links to higher-SES individuals are related to that outcome. This is consistent with hypotheses that bridging capital is useful specifically for getting ahead (rather than simply getting by)^36,83^. For other outcomes, other social capital indices that we construct here may be stronger predictors. For example, differences in life expectancy among individuals with low income across counties are more strongly predicted by network cohesiveness measures (clustering coefficients and support ratios) than EC (Supplementary Fig. 11 and Supplementary Information C.3).

Our analysis raises three sets of questions for future research. First, it would be useful to conduct systematic studies of the forms of social capital that matter for other outcomes; for example, to determine which forms of social capital matter for health behaviours or the formation of political preferences. The publicly available statistics constructed here can be used to study many such questions.

Second, it would be valuable to build on the methods developed here and construct analogous measures of social capital beyond the United States, either using social network data or other sources of network information such as financial transactions or mobile phone data^84^. Although many of the lessons obtained from our analysis of the United States are likely to generalize more broadly, international comparisons would enrich our understanding of social capital and its determinants.

Finally, it would be useful to directly study whether efforts to increase economic connectdness can increase intergenerational income mobility. Doing so requires an understanding of the determinants of EC and potential interventions to increase it. We address these questions in the companion paper^9^, in which we study why economic connectedness varies with SES and how we can increase connectedness among individuals with low SES.

## Methods

### Sample construction

This section describes the methods used to generate the data analysed in this paper. A server-side analysis script was designed to automatically process the raw data, strip the data of personal identifiers, and generate aggregate results, which we analyzed to produce the conclusions in this paper. The script then promptly deleted the raw data generated for this project (see the Privacy and Ethics section).

We work with privacy-protected data from Facebook. Survey data show that more than 69% of the US adult population used Facebook in 2019, and about three-quarters of those individuals did so every day^37^. The same survey also found that Facebook usage rates are similar across income groups, education levels and racial groups, as well as among urban, rural and suburban residents; they are lower among older adults and slightly higher among women than men.

Starting from the raw Facebook data as of 28 May 2022, our primary analysis sample was constructed by limiting the data to users aged between 25 and 44 years who reside in the United States, were active on the Facebook platform at least once in the previous 30 days, had at least 100 US-based Facebook friends and had a ZIP code. Our final analysis sample consists of 72.2 million Facebook users who constitute 84% of the US population between ages 25 and 44 years (based on a comparison to the 2014–2018 American Community Survey (ACS)). We focus on the 25–44-year age range because previous work^37^ has documented that its Facebook usage rate is above 80%, higher than for other age groups. In addition, the ACS publicly releases demographic data for certain age groups, one of which is ages 25–44 years, which enables us to compare our sample with the full population as well as to use ACS aggregates to predict SES (‘Variable definitions’).

We do not link any external individual-level information to the Facebook data. However, we use various publicly available sources of aggregate statistics to supplement our analysis, including data on median incomes by block group from the 2014–2018 ACS, data on economic mobility by Census tract and county from the Opportunity Atlas^72^, and measures of county-level and ZIP-level characteristics, such as the share of the population by race and ethnicity and the share of single parents, from the ACS and the Census. We describe those data in detail in Supplementary Information A.5.

### Variable definitions

We construct the following sets of variables for each person in our analysis sample. We measured these variables on 28 May 2022.

#### Friendship links

The data contain information on all friendship links between Facebook users. We focus only on friendships within our analysis sample; that is, we exclude friendships with people aged below 25 years or above 44 years, people who live outside the United States or people who do not satisfy one of our other criteria for inclusion in the analysis sample.

Facebook friendship links need to be confirmed by both parties, and most Facebook friendship links are between individuals who have interacted in person^85^. The Facebook friendship network can therefore be interpreted as providing data on people’s real-world friends and acquaintances rather than purely online connections. Because individuals tend to have many more friends on Facebook than they interact with regularly, we also verify that our results hold when focusing on an individual’s ten closest friends, where closeness is measured on the basis of the frequency of public interactions such as likes, tags, wall posts and comments.

#### Locations

Following prior work^86^, we use location data to construct statistics at various geographical levels. Every individual is assigned a residential ZIP code and county based on information and activity on Facebook, including the city reported on Facebook profiles as well as device and connection information. Formally, we use 2010 Census ZIP code tabulation areas (ZCTAs) to perform all geographical analyses of ZIP-code-level data. We refer to these ZCTAs as ZIP codes for simplicity. According to the 2014–2018 ACS, there are 219,214 Census block groups, 32,799 ZIP codes and 3,220 counties, with average populations of 1,488, 9,948 and 101,332 in each respective geographical designation.

#### Socioeconomic status

We construct a model that generates a composite measure of socioeconomic status (SES) for working-age adults (individuals between the ages of 25 and 64 years) that combines various characteristics. We construct our baseline SES measure in three steps, which are described in greater detail in Supplementary Information B.1.

First, for Facebook users who have location history (LH) settings enabled, we use the ACS to collect the median household income in their Census block group. LH is an opt-in setting for Facebook accounts that allows the collection and storage of location signals provided by a device’s operating system while the app is running. We observe Census block groups from individuals in the LH subsample. By contrast, we can only assign ZIP codes to individuals who do not have LH enabled. If an individual subsequently opts out of LH, their previously stored location signals are not retained.

Second, we estimate a gradient-boosted regression tree to predict these median household incomes using variables observed for all individuals in our sample, such as age, sex, language, relationship status, location information (ZIP code), college, donations, phone model price and mobile carrier, usage of Facebook on the Internet (rather than a mobile device), and other variables related to Facebook usage listed in Supplementary Table 4. We use this model to generate SES predictions for all individuals in our sample.

Finally, individuals (including the LH users in the training sample) are assigned percentile ranks in the national SES distribution on the basis of their predicted SES relative to others in the same birth cohort.

We do not use any information from an individual’s friends to predict their SES, which ensures that errors in the SES predictions are not correlated across friends, which would bias our estimates of homophily by SES. We also do not use direct information on individuals’ incomes or wealth, as we do not observe these variables at the individual level in our data. However, we show below that our measures of SES are highly correlated with external measures of income across subgroups.

The algorithm described above is one of many potential ways of combining a set of underlying proxies for SES into a single measure. To verify that our findings are not sensitive to the specific variables or algorithm used to predict SES, we show that our results are similar when we use a simple unweighted average of *z*-scores of the underlying proxies or when we directly use ZIP code median household incomes for all users, eschewing the prediction model and other proxies entirely (Supplementary Table 5).

#### Parental SES

We link individuals in our primary analysis sample to their parents (who may not be in the analysis sample themselves) to construct measures of family SES during childhood. To link individuals to their parents, we use self-reported familial ties, a hash of user last names, and public user-generated wall posts and major life events (see Supplementary Information A.2 for details). We then use the SES of parents, constructed using the algorithm described above, to assign parental SES to individuals. Finally, we assign individuals a parental SES rank on the basis of their predicted parental SES, ranking individuals on the basis of parental SES relative to others in the same birth cohort. We are able to assign parental SES ranks for 31% of the individuals in our primary analysis sample.

#### High school friendships

To identify friendships made in high school, we first use self-reports to assign individuals to schools. For people who do not report a high school, we use data on their friendship networks to impute those groups (see Supplementary Information A.3 for details). For the 3.3% of users who report multiple high schools, we select the school in which the user has the largest number of friends. This process produces information on high schools for 74.9% of individuals in our analysis sample. Finally, if an individual and one of their friends attended the same high school within three cohorts of each other, we identify them as high school friends.

### Benchmarking

Extended Data Table 4a shows summary statistics for our baseline sample and, for comparison, for those aged between 25 and 44 years in the 2014–2018 ACS. The Facebook sample is similar to the full population in terms of age, sex and language. Consistent with previous work^87^, women are slightly over-represented in our Facebook sample (53.6%) relative to men. The median individual in our analysis sample has 382 in-sample Facebook friends; in total, there are just under 21 billion friendship pairs between individuals in the sample.

As much of our analysis relies on variation across areas, it is important that our sample has good coverage not just nationally but also across locations. In Supplementary Information A.1, we show that our sample has high coverage rates across the United States, and that coverage rates do not vary systematically across locations with different income levels or demographic characteristics.

Most of our analysis draws on the SES measure constructed as described in the previous subsection. We evaluate the accuracy of this SES measure by correlating the share of households with above-median income within each ZIP code from the ACS with the estimated share of Facebook users with above-median SES in our sample. The population-weighted correlation between our estimates of the share of high-SES individuals and the ACS estimates at the ZIP-code level is 0.88. Furthermore, there are similarly high correlations between our estimates of the share of high-SES households and corresponding statistics drawn from external publicly available administrative datasets at the high school and college levels (see the companion paper^9^ for details).

For some parts of our analysis—in particular, for computing measures of EC during childhood—we focus on the subsample of individuals whom we can link to parents with an SES prediction and whom we can assign to a high school on the basis of self-reports and network-based imputations. Panel B of Extended Data Table 4 presents summary statistics for this subsample of 19.4 million users, or about 27% of the full analysis sample. The characteristics of this subsample are broadly similar to those of the full sample, although users whom we can link to high schools and parents with SES predictions are about 2 years younger on average than users in the full sample, in large part because our approach does not allow us to assign SES predictions for parents older than 65 years. County-level median household incomes differ by $876 between the samples, about 6% of a standard deviation.

We further evaluate our SES measure and parental linkages by comparing estimates of intergenerational economic mobility using our SES proxies to publicly available estimates based directly on household incomes from population-level tax data. There is a linear relationship between individuals’ and their parents’ SES ranks across the distribution of parental SES, with a slope of 0.32 (Extended Data Fig. 2) This relationship is similar to the estimated slope of 0.34 in population tax data^10^, thereby supporting the validity of both our SES imputations and parental linkages.

We conclude that our Facebook analysis samples are representative of the populations we seek to study and that our measures of SES align with external data.

### Measuring connectedness

#### Economic connectedness

Let

1 $${f}_{Q,i}\equiv \frac{{[{\rm{N}}{\rm{u}}{\rm{m}}{\rm{b}}{\rm{e}}{\rm{r}}{\rm{o}}{\rm{f}}{\rm{f}}{\rm{r}}{\rm{i}}{\rm{e}}{\rm{n}}{\rm{d}}{\rm{s}}{\rm{i}}{\rm{n}}{\rm{S}}{\rm{E}}{\rm{S}}{\rm{q}}{\rm{u}}{\rm{a}}{\rm{n}}{\rm{t}}{\rm{i}}{\rm{l}}{\rm{e}}Q]}_{i}}{{\rm{T}}{\rm{o}}{\rm{t}}{\rm{a}}{\rm{l}}\,{\rm{n}}{\rm{u}}{\rm{m}}{\rm{b}}{\rm{e}}{\rm{r}}\,{\rm{o}}{\rm{f}}\,{{\rm{f}}{\rm{r}}{\rm{i}}{\rm{e}}{\rm{n}}{\rm{d}}{\rm{s}}}_{i}}$$

denote individual *i*’s share of friends from SES quantile *Q*. To obtain measures of the degree of homophily that are not sensitive to the size of each quantile bin, we normalize *f*_*Q*,*i*_ by the share of individuals in the sample who belong to quantile *Q*, *w*_*Q*_ (for example, *w*_*Q*_ = 0.1 for deciles). We then define person *i*’s individual EC (IEC) to individuals from quantile *Q* as

2 $${{\rm{IEC}}}_{Q,i}\equiv \frac{{f}_{Q,i}}{{w}_{Q}}.$$

We define the level of EC in community (county or ZIP code) *c* as the mean level of individual EC of low-SES (for example, below-median) members of that community, as follows:

3 $${{\rm{EC}}}_{c}=\frac{{\sum }_{i\in L\cap c}{{\rm{IEC}}}_{i}}{{N}_{Lc}},$$

where *N*_*Lc*_ is the number of low-SES individuals in community *c*. When defining EC in a given community, we continue to rank individuals in the national SES distribution and include friendships to individuals residing outside that community. In the presence of homophily, EC ranges from 0 to 1, with a value of 1 indicating, for example, that half of below-median-SES individuals’ friends have above-median-SES.

We construct standard errors for EC in each location using a bootstrap resampling method that adjusts for correlations in connectedness across individuals arising from having common pools of friends (Supplementary Information B.3). Because sample sizes are large, almost none of the geographical difference in EC is due to sampling variation. At the county level, the mean standard error of 0.004 is more than an order of magnitude smaller than the signal standard deviation of EC across counties of 0.18. When we randomly split the microdata into two halves and estimate ECs by county in each half, we obtain a split-sample correlation (reliability) of 0.999 across counties, weighting by the number of people in each county with household income below the national median. The ZIP-code-level estimates we release are also precise, with a split sample reliability of 0.99 (pooling all ZIP codes in the United States) when weighted by below-median-income population.

#### Childhood EC

We construct two measures of childhood EC: one based on links between individuals and their parents in our Facebook analysis sample and another based on data from Instagram.

To measure childhood EC in the Facebook sample, we restrict the sample to individuals whom we could link to high schools and their parents (about 27% of the full sample). We assign parental SES ranks (estimated using the machine-learning algorithm described in the ‘Variable definitions’ section) within this subsample, ranking individuals on the basis of parental SES relative to others in the same birth cohort. We then measure *f*_*Q*,*i*_ as the share of friends from parental-SES quantile *Q* within the subset of friends from high school: friends who attended the same high school and are within three cohorts of the individual (so that they would have most likely overlapped in school). Ideally, we would directly observe all friendships made during childhood. However, because the Facebook platform was not available when the members of the birth cohorts we analyse were growing up, we use current friends who attended the same high school to identify friendships made in childhood. When calculating childhood EC by location, we assign individuals to the counties where their high schools are located, rather than counties where they currently live, to map people to the places where they grew up. We do not produce ZIP-code-level measures of childhood EC because we cannot reliably infer individuals’ childhood ZIP codes from the locations of their high schools (as children from many ZIP codes might attend a given school).

To measure childhood EC for users of Instagram, a widely used social networking platform owned by Meta, we restrict the raw Instagram data to personal users (not business pages) in the United States who had not deactivated their account, been active on the platform within the past 30 days, and were predicted to be between 13 and 17 years of age as of 28 May 2022 (see Supplementary Information A.4 for further details). Next, we assign the individuals in our sample to ZIP codes on the basis of their IP address and other features. Then, we assign Instagram users an SES estimate on the basis of two variables: (1) the median household income of their residential ZIP code from publicly available data on incomes in the 25–44-year age bin from the 2014–2018 ACS, and (2) the price of their phone. We then construct a weighted *z*-score of these two inputs, placing two-thirds of the weight on median household income and one-third of the weight on the price of the phone. The higher weight on ZIP-code-based income relative to phone price reflects that ZIP codes played a particularly large role in the machine-learning model used to construct our baseline measures of SES in the Facebook data (although using other weights in the construction of the *z*-score produced similar results). We rank users nationally on the basis of these weighted *z*-scores to assign them a SES percentile rank. Users above the 50th percentile are termed high SES, whereas those at the 50th percentile and below are termed low SES. Finally, we construct measures of individual EC as defined in equation (2). Because ties on Instagram, which are termed ‘follows’, are directional—that is, one person can follow another without that person following them—we restrict our attention to reciprocal followers to mimic friendships on Facebook when measuring connectedness.

Each of the two measures of childhood EC has certain advantages and limitations. The Facebook parental SES measure has the advantage of capturing the childhood friendships of individuals in approximately the same set of cohorts for which we measure economic mobility. However, because we are able to construct this measure only for the 27% of individuals for whom we can link to parents and who report their high school, these estimates are noisier and potentially less representative than our baseline estimates. The Instagram data do not require parental linkage and capture all friends, not just high school friends, thereby producing a larger and more comprehensive sample. The limitation of the Instagram EC measure is that it measures EC among the 2005–2009 birth cohorts, rather than the 1978–1983 cohorts for which we measure economic mobility. However, the stability of both economic mobility^72^ and EC (Supplementary Fig. 1) within a location over time mitigates the consequences of this misalignment.

### Measuring cohesiveness

We represent a set of friendships by the matrix **A** ∈ {0, 1}^*n*×*n*^, where  *A*_*ij*_ = 1 denotes the existence of a friendship (edge) between individuals *i* and *j*, and *A*_*ij*_ = 0 denotes the absence of a friendship. We focus on three measures of the structure of **A**: clustering and support ratio, which are measures of local correlation in friendships, and spectral homophily, a measure of overall network fragmentation. Other measures of cohesiveness, such as algebraic connectivity^88^, are also informative, but are difficult to compute or even approximate for networks of the scale we analyse. The three measures of cohesiveness we focus on here have the advantage of being computationally tractable in large samples.

#### Clustering

Previous work^33^ has argued that if person *i* is friends with both persons  *j* and *k*, then having  *j* and *k* be friends with each other can help them collectively pressure and sanction person *i*, thereby helping to enforce norms. Motivated by this logic, many studies have measured the extent of such ‘network closure’ by the degree of clustering within a person’s network: the frequency with which two friends of that person are in turn friends with each other. Letting *N*_*i*_(**A**) denote the set of *i*’s friends and *d*_*i*_(*A*) its cardinality (the number of friends *i* has), the clustering of *i*’s network is defined as

4 $${{\rm{Clustering}}}_{i}({\bf{A}})=\sum _{k,j\in {N}_{i}({\bf{A}}),\,k < j}\frac{{A}_{kj}}{{d}_{i}({\bf{A}})({d}_{i}({\bf{A}})-1)/2}.$$

We measure clustering in a community *c* as the average of equation (4) across people living in that community as follows:

5 $${{\rm{Clustering}}}_{c}=\frac{{\sum }_{i\in c}{{\rm{Clustering}}}_{i}({\bf{A}})}{{N}_{c}}.$$

#### Support ratio

Letting **A**^*c*^ denote the subset of friendships between individuals who are both members of community *c*, we measure a community *c*’s support ratio as the overall frequency with which pairs of friends have at least one friend in common, focusing only on the people and friendships within that community:

6 $${{\rm{S}}{\rm{u}}{\rm{p}}{\rm{p}}{\rm{o}}{\rm{r}}{\rm{t}}{\rm{r}}{\rm{a}}{\rm{t}}{\rm{i}}{\rm{o}}}_{c}=\frac{|\{(ij):i,j\in c,{A}_{ij}^{c}=1,{[{({A}^{c})}^{2}]}_{ij} > 0\}|}{|\{(ij):i,j\in c,{A}_{ij}^{c}=1\}|}.$$

#### Spectral homophily

Spectral homophily measures the extent to which a network is fragmented into separate groups, and relates to the speed of information aggregation in a network. A wide variety of algorithms can detect subcommunities^89^, and spectral homophily provides a simple measure of how strongly a network splits into such subcommunities. Formally, spectral homophily is the second largest eigenvalue of the degree-normalized (row-stochasticized) adjacency matrix ${{{\bf{A}}}^{c}}_{{\bf{s}}}\in {[0,1]}^{n\times n}$. We measure spectral homophily in each county on the basis of the set of friendships among individuals in our primary sample living in that county. Friendship matrices are too sparse to estimate spectral homophily reliably at the ZIP code level. In the rare instances when there are fully isolated nodes within a county, we calculate spectral homophily on the largest connected component, which usually makes up the majority of users living in a county.

### Measuring civic engagement

#### Volunteering rate

We start with the set of all Facebook Groups in the United States that are predicted to be about volunteering or activism based on their titles and do not have the privacy setting ‘secret’ enabled. To further improve this classification, we manually review the 50 largest such groups in the United States and the largest such group in each state, and remove the very small number of groups that are clearly misclassified. We then define the volunteering rate as the share of Facebook users in an area who are a member of at least one volunteering or activism group.

#### Civic organizations

We start with the set of all Facebook Pages in the United States that are categorized as ‘public good’ pages on the basis of the page title and page category. We then remove pages that do not have a website linked, do not have a description on their Facebook page or do not have an address listed. We then assign the page to a ZIP code and county on the basis of its listed address, and calculate the density of civic organizations as the number of such pages per 1,000 Facebook users in the area.

### Correlations

We weight all correlations and regressions by the number of individuals with below-national-median parental income as calculated using Census data^72^, unless otherwise noted. We cluster standard errors in all county-level regressions by commuting zone and ZIP-code-level regressions by county to adjust for potential spatial autocorrelation in errors, unless otherwise noted.

The causal effect estimates used in the ‘Causal effects of place versus selection’ section are identified solely from individuals who move across areas and are therefore much less precise than the baseline observational estimates of economic mobility used in the rest of the paper, making it necessary to adjust for attenuation bias in those correlation estimates due to sampling error. We adjust for attenuation bias by dividing the raw correlation between the causal estimates of mobility and EC by the square root of the reliability of the causal estimates of mobility, as estimated by Chetty and Hendren^76^. The causal effect estimates are also unavailable at the ZIP-code level owing to small sample sizes for ZIP-code-level moves. This is why we focus on the observational estimates of upward income mobility in our baseline analysis.

### Privacy and ethics

This project focuses on drawing high-level insights about communities and groups of people, rather than individuals. We used a server-side analysis script that was designed to automatically process the raw data, strip the data of personal identifiers, and generate aggregated results, which we analyzed to produce the conclusions in this paper. The script then promptly deleted the raw data generated for this project. While we used various publicly available sources of aggregate statistics to supplement our analysis, we do not link any external individual-level information to the Facebook data. All inferences made as part of this research were created and used solely for the purpose of this research and were not used by Meta for any other purpose.

A publicly available dataset, which only includes aggregate statistics on social capital, is available at https://www.socialcapital.org. We use methods from the differential privacy literature to add noise to these aggregate statistics to protect privacy while maintaining a high level of statistical reliability; see https://www.socialcapital.org for further details on these procedures. The project was approved under Harvard University IRB 17-1692.

### Reporting summary

Further information on research design is available in the Nature Research Reporting Summary linked to this paper.

## Online content

Any methods, additional references, Nature Research reporting summaries, source data, extended data, supplementary information, acknowledgements, peer review information; details of author contributions and competing interests; and statements of data and code availability are available at 10.1038/s41586-022-04996-4.

### Supplementary information

Supplementary Information This file contains information on data and sample construction, supplementary methods and supplementary discussion.

Reporting Summary

## Figures and Tables

**Extended Data Table 1:**
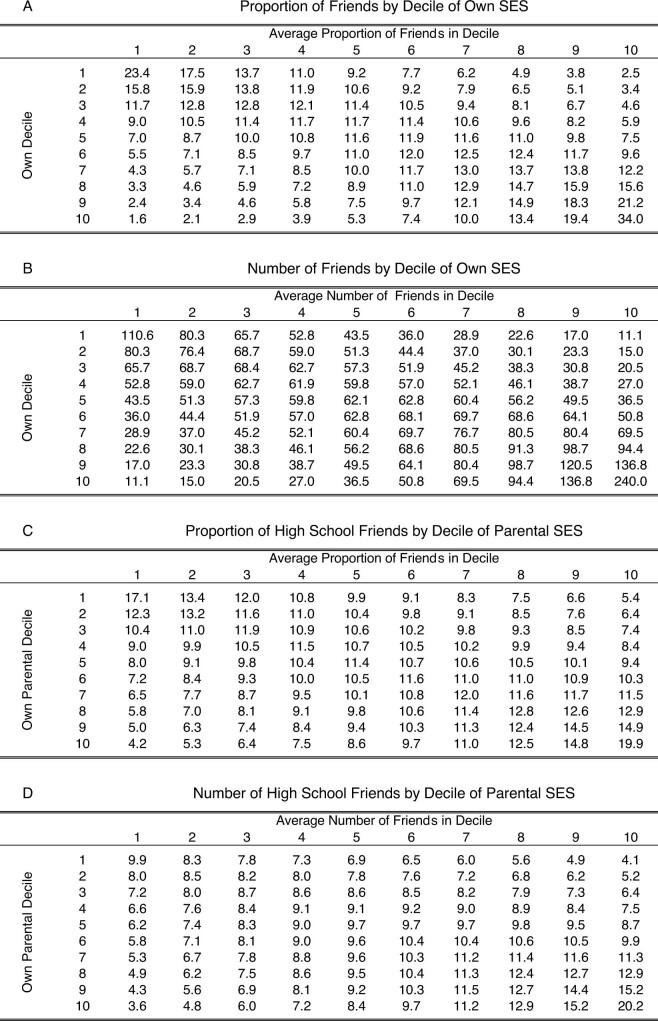
Conditional Distributions of Friends’ SES by Own SES Decile

This table shows the number and percentage of friends from each SES decile by individuals’ own SES deciles. Panels A and B, respectively, calculate friend shares and average numbers of friends using current SES and individuals’ entire friendship networks. Panels C and D calculate friend shares and average numbers of friends using parental SES and individuals’ high school friendship networks—the set of peers within three birth cohorts who attended the same high school. In Panels A and B, the statistics are calculated on our primary analysis sample (see Extended Data Table 4a). In Panels C and D, they are calculated on the subsample with linked parental SES and high schools (see Extended Data Table 4b). See Supplementary Figure 16 for a heat map of an analogous matrix showing friendship links by percentile (rather than decile) of own SES.

**Extended Data Table 2:**
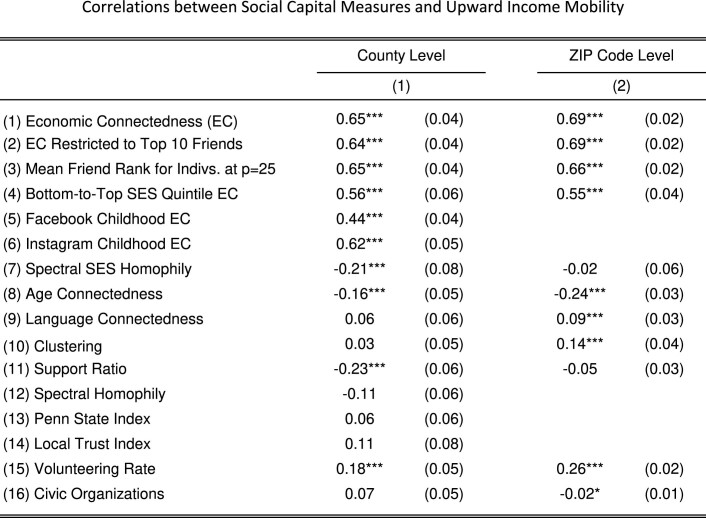
Correlations between Social Capital Measures and Upward Income Mobility

This table reports county-level and ZIP-code-level univariate correlations between upward income mobility and the full set of social capital measures we construct in this paper, expanding upon the subset shown in Fig. 3a. All correlations are weighted by the number of children born to parents with below-median income as reported in the Opportunity Atlas^72^. Standard errors (reported in parentheses) are clustered by commuting zone for county-level correlations and by county for ZIP code-level correlations. For definitions of the variables used in this table, see the notes to Fig. 3; the Economic Connectedness, Cohesiveness, and Civic Engagement sections of Main Text and Methods; and Supplementary Information A.5.3 and B.2. Asterisks indicate the level of significance: *5%, **1%, ***0.1%.

**Extended Data Table 3:**
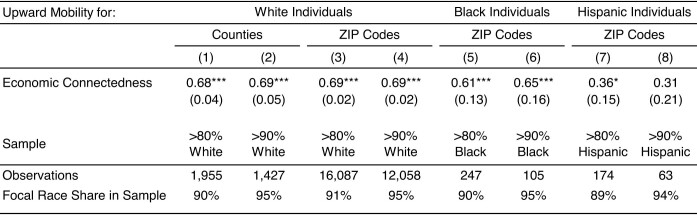
Associations between Race-Specific Upward Income Mobility and Economic Connectedness in Racially Homogeneous Areas

This table presents correlations between race-specific measures of upward income mobility and economic connectedness across racially homogeneous counties and ZIP codes. Data on upward income mobility by race are obtained from Chetty et al.^73^. Upward mobility is measured as the predicted household income rank in adulthood for children in the 1978-83 birth cohorts with parents at the 25th percentile of the national income distribution. In columns 1-4, we correlate upward mobility for white individuals with economic connectedness in counties and ZIP codes where over 80% or over 90% of the population is white (based on data from the 2000 Census). In columns 5-6, we correlate upward mobility for Black individuals with economic connectedness in ZIP codes where over 80% or over 90% of the population is Black. In columns 7-8, we correlate upward mobility for Hispanic individuals with economic connectedness in ZIP codes where over 80% or over 90% of the population is Hispanic. Because the statistics on upward mobility are constructed using individuals who grew up in the US. and a large share of Hispanic individuals are immigrants, in Columns 7-8 we measure economic connectedness using only individuals who have a US hometown in the Facebook data. The bottom row of the table shows the percentage of individuals in the estimation sample of the focal racial group (e.g., percentage white in columns 1-4). For white and Black individuals, the correlations are weighted by number of children with below-median parental income as calculated in the Opportunity Atlas^72^ using Census data. In columns 7-8, the weights are the number of children who have a US hometown and have below-median parental SES in the Facebook data. Standard errors (reported in parentheses) below each correlation are clustered at the commuting zone level for county-level correlations and at the county level for ZIP code-level correlations. Asterisks indicate the level of significance: *5%, **1%, ***0.1%.

**Extended Data Table 4:**
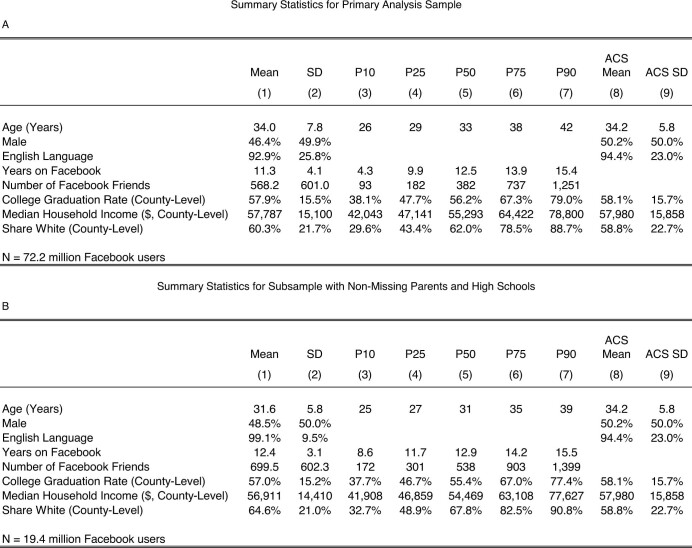
Summary Statistics for Analysis Samples

Panel A presents summary statistics for our primary analysis sample, which consists of individuals between the ages of 25 and 44 as of May 28, 2022 who reside in the United States, have been active on the Facebook platform at least once in the previous 30 days, have at least 100 US-based Facebook friends, and have a non-missing residential ZIP code. Panel B replicates Panel A for the sample used to measure childhood economic connectedness—users whom we can link to parents with valid SES predictions (see Supplementary Information A.2) and assign a high school (see Supplementary Information A.3). For each variable, columns 1-7 present means, standard deviations, and selected percentiles. Columns 8 and 9 report means and standard deviations for the corresponding variables using the nationally representative 2012-2016 American Community Survey (ACS) for median household income and the 2014-2018 ACS for the remaining variables. Age and gender are self-reported by individuals. *English Language* refers to whether users set their language to English in the Facebook data and to the share of individuals who speak English only or speak English “very well” in the ACS data. *Years on Facebook* and *Number of Facebook Friends* are only observed in the Facebook data. In both panels, we report the number of Facebook friends within our primary analysis sample, as opposed to the total number of Facebook friends. The remaining variables are obtained from the 2014-2018 American Community Survey (ACS) and are assigned based on an individual’s residential county. The ACS means for these variables are population-weighted averages of the same county-level variables.

## Data Availability

The only data shared outside of Meta were aggregate statistics on social capital (by county and ZIP code, etc.). We used methods from the differential privacy literature to add noise to these aggregate statistics to protect privacy while maintaining a high level of statistical reliability. See https://www.socialcapital.org for further details on these procedures.

## Code availability

The code that supports the findings of this study using the publicly released data is available at https://opportunityinsights.org.
