# Supplementary material for: Social capital I: measurement and associations with economic mobility
Source: Nature. 2022 Aug 1;608(7921):108–21. doi: 10.1038/s41586-022-04996-4 (PMC9352590; doi:10.1038/s41586-022-04996-4)
Supplement: Supplementary file 1 — This file contains information on data and sample construction, supplementary methods and supplementary discussion. [file 41586_2022_4996_MOESM1_ESM.pdf]

nature portfolio

https://doi.org/10.1038/s41586-022-04996-4

Supplementary information

Social capital I: measurement and associations with economic mobility

In the format provided by the authors and unedited

Supplementary Information For "Social Capital I: Measurement and Associations with Economic Mobility"

Raj Chetty*, Matthew O. Jackson*, Theresa Kuchler*, Johannes Stroebel*,
Nathaniel Hendren, Robert B. Fluegge, Sara Gong, Federico Gonzalez, Armelle Grondin,
Matthew Jacob, Drew Johnston, Martin Koenen, Eduardo Laguna-Muggenburg,
Florian Mudekereza, Tom Rutter, Nicolaj Thor, Wilbur Townsend, Ruby Zhang,
Mike Bailey, Pablo Barberá, Monica Bhole, and Nils Wernerfelt

Contents

A Supplementary Information on Data and Sample Construction

A.1 Sample Coverage
A.2 Linking Individuals to Parents
A.3 Identifying High Schools
A.4 Instagram Data
A.5 External Data
  A.5.1 Neighborhood Characteristics
  A.5.2 Add Health Survey Data
  A.5.3 External Social Capital Measures

B Supplementary Methods

B.1 Measuring Socioeconomic Status
B.2 Other Social Capital Measures
B.3 Standard Errors of Economic Connectedness Measures

C Supplementary Discussion

C.1 Prior Work with Facebook Data
C.2 Associations Between Civic Engagement and Economic Mobility
C.3 Associations Between Social Capital and Life Expectancy
C.4 Friendship Rates by SES Percentile Rank

---

* R.C., M.J., T.K., and J.S. contributed equally to this work.

A Supplementary Information on Data and Sample Construction

In this section, we provide further details on how we construct the data we use for our analysis, expanding on the discussion in the Methods section of the main paper, Chetty et al. (2022a).

A.1 Sample Coverage

Since much of our analysis relies on variation across areas, it is important that our sample has good coverage not just nationally but also across locations. Supplementary Figure 12 shows the geographic distribution of relative Facebook coverage rates, defined as the total number of Facebook users in our sample divided by population counts for that age group based on the 2014-2018 ACS, normalized by the national average coverage rate. Rates of coverage are high across the country, although usage rates are slightly lower in California. The correlations between sample sizes in the Facebook data and population counts are 0.99 across counties and 0.91 across ZIP codes.

Perhaps most important for our analysis, Facebook coverage does not vary systematically across locations with different income levels or demographic characteristics. Median household incomes, racial shares, and levels of education (based on ACS data) by county of residence are similar for Facebook users and the nationally representative ACS sample, as shown in Extended Data Table 4. For example, median county incomes for individuals in our sample differ from those for the ACS by $193 on average, a small difference relative to the standard deviation of median incomes across counties of more than $15,000. Supplementary Figure 13 confirms that our coverage is consistent across the full distribution of ZIP code incomes.

To evaluate whether our main findings might be biased by differences in rates of Facebook coverage across areas, we replicate the key results in Figures 3 and 5 of Chetty et al. (2022a), restricting the sample to the top 25% of counties in terms of Facebook coverage. We find similar results in this subsample of counties (Supplementary Figure 14).

A.2 Linking Individuals to Parents

To construct measures of parental SES, we link individuals in our primary analysis sample (ages 25 to 44) to their parents. To do so, we start from the subsample of individuals in our primary analysis sample who self-report their parents. Since many individuals do not report their parents, we then use three methods sequentially to impute linkages for individuals who do not self-report parents: public user-generated wall posts that provide some indication of parental relationships; matching based on age and last names (using hashed strings to protect confidentiality);[1] and familial relationships (e.g., inferring parents based on information provided by self-reported siblings).

We evaluate the accuracy of the imputed parental links by computing the false positive rate in the subsample of individuals who self-report their parents. For such individuals, we assign mothers and fathers using the self-reports and imputation procedures separately. The match rates between the self-reports and the imputed linkages are 78% and 83% for mothers and fathers, respectively. The correlations of parental SES between the self-reported and imputed parental matches are 0.87 and 0.91 for mothers and fathers, respectively. Out of all final parent-child linkages, 39.4% are based on self-reports and the rest are based on imputations.

Overall, we match 46% of the individuals in our primary sample to parents. 31% of the primary sample is assigned a parental SES. We are unable to assign an SES to all parents because some of them are above age 65 in 2022 and we do not assign SES to those over age 65 since we cannot measure SES reliably for retired individuals.

[1] Hashing is a procedure that recodes a specific string into a unique numerical identifier. This enables us to perform name-matching across users while only using their corresponding numerical identifiers for privacy protection.

A.3 Identifying High Schools

We assign individuals to high schools based on self-reported high schools, self-reported hometowns, and information from their social networks.

We begin by matching self-reported high school names to the National Center for Education Statistics' (NCES) comprehensive surveys of U.S. public and private schools. We drop virtual schools, schools located in the five U.S. territories or on military bases abroad, and schools with fewer than 50 students. For individuals who self-report a common high school name (e.g., "Central High School"), we only include that self-report if the individual also reports a hometown that matches the school's location. We also exclude self-reported schools where users have fewer than 10 friends (with ages within three years of their own). For the 3.3% of individuals with multiple self-reported high schools, we assign the school at which the individual has the greatest number of friends whose ages are within three years of the individual's own age.

For people without a validated self-reported high school, we use their friendship network to impute their high school. For this imputation, we only consider friends who have a valid self-reported high school and who are within three years of the individual's age. We then calculate the ratio of an individual's friends in the high school where they have the most friends relative to the schools where they have the next most friends, and assign the user to the first high school if this ratio exceeds two (we further require that the individual has at least five friends in the first high school). We evaluate the accuracy of this imputation approach using the sample of users with validated self-reports. For users with a valid self-reported high school, the network-imputed high school matches the self-reported high school 97.4% of the time.

Using this algorithm, we observe high schools for 74.9% of individuals in our analysis sample; 53.8% are assigned via self-reports and 21.1% via imputation based on their friendship network.

A.4 Instagram Data

We construct the Instagram sample used to measure childhood EC by restricting to personal users (not business pages) in the United States who had not deactivated their account, had been active on the platform within the last 30 days, and were predicted to be between 13 and 17 years of age as of May 28, 2022. We obtain this age range prediction from a Meta-internal machine learning model, which is trained on multiple signals including Instagram accounts linked to Facebook accounts (see `https://about.fb.com/news/2021/07/age-verification/` for details on this model). We remove influencers and prominent public figures from the sample, defined as accounts that are above the 99th percentile in terms of followers or that receive more than 80,000 messages per week. Lastly, we remove accounts that do not have at least 5 reciprocal follows; that is, we require that at least five accounts a user follows must follow them back.

Supplementary Figure 15 plots the mean SES percentile rank of individuals' friends against their own SES percentile rank, replicating Figure 1 in the Instagram sample. We find slightly greater homophily in the Instagram sample than in our baseline analysis sample, with an SES rank-rank slope of 0.51 (and a slope of 0.47 between the 10th and 90th percentiles of own SES).

A.5 External Data

In this section, we describe the external (non-Facebook) data we use in our analysis. Note that we do not link any external individual-level information to the Facebook data.

A.5.1 Neighborhood Characteristics

American Community Survey. We obtain data on median incomes by Census block group and ZIP code from the 2014–2018 American Community Survey (ACS). These block-group-level income data are used in our machine learning algorithm for predicting socioeconomic status (see Supplementary Information B.1).

We also use the ACS to construct measures of racial and income segregation across tracts within each county. We measure racial segregation using Theil's H index, following equation (4) in Chetty et al. (2014). We compute the racial segregation index based on the shares of four groups in each tract: whites, Blacks, Hispanics, and all others. We measure income segregation using the generalized H index averaging across all income percentiles introduced by Reardon and Bischoff (2011), following equation (5) from Chetty et al. (2014). We are unable to construct reliable estimates of the segregation variables for counties with populations below 20,000 and cannot construct estimates for counties that consist of a single tract. We therefore omit those counties when analyzing income and racial segregation.

Opportunity Atlas. Data on economic mobility by Census tract and county are obtained from the publicly available Opportunity Atlas (Chetty et al. 2018). We define upward income mobility in each area as the average income percentile in adulthood of a child born to parents at the 25th percentile of the income distribution. We aggregate the Census tract data on upward mobility to the ZIP code (ZCTA) level using the number of children with below-median parental income as weights.

We also use the following variables from the Opportunity Atlas and Chetty et al. (2014), which are derived from the ACS and other sources, for correlational analyses: jobs within 5 miles, job growth rate 2004–2013, employment rate in 2000, Gini coefficient, top 1% share, share above poverty line, mean household income, mean 3rd grade math score, share college graduates, Black share, Hispanic share, and single parent share. We measure income inequality in each county as the raw Gini coefficient estimated using tax data minus the income share of the top 1% to obtain a measure of inequality among the bottom 99%, which Chetty et al. (2014) show is most predictive of differences in upward mobility across areas. We exclude four small counties where the resulting estimate of the Gini coefficient is negative.

A.5.2 Add Health Survey Data

The National Longitudinal Study of Adolescent to Adult Health (Add Health) is a nationally representative sample of students who attended grades 7–12 in 1994–1995. We use data from the first wave of Add Health (1995), which contains information on students' self-reported friendship networks as well as household income reported by the female head of household (unless there is no such individual in the household). The sample consists of 18,924 students, 90 percent of whom have information regarding parental household income. The social network data in the Add Health survey was constructed by asking the teenagers to nominate at most 5 male and 5 female friends. We define an individual's set of friends as those they nominate as one of their friends as well as all individuals who list them as one of their friends. Figure 1b is generated by ranking students according to their household income and averaging friends' household income ranks for each student.

A.5.3 External Social Capital Measures

Penn State Index. We use the county-level social capital index constructed by Rupasingha et al. (2006) as an index of civic engagement. This index is the first principal component of a set of four variables: the Census response rate; voter turnout in presidential elections; the number of per capita non-profit organizations; and an aggregate of a set of variables containing the number of various organizations (such as religious, civic, and labor organizations).

Local Trust Index. We measure local trust using the state-level social support subindex from Social Capital Project (2018). It comprises four indicators derived from survey data: the share of adults who "get the social and emotional support [they] need"; the share of adults who do favors for neighbors at least once a month; the share who trust most or all of their neighbors; and the average number of "close" friends reported.

B Supplementary Methods

This section provides further details on three aspects of our methods: (1) our algorithm for measuring socioeconomic status; (2) the construction of certain social capital measures we analyze in Supplementary Tables; and (3) estimation of standard errors for our publicly available estimates of economic connectedness.

B.1 Measuring Socioeconomic Status

We construct our baseline measure of socioeconomic status by combining various proxies for SES (e.g., median incomes in one's residential ZIP code, cell phone model, college attended, etc.) that are observed in the Facebook data. We estimate SES solely for the purpose of this research project, and delete these SES measures at the end of our analysis; the SES measure we use is not an internal measure provided by Facebook.

The underlying proxies we use for SES, which are listed in Supplementary Table 4, can be combined in many ways to create a single SES measure. We seek to identify the combination of proxies that best predicts household median income at the block group level using a machine learning model. To identify this combination, we begin by forming a training sample that consists of users aged 25 to 64 in our primary analysis sample with Location History (LH) enabled. We observe the residential Census block group of LH users, and use this information to assign each of them the median household income in their residential block group using data from the publicly available 2014–2018 American Community Survey (ACS), separately for the 25–44 and 45–64 year age buckets.

Next, we train a gradient-boosted regression tree to predict the log of block group median income in the LH sample, using the SES proxies described in Supplementary Table 4 as predictors. Because these predictors are available for non-LH users as well, this model allows us to create SES predictions for all users in our analysis sample. To reduce the risk of overfitting, we impose a maximum tree depth and hold out 10% of our data as a validation sample. Reassuringly, we find that the model has similar performance in the validation sample.

After estimating the model, we construct predicted values of SES for the entire sample (including non-LH users). We then convert the individual-level SES predictions obtained from the model to local rankings of individuals within each county. We map these local rankings to ranks in the national SES distribution by using data on income distributions by county from the ACS, which releases the number of households in 16 income bins in each county. For each county, we fit a parametric distribution to this discrete data, using Stata's mgbe (multimodel generalized beta estimator) command, as described in von Hippel et al. (2015). The distribution is selected using maximum likelihood estimation with the following log-likelihood function:

$$l(\theta|X) = \ln \prod_{b=1}^{B} (P(l_b < X < u_b))^{n_b} = \ln \prod_{b=1}^{B} (F(u_b) - F(l_b))^{n_b}, \tag{1}$$

where for each income bin $b \in \{1, ..., B = 16\}$, the lower and upper boundaries are denoted by l_b and u_b, and the number of observations in the bin by n_b. We estimate the parameters, θ, for the cumulative distribution function, $F(.)$, that maximize the likelihood of each observation falling in its respective income bin (i.e. $P(l_b < X < u_b)$). We perform a similar exercise for each of the seven distributions in the generalized beta family (dagum, singh-maddala, beta, loglogistic, gamma, generalized gamma, and weibull), and choose the distribution with the smallest Akaike information criterion (AIC). Using the estimated local income distribution, we translate the local rankings in each county obtained from the machine learning model to estimates of levels of income. Lastly, we rank all individuals in the national distribution relative to others in their birth cohort to obtain our final SES ranks.

For sensitivity analyses, we also construct three other measures of socioeconomic status:

ACS Block Group Median Income. We assign each user in the LH sample the household median income for their residential block group as described above.

ACS ZIP Code Household Median Income. To construct an area-level proxy that is available for the entire primary analysis sample, we assign each user the household median income for their ZIP code based on the 2014–18 ACS. ZIP code is available for all users, not just those in the LH sample.

Z-Score Index. We create a different combination of the SES proxies that does not rely on the machine learning model by taking a mean of the z-scores of the following six variables: the ACS ZIP code median household income, days since joining Facebook, phone price, college tier, web usage, and an indicator for self-reported graduate school. We focus on this set of variables because they are most predictive of block-group SES in the ML model and have no missing observations. We construct the z-score for each variable by subtracting the mean and dividing by the standard deviation, separately by birth cohort. The composite index is then constructed as the unweighted average of the z-scores, with each variable signed so that higher values correlate with higher SES predictions from the machine learning model.

As above, we create SES ranks based on all of these measures by ranking individuals relative to all others in the primary sample in their birth cohort.

Supplementary Table 5 shows how measures of economic connectedness based on each of these SES proxies correlate with each other and with economic mobility across counties. All the measures are highly correlated with each other and with upward income mobility, indicating that our results are insensitive to the specific algorithm used to measure SES.

B.2 Other Social Capital Measures

In this section, we describe other social capital measures that we analyze in the Supplementary Information but do not discuss in the main text.

Mean Friend Rank for Individuals at p25. To construct this measure, we first calculate the mean SES rank of each individual's friends. In each area (county or ZIP), we then regress this mean friend rank on the individual's own rank and calculate the predicted average friend rank from this regression for individuals at the 25th percentile of the national SES distribution. The difference between this measure and our baseline measure of economic connectedness is that it controls for differences in the SES distribution among individuals with below-median SES across areas.

Bottom-to-Top SES Quintile EC. Analogous to our baseline economic connectedness measure, this statistic is five times the share of top-SES-quintile friends among bottom-SES-quintile individuals.

Spectral SES Homophily. This is a summary measure of the degree of connection across individuals in different quintiles of the national SES distribution in a given area c, as defined in Golub and Jackson (2012). We begin by constructing a five-by-five matrix whose i, j elements are the share of friends from quintile j among individuals in quintile i of the national SES distribution. We then define spectral SES homophily as the second eigenvalue in magnitude of this matrix, which ranges from 0 to 1, with higher values corresponding to more homophily by SES. If the matrix had 0.2 in every entry, then each quintile would have 1/5 of its friendships in every quintile, and there would be no homophily. The second eigenvalue of such a matrix is 0—corresponding to 0 homophily by SES. If the community were fully homophilistic and had no connections across SES quintiles, then the matrix would be the identity matrix and would have a second eigenvalue of 1.

B.3 Standard Errors of Economic Connectedness Estimates

This section explains how we construct standard errors for our estimates of economic connectedness for each county and ZIP code in our sample (as well as for high schools and colleges in our companion paper, Chetty et al. 2022b).

Recall that we define EC in a given area c as the mean level of individual economic connectedness (IEC) of low-SES (below-median) users:

$$\text{EC}_c = \frac{\sum_{i \in L \cap c} \text{IEC}_i}{N_{Lc}}, \tag{2}$$

where N_{Lc} is the number of low-SES users in c.

Since this is a mean of individual-level values, a natural estimator for the standard error of the EC estimate that ignores the network structure of the data is:

$$\text{se}_{\text{naive}}(\text{EC}_c) = \sqrt{\frac{\sum_{i \in L \cap c} (\text{IEC}_i - \text{EC}_c)^2}{N_{Lc} - 1}} \tag{3}$$

However, this "naive" standard error estimate—which assumes that IEC_i is independent and identically distributed across users within c—is likely to be an underestimate of the true standard error of our EC estimate because IEC is likely to be correlated across people in a given community c. Perhaps the most important source of such correlation is that all users in a given community draw their friends from the same pool of potential friends (e.g., the set of students who attend a given school), which is itself stochastic given the limited size of each group.

We correct for this additional source of variance in our estimates using the bootstrap-based approach described in Section 2.3 of Davezies et al. (2021). Each iteration of the bootstrap yields a potential network that could have been observed, and hence a corresponding value of EC that could have been observed. We calculate the standard error of EC in a given cell c as the standard deviation of EC across these potential realizations. Each iteration of the bootstrap consists of the following steps:

1. Assign each individual i in the analysis sample a weight π_i from a Poisson(1) distribution, reflecting the number of times the user is "sampled" in this bootstrap iteration.

2. Construct a sampled friend list from friendships in the original Facebook graph. For two users i and j with Poisson weights π_i and π_j who are friends, their friendship appears $\pi_i \times \pi_j$ times in the sampled friend list. Note that if $\pi_j = 0$, this friendship will not appear in the sampled friend list.

3. Calculate a new IEC for each individual i using this sampled friend list. For a given individual i we calculate their new IEC as two times the weighted (using weights π_j from step 1, where j indexes friends of i) proportion of their friends with high SES.

4. Take the weighted average of IEC over individuals i in the cell using the weights π_i from step 1.

The average standard error of EC in each setting using this bootstrap method is similar to an estimate of the standard deviation of the noise component of EC across groups within each setting based on split-sample estimates of the reliability of the EC estimates. We estimate reliability by randomly splitting the nodes in each group into two and then correlate EC estimates in the two split samples with each other. We then use this reliability estimate to calculate the portion of the total variance in EC that is due to noise within each setting.

Note that there may be other sources of correlation between the IECs of individuals in a given cell c that are not fully captured by the bootstrap method or split sample reliability calculation, so the standard error estimates we report should be interpreted with caution.

C Supplementary Discussion

C.1 Prior Work with Facebook Data

Facebook data have been used to study the effects of social networks on a variety of outcomes in prior work: patent citations (Bailey et al. 2018a), home purchasing decisions (Bailey et al. 2018b), mortgage choices (Bailey et al. 2019a), cell phone adoption (Bailey et al. 2019b), labor market outcomes (Gee et al. 2017; Gee 2018), commuting flows (Bailey et al. 2020a), international trade flows (Bailey et al. 2021), investment decisions (Kuchler et al. 2020a), peer-to-peer lending (Allen et al. 2020), EITC claiming behavior (Wilson 2020), racial homophily (Wimmer and Lewis 2010), health behavior and beliefs (Bailey et al. 2020b), mortality rates (Hobbs et al. 2016), the spread of COVID-19 (Kuchler et al. 2020b), and the social integration of international migrants (Bailey et al. 2022). Although they use some of the same underlying data, prior studies have not constructed systematic measures of social capital or studied their determinants as we do here.

C.2 Associations Between Civic Engagement and Economic Mobility

Our results on the lack of a strong association between civic engagement and economic mobility may appear to be inconsistent with the findings of the Social Capital Project, who report stronger correlations between measures of what we term civic engagement and economic mobility (Social Capital Project 2018). The difference is explained primarily by the fact that we weight our correlations by the number of children with below-national-median parental income (which are very similar to population weights), whereas Social Capital Project reports unweighted correlations (Social Capital Project 2018). The unweighted state-level correlation between the Social Capital Project "social support" measure and our upward mobility measure is 0.50, whereas the weighted correlation is 0.17 (in contrast, weighted and unweighted correlations of economic connectedness with upward mobility are very similar). This is because civic engagement is more highly correlated with mobility (as well as connectedness) in rural areas. A further difference is that we measure economic mobility here as the mean adult income rank of individuals with low-income (25th percentile) parents, whereas the Social Capital Project focuses on a relative mobility measure—the difference between outcomes at the 25th and 75th percentile (Social Capital Project 2018). Economic connectedness remains one of the strongest predictors of relative mobility as well.

C.3 Associations Between Social Capital and Life Expectancy

We measure the association between social capital and life expectancy using publicly available data on life expectancy by county for individuals at the 25th percentile of the national income distribution from Chetty et al. (2016). Clustering coefficients and support ratios are much stronger predictors than economic connectedness of differences in life expectancy among low-income individuals across counties (Supplementary Figure 11). Perhaps surprisingly, higher levels of clustering and support are associated with *lower* life expectancy. One potential explanation for this correlation is that areas with relatively low life expectancy face challenges that create a demand for closely-knit communities to provide support.

C.4 Friendship Rates by SES Percentile Rank

In Supplementary Figure 16a, we plot friendship rates by own and friends' SES percentile ranks, showing the fraction of friends from each SES rank by an individual's own SES percentile rank. Individuals with similar SES are more likely to be friends with each other. Consistent with the higher slope in the upper tail of Figure 1, individuals in the upper tail of the SES distribution are especially likely to befriend those in the upper tail (Supplementary Figure 16b). 9% of the friends of individuals in the top 1% of the national SES distribution come from the top 1%; hence,

individuals in the top 1% are 9 times more likely to befriend those in the top 1% than would occur in the absence of homophily.

Supplementary References

Allen, Linda, Lin Peng, and Yu Shan (2020). "Social Networks and Credit Allocation on FinTech Lending Platforms". Working Paper.

Bailey, Michael, Rachel Cao, Theresa Kuchler, Johannes Stroebel, and Arlene Wong (2018a). "Social Connectedness: Measurement, Determinants, and Effects". *Journal of Economic Perspectives* 32.3, pp. 259–280.

Bailey, Michael, Ruiqing Cao, Theresa Kuchler, and Johannes Stroebel (2018b). "The Economic Effects of Social Networks: Evidence from the Housing Market". *Journal of Political Economy* 126.6, pp. 2224–2276.

Bailey, Michael, Eduardo Dávila, Theresa Kuchler, and Johannes Stroebel (2019a). "House Price Beliefs And Mortgage Leverage Choice". *The Review of Economic Studies* 86.6, pp. 2403–2452.

Bailey, Michael, Patrick Farrell, Theresa Kuchler, and Johannes Stroebel (2020a). "Social Connectedness in Urban Areas". *Journal of Urban Economics* 118, p. 103264.

Bailey, Michael, Abhinav Gupta, Sebastian Hillenbrand, Theresa Kuchler, Robert Richmond, and Johannes Stroebel (2021). "International Trade and Social Connectedness". *Journal of International Economics* 129, p. 103418.

Bailey, Michael, Drew Johnston, Martin Koenen, Theresa Kuchler, Dominic Russel, and Johannes Stroebel (2020b). *Social Networks Shape Beliefs and Behavior: Evidence from Social Distancing during the COVID-19 Pandemic.* Working Paper 28234. National Bureau of Economic Research.

Bailey, Michael, Drew M Johnston, Martin Koenen, Theresa Kuchler, Dominic Russel, and Johannes Stroebel (2022). *The Social Integration of International Migrants: Evidence from the Networks of Syrians in Germany.* Working Paper 29925. National Bureau of Economic Research.

Bailey, Michael, Drew M Johnston, Theresa Kuchler, Johannes Stroebel, and Arlene Wong (2019b). *Peer Effects in Product Adoption.* Working Paper 25843. National Bureau of Economic Research.

Chetty, Raj, John N. Friedman, Nathaniel Hendren, Maggie R Jones, and Sonya R Porter (2018). *The Opportunity Atlas: Mapping the Childhood Roots of Social Mobility.* Working Paper 25147. National Bureau of Economic Research.

Chetty, Raj, Nathaniel Hendren, Patrick Kline, and Emmanuel Saez (2014). "Where Is the Land of Opportunity? The Geography of Intergenerational Mobility in the United States". *The Quarterly Journal of Economics* 129.4, pp. 1553–1623.

Chetty, Raj, M Stepner, S Abraham, S Lin, B Scuderi, N Turner, A Bergeron, and D Cutler (2016). "The Association between Income and Life Expectancy in the United States, 2001-2014". *Journal of the American Medical Association* 315.16, pp. 1750–1766.

Chetty, Raj et al. (2022a). "Social Capital I: Measurement and Associations with Economic Mobility". *Nature.*

— (2022b). "Social Capital II: Determinants of Economic Connectedness". *Nature.*

Davezies, Laurent, Xavier D'Haultfœuille, and Yannick Guyonvarch (2021). "Empirical Process Results for Exchangeable Arrays". *The Annals of Statistics* 49.2, pp. 845–862.

Gee, Laura K. (2018). "The More You Know: Information Effects on Job Application Rates in a Large Field Experiment". *Management Science* 65.5, pp. 2077–2094.

Gee, Laura K., Jason Jones, and Moira Burke (2017). "Social Networks and Labor Markets: How Strong Ties Relate to Job Finding on Facebook's Social Network". *Journal of Labor Economics* 35.2, pp. 485–518.

Golub, Benjamin and Matthew O. Jackson (2012). "How Homophily Affects the Speed of Learning and Best-Response Dynamics". *Quarterly Journal of Economics* 127.3, pp. 1287–1338.

Hobbs, William R., Moira Burke, Nicholas A. Christakis, and James H. Fowler (2016). "Online social integration is associated with reduced mortality risk." *PNAS* 113.46, pp. 12980–12984.

Kuchler, Theresa, Yan Li, Lin Peng, Johannes Stroebel, and Dexin Zhou (2020a). *Social Proximity to Capital: Implications for Investors and Firms*. Working Paper 27299. National Bureau of Economic Research.

Kuchler, Theresa, Dominic Russel, and Johannes Stroebel (2020b). *The Geographic Spread of COVID-19 Correlates with Structure of Social Networks as Measured by Facebook*. Working Paper 26990. National Bureau of Economic Research.

Reardon, S and Kendra Bischoff (2011). "Income Inequality and Income Segregation". *American Journal of Sociology* 116.4, pp. 1092–1153.

Rupasingha, Anil, Stephan J Goetz, and David Freshwater (2006). "The Production of Social Capital in US Counties". *The Journal of Socio-Economics* 35.1, pp. 83–101.

Social Capital Project (2018). *The Geography of Social Capital in America*. Tech. rep. SCP Report Number 1-18.

von Hippel, Paul T., Samuel V. Scarpino, and Igor Holas (2015). "Robust Estimation of Inequality from Binned Incomes". *Sociological Methodology* 46, pp. 212–251.

Wilson, Riley (2020). "The Impact of Social Networks on EITC Claiming Behavior". *The Review of Economics and Statistics*, pp. 1–45.

Wimmer, Andreas and Kevin Lewis (2010). "Beyond and Below Racial Homophily: ERG Models of a Friendship Network Documented on Facebook." *American Journal of Sociology* 116.2, pp. 583–642.

SUPPLEMENTARY TABLE 1: Correlations of Social Capital Measures Across Counties

		(1)	(2)	(3)	(4)	(5)	(6)	(7)	(8)	(9)	(10)	(11)	(12)	(13)	(14)	(15)	(16)
Economic Connectedness	(1) Economic Connectedness (EC)	1.00															
	(2) EC Restricted to Top 10 Friends	0.99	1.00														
	(3) Mean Friend Rank for Indivs. at p=25	0.98	0.97	1.00													
	(4) Top/Bottom Quintile EC	0.74	0.75	0.81	1.00												
	(5) Childhood EC	0.61	0.62	0.57	0.39	1.00											
	(6) Instagram Childhood EC	0.82	0.82	0.80	0.60	0.56	1.00										
Connectedness on Other Dimensions	(7) Spectral SES Homophily	-0.24	-0.23	-0.21	-0.21	-0.02	-0.22	1.00									
	(8) Age Connectedness	-0.45	-0.44	-0.46	-0.40	-0.35	-0.31	-0.49	1.00								
	(9) Language Connectedness	0.10	0.08	0.07	-0.08	-0.12	0.11	-0.40	0.17	1.00							
Network Cohesiveness	(10) Clustering	0.01	0.01	-0.01	-0.05	-0.14	0.04	-0.55	0.51	0.38	1.00						
	(11) Support Ratio	-0.25	-0.27	-0.29	-0.39	-0.18	-0.23	-0.33	0.50	0.30	0.64	1.00					
	(12) Spectral Homophily	-0.09	-0.07	-0.05	0.08	0.12	-0.14	0.59	-0.49	-0.37	-0.61	-0.51	1.00				
Civic Engagement	(13) Penn State Index	0.31	0.29	0.30	0.12	0.11	0.22	-0.07	-0.04	0.08	0.39	0.28	-0.25	1.00			
	(14) Local Trust Index	0.35	0.33	0.28	0.01	0.09	0.31	-0.24	-0.04	0.25	0.34	0.32	-0.35	0.43	1.00		
	(15) Volunteering Rate	0.46	0.44	0.44	0.16	0.16	0.39	-0.21	-0.04	0.28	0.30	0.23	-0.35	0.44	0.43	1.00	
	(16) Civic Organizations	0.27	0.24	0.28	0.10	0.03	0.19	-0.15	0.05	0.16	0.37	0.23	-0.33	0.67	0.28	0.46	1.00

Notes: This table replicates Table 1 with the full set of social capital measures that we construct. See the Economic connectedness, Cohesiveness, and Civic engagement sections of Main Text, Supplementary Information A.5.3, and Supplementary Information B.2 for definitions of each of the measures in this table.

SUPPLEMENTARY TABLE 2: Heterogeneity in ZIP Code-Level Relationships between Social Capital and Upward Income Mobility across Counties

Variables	Mean Correlation	Signal SD of Correlations	Noise SD of Correlations	% Corrs Opp. Sign of Mean
	(1)	(2)	(3)	(4)
Economic Connectedness	0.68	0.19	0.12	0.0%
Clustering	0.39	0.34	0.15	12.5%
Support Ratio	-0.08	0.26	0.16	37.6%
Volunteering Rate	0.40	0.24	0.16	4.5%

Notes: This table presents statistics summarizing the variation across counties in the correlation between upward income mobility and various social capital measures (shown in the rows) across ZIP codes within the 250 most populous counties. For each county, we first estimate the correlation between upward income mobility and the social capital measure across ZIP codes within each county, weighted by the number of children born to parents with below-median income as calculated in the Opportunity Atlas (Chetty et al. 2018) using Census data. Column 1 reports the mean value of these correlations across counties. We then estimate the heteroskedasticity-robust standard error associated with the correlation in each county. We estimate the noise variance in the correlation coefficients across counties as the weighted mean of the squared standard errors of the correlation coefficients across counties. We calculate the signal component of the variance as the total (raw) variance of the correlation coefficients across counties minus the noise variance. The Signal SD of Correlations reported in column 2 is the square root of the signal variance and is an estimate of the latent variation in the underlying (signal) distribution of the correlations in the absence of noise due to sampling error. In column 3, Noise SD of Correlations denotes the square root of the noise variance in the correlations. Column 4 reports the estimated proportion of correlations with the opposite sign to the weighted mean of these correlations, using the signal standard deviation and assuming a Normal distribution.

SUPPLEMENTARY TABLE 3: Correlations Between Economic Connectedness and Racial Shares across Counties

	(1)	(2)	(3)	(4)	(5)
(1) Economic Connectedness (EC)	1.00				
(2) Share White	0.45	1.00			
(3) Share Black	-0.43	-0.47	1.00		
(4) Share Hispanic	-0.29	-0.74	-0.16	1.00	
(5) Share Asian	0.26	-0.44	-0.04	0.28	1.00

Notes: This table reports pairwise correlations between economic connectedness and racial shares across counties, weighted by number of children with below-median parental income as calculated in the Opportunity Atlas (Chetty et al. 2018) using Census data. Economic connectedness is twice the share of above-median-SES friends among below-median-SES people in our primary analysis sample. Racial shares are obtained from the 2000 Census.

SUPPLEMENTARY TABLE 4: Variables Used to Predict Socioeconomic Status in Machine Learning Model

Number	Feature	Description
1	Age	Current year minus year of birth
2	Age at registration	Age when user created Facebook account
3	City prediction	User's home city
4	College	College attended
5	Instagram	Indicator for whether the user has an Instagram account
6	County prediction	User's predicted county
7	Days since confirmed	Number of days since Facebook account creation
8	Gender	User gender
9	Graduate school	Self-reported indicator for attendance of graduate school
10	Language	User-specified language setting
11	Phone model	Model of primary phone used
12	Estimated phone price	Current price of primary phone used
13	Region (state) prediction	User's home state
14	ZIP prediction	User's home ZIP code
15	Relationship status	Indicators for current relationship status (e.g., married)
16	Usage L28	Number of days with activity on any Facebook platform in last 28 days
17	Mobile usage L28	Number of days with activity on Facebook mobile platform in last 28 days
18	Web usage L28	Number of days with activity on Facebook web platform in last 28 days
19	Mobile carrier	Carrier of user's mobile phone
20	Phone OS type	OS of user's phone (e.g., Android, iOS)
21	Avg donation amount	Average amount that user has donated on the platform
22	Time since last donation	Time since user's last donation on the platform

Notes: This table lists the set of variables that we use to predict individuals' socioeconomic status using the machine learning model described in Supplementary Information B.1.

SUPPLEMENTARY TABLE 5: Correlations between Economic Mobility and Measures of Economic Connectedness

	Upward Mobility (1)	Baseline ML Ests. (2)	ACS Block Group Median (3)	ACS Zip Code Median (4)	Z-Score (5)
Upward Mobility	1				
Baseline ML Estimates	0.646	1			
ACS Block Group Median	0.605	0.839	1		
ACS Zip Code Median	0.550	0.783	0.945	1	
Combined Z-Score Index	0.599	0.913	0.882	0.855	1

Notes: This table presents county-level correlations between upward income mobility and measures of economic connectedness constructed from various alternative proxies for SES described in Supplementary Information B.1. All correlations are weighted by the number of children born to parents with below-median income. Upward income mobility comes from Chetty et al. (2018), and is defined as the predicted household income rank in adulthood for children in the 1978-83 birth cohorts with parents at the 25th percentile of the national income distribution. The measures of SES we consider are: the baseline machine learning (ML) model prediction used in our main analysis; the median household income in a block group (available for the Location History subsample only) or ZIP code (available for the full primary sample) from the ACS; and a composite z-score index consisting of: (1) the number of days since account creation (an older account correlates with higher SES); (2) the price of the phone used by the individual; (3) the selectivity tier of college an individual attended; (4) the number of days out of the last 28 days that a user accessed Facebook using a web browser (more usage from a website correlates with higher SES); (5) an indicator for whether the user reports a graduate school (having a graduate school is associated with higher SES); and (6) the median household income in the user's ZIP code from the ACS. The composite index is constructed by standardizing each of these six variables (subtracting the mean, dividing by the standard deviation) and then taking an unweighted average of the six variables, with each variable signed so that higher values correlate with higher SES ML predictions.

SUPPLEMENTARY TABLE 6: Typology of Social Capital Measures

Type of Social Capital		Data Used to Construct Measures: Labeled Network	Unlabeled Network	Non-Network
	Cross-Type Connectedness (Bridging Capital)	Economic Connectedness<br>Age Connectedness<br>Language Connectedness<br>Spectral SES Homophily		Income Segregation<br>Racial Segregation
	Network Cohesiveness		Clustering<br>Support Ratio<br>Spectral Homophily	
	Civic Engagement			Volunteering Rate<br>Civic Organizations<br>Penn State Index<br>Local Trust Index

Notes: This table summarizes the various social capital measures we construct in the paper. The measures are divided into three types of social capital, shown in the three rows: cross-type connectedness (or bridging capital), network cohesiveness, and civic engagement. We then classify the measures by the type of data used to construct them in the three columns: network data with labels, unlabeled network data, and non-network data. The measures that we focus on in this paper are shown in black; the racial and income segregation measures, which use non-network data to measure connectedness between different types of groups, shown in grey, are analyzed in Table 2. This table covers only the measures we analyze in this paper and does not provide a comprehensive classification of many other measures of social capital discussed in prior work.

SUPPLEMENTARY FIGURE 1: Stability of County-Level Economic Connectedness Across Cohorts

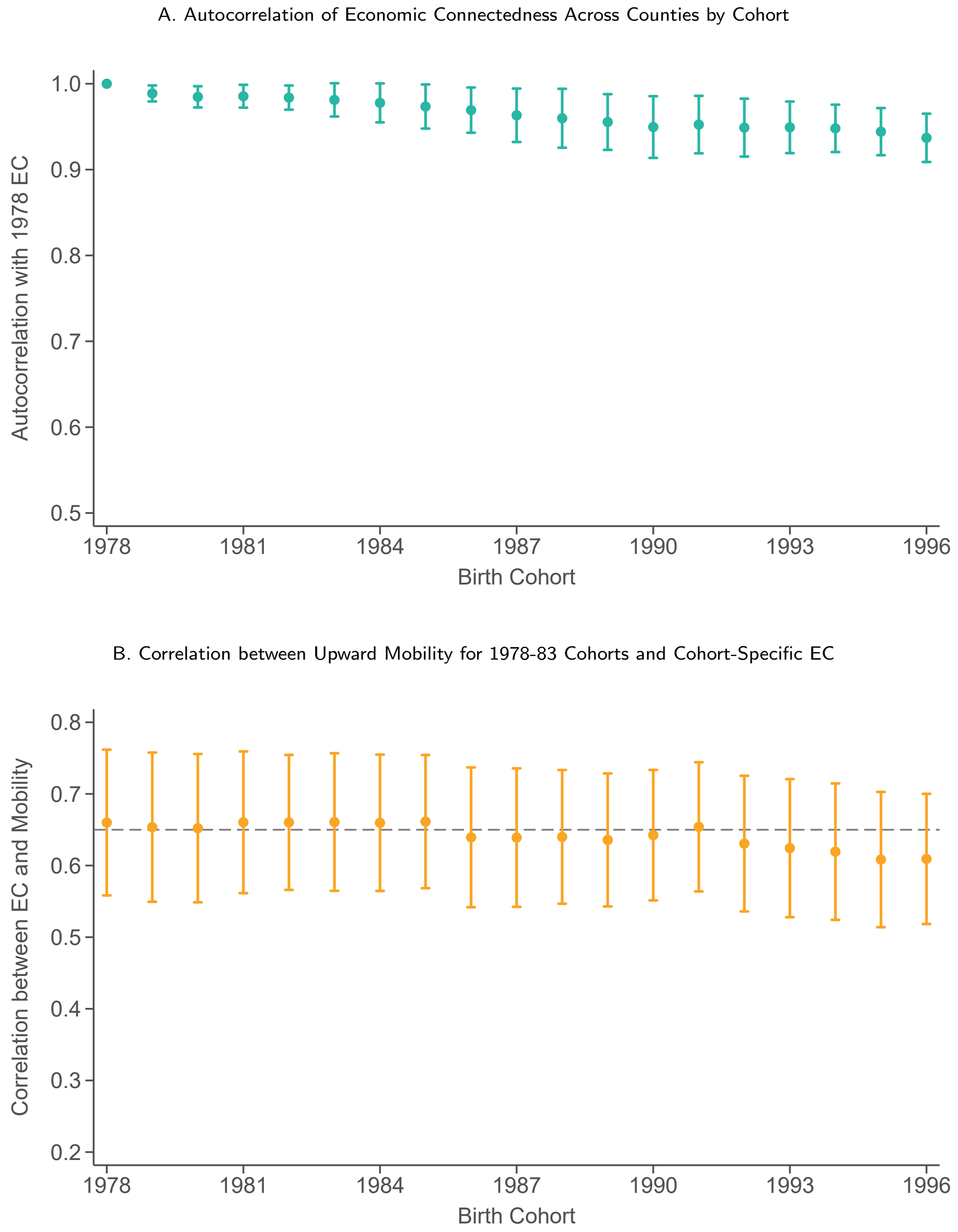

Notes: Panel A plots the correlation across counties between economic connectedness estimated using individuals in cohort x (for x ranging from 1978 to 1996) and EC estimated using individuals in the 1978 birth cohort. Panel B plots the correlation across counties between economic connectedness estimated using individuals in cohort x and upward income mobility for the 1978–83 birth cohorts, as estimated by Chetty et al. (2018). All correlations are weighted by the number of children in each county whose parents earn less than the national median income. Vertical lines represent 95% confidence intervals estimated using standard errors clustered by commuting zone. The dashed line in Panel B shows the correlation between economic connectedness estimated on the entire primary sample and upward income mobility.

SUPPLEMENTARY FIGURE 2: LASSO Estimates & Incremental R-Squared of Predictors in Multivariable Models

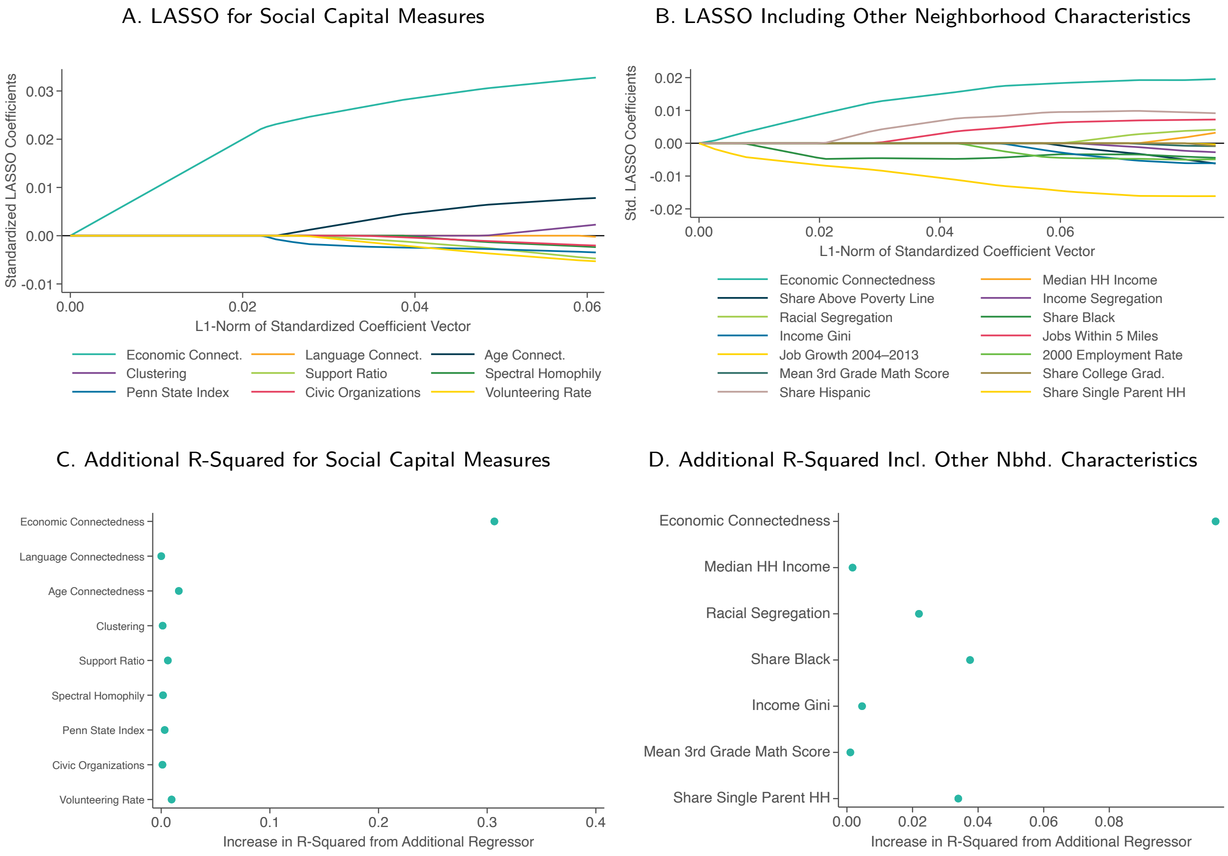

Notes: Panel A shows the standardized coefficients obtained from a LASSO regression of upward mobility on the set of social capital measures used in Figure 3a, plotted against the sum of the absolute values of the standardized coefficients. Panel B presents an analogous plot using economic connectedness and the other neighborhood characteristics used in Figure 5a. Panel C presents the incremental R-squared from adding each of the social capital measures to a regression that already includes all of the other measures used in the multivariable OLS regression specification in Figure 3b. Panel D presents an analogous plot for the regression specification in Figure 5b. All regressions are weighted by the number of children with parents who earn below the national median as reported in the Opportunity Atlas (Chetty et al. 2018) using Census data.

SUPPLEMENTARY FIGURE 3: ZIP Code-Level Correlations between Upward Mobility and Measures of Social Capital

A. Univariate Correlations

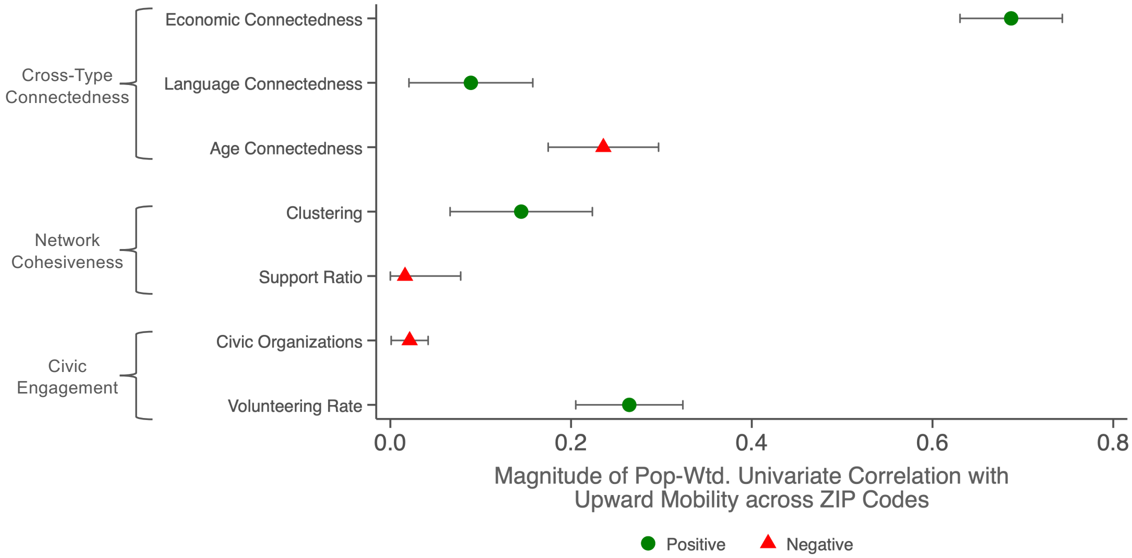

B. Coefficients from Multivariable Regression

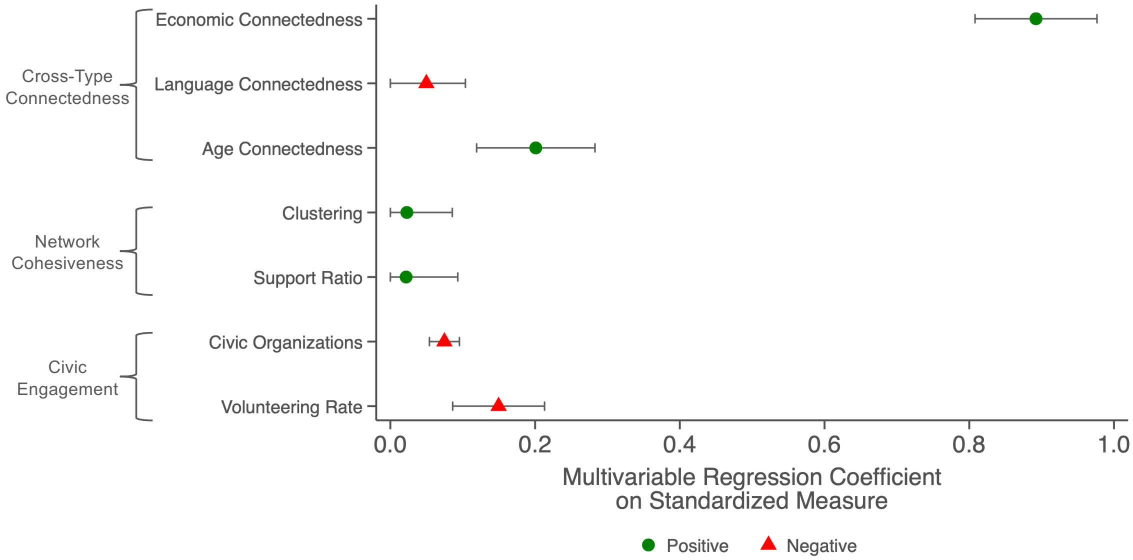

Notes: This figure replicates Figure 3 at the ZIP code level instead of the county level for the measures that are available at the ZIP code level. See note to Figure 3 for details.

SUPPLEMENTARY FIGURE 4: ZIP Code-Level Correlations between Economic Mobility and Neighborhood Characteristics

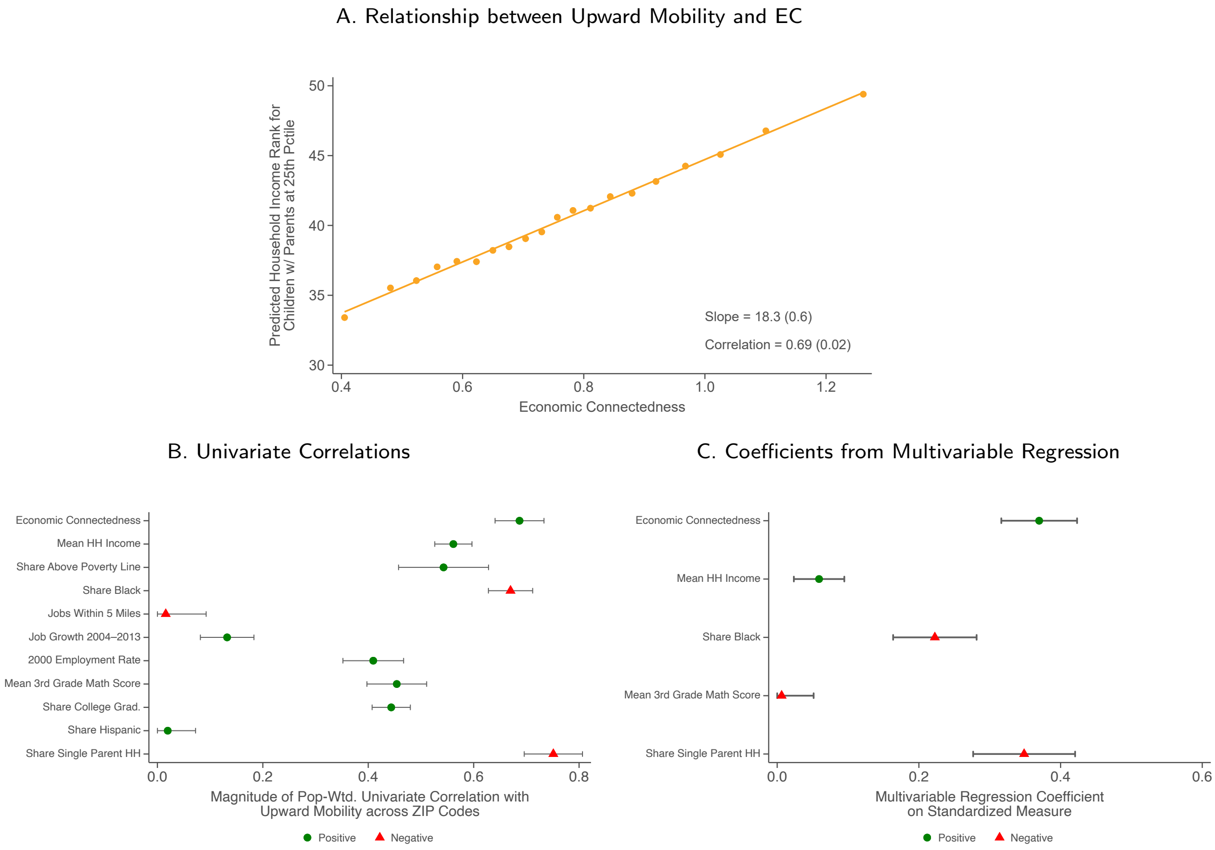

Notes: Panel A of this figure replicates the orange (below-median) series of Extended Data Figure 6a at the ZIP code level. Panels B and C of this figure replicate Figure 5 at the ZIP code level instead of the county level. Income inequality (Gini coefficient) and segregation are omitted from Panels B and C because they are traditionally measured at broader geographies. The regressions are weighted by the number of children in each ZIP code whose parents have below-national-median income. Standard errors are clustered at the county level. See notes to Figure 5 and Extended Data Figure 6 for further details.

SUPPLEMENTARY FIGURE 5: Social Capital and Upward Mobility in Counties with Predominantly White Residents

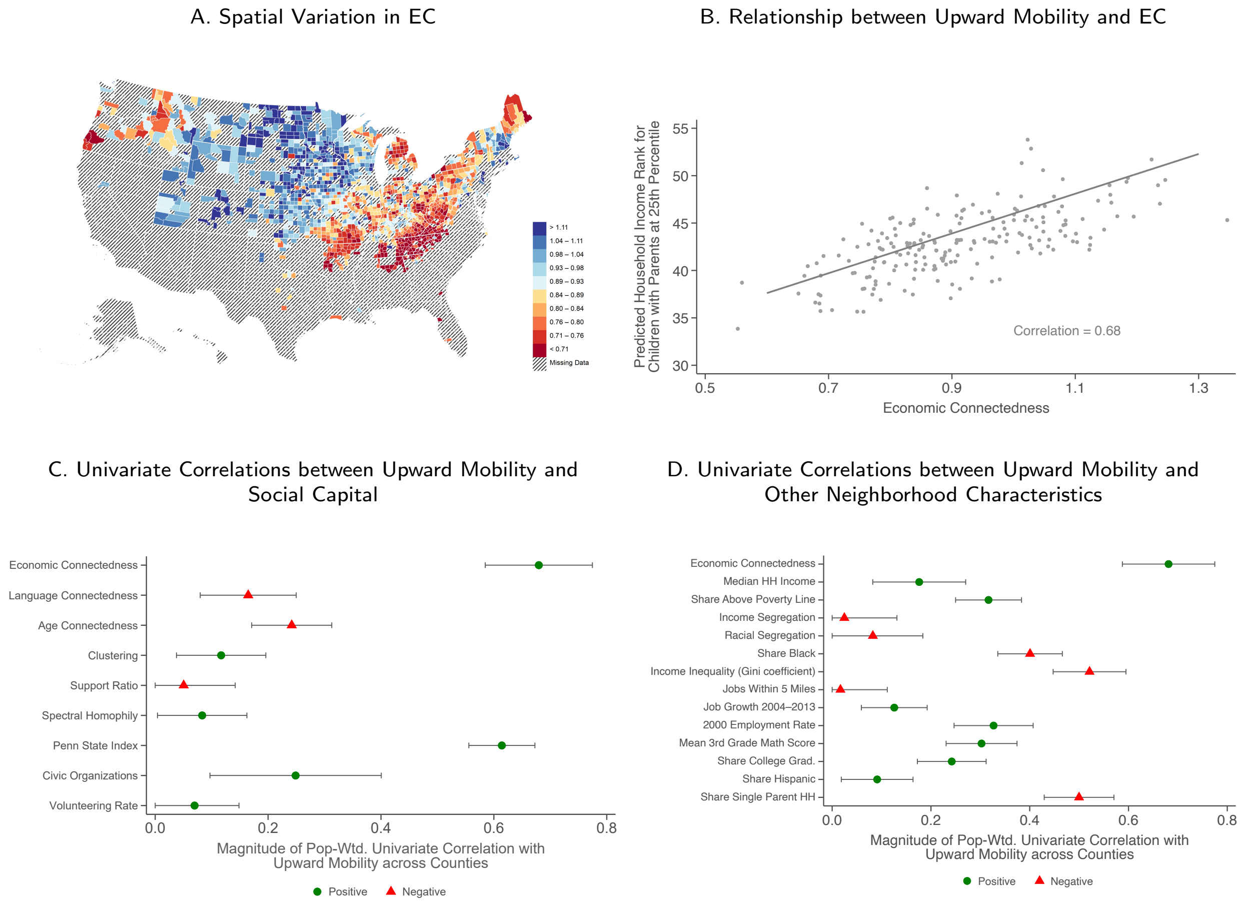

Notes: In this figure, we restrict the sample to the 1,427 counties where more than 90% of the population is white according to the 2000 Census. Panels A–D replicate Figures 2a, 4, 3a, and 5a, respectively, on this subsample of counties. See notes to those figures for further details.

SUPPLEMENTARY FIGURE 6: Regression of Counties' Causal Effects on Upward Mobility on Social Capital

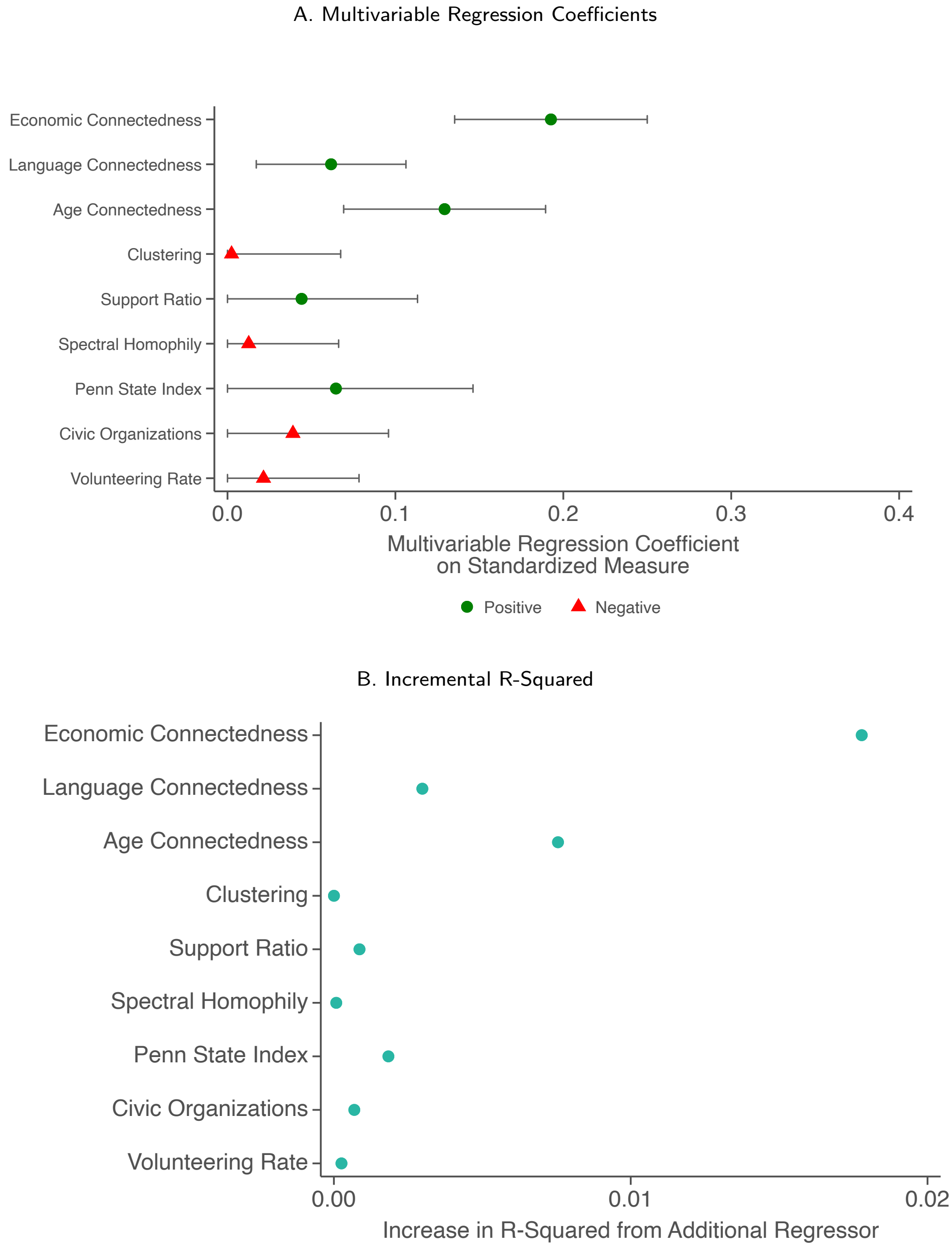

Notes: Panels A and B of this figure replicate Figure 3b and Supplementary Figure 2c, respectively, using causal instead of observational estimates of upward mobility. Causal effects on upward mobility are the raw (unshrunk) annual exposure effect estimates constructed by Chetty and Hendren (2018) by analyzing cross-county movers. Regressions are weighted by the number of children with parents who earn below the national median as calculated in the Opportunity Atlas (Chetty et al. 2018) using Census data.

SUPPLEMENTARY FIGURE 7: Upward Mobility, Economic Connectedness, and Income Levels across Counties

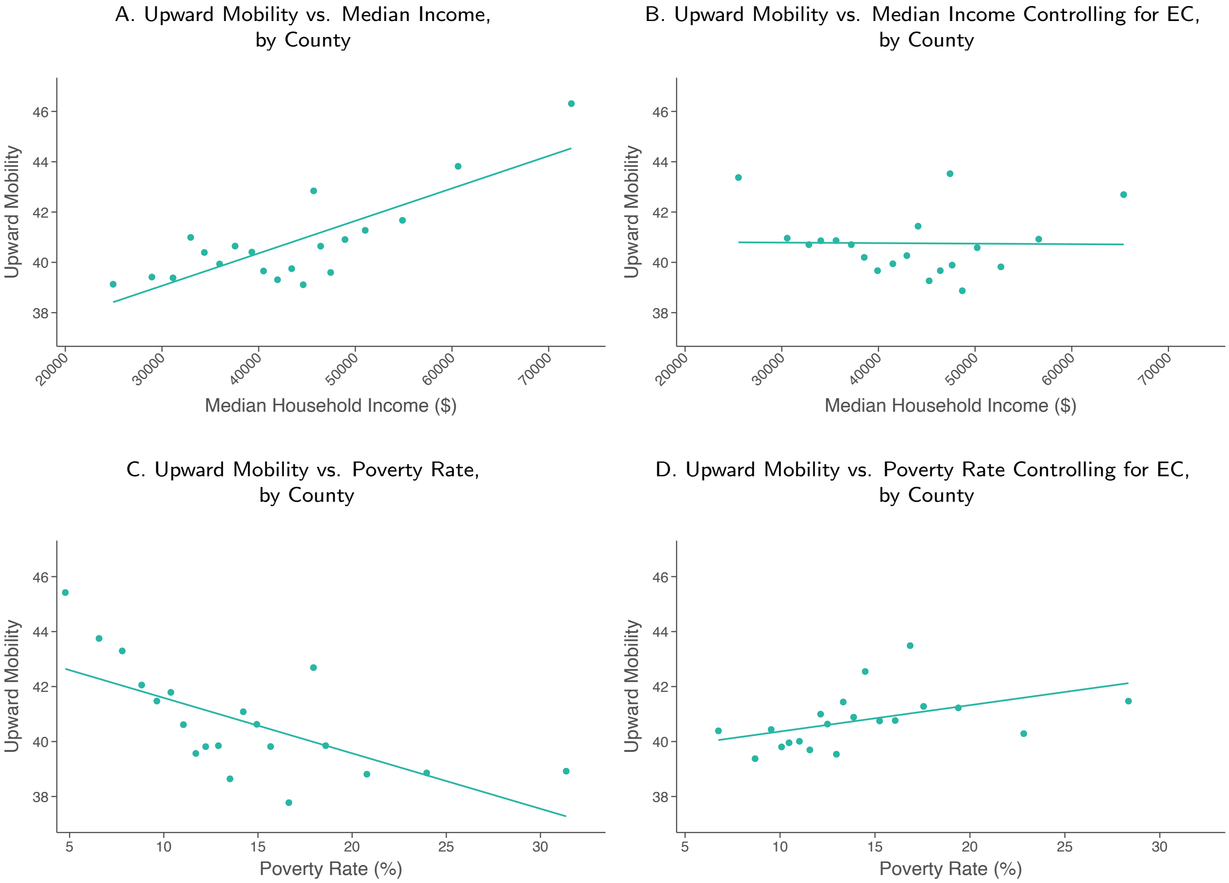

Notes: This figure shows county-level binned scatter plots of upward mobility vs. median income and poverty rates, providing non-parametric explorations of the specifications in Table 2a. The left-hand panels show raw binned scatter plots, while the right-hand panels show the same binned scatter plots controlling for economic connectedness (EC). To construct the raw binned scatter plots, we group counties into twenty bins (weighted by the number of children in each county whose parents have below-national-median income) based on values plotted on the horizontal axis (median incomes in Panels A and B, and poverty rates in Panels C and D). We then plot the (weighted) mean level of upward mobility and the income or poverty measure within each bin. The plots that control for EC are constructed by first residualizing upward mobility and the relevant horizontal-axis variable on economic connectedness using univariate OLS regressions, and then plotting a standard binned scatter plot of the residuals after adding back the means of each variable for scaling purposes. See notes to Table 2 for variable definitions and other details.

SUPPLEMENTARY FIGURE 8: Upward Mobility, Economic Connectedness, and Income Levels across ZIP Codes

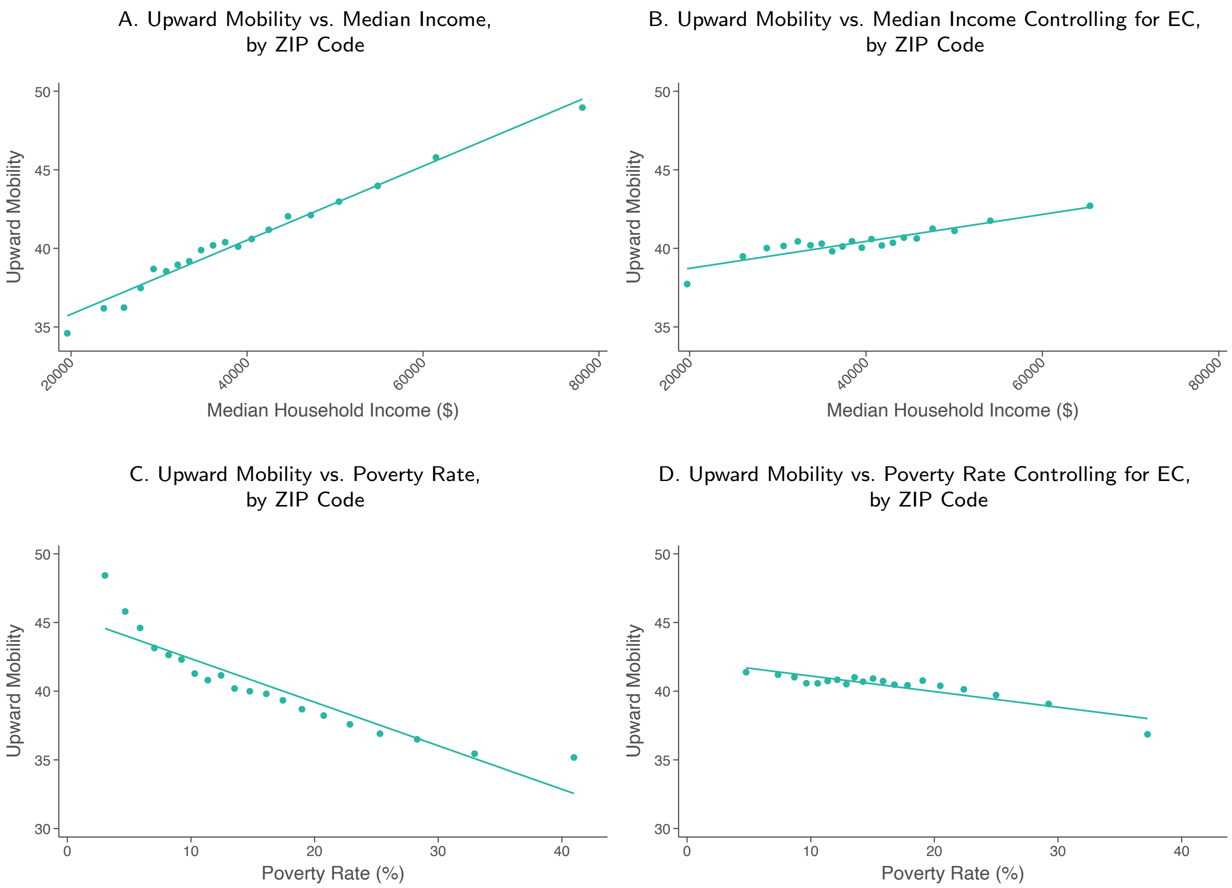

Notes: This figure presents ZIP code-level binned scatter plots of upward mobility vs. median income and poverty rates, corresponding to the linear regression specifications in Table 2a. The left-hand panels show raw binned scatter plots, while the right-hand panels show the same binned scatter plots controlling for economic connectedness (EC). See Supplementary Figure 7 for details on construction of these binned scatter plots, and the notes to Table 2 for variable definitions and other details.

SUPPLEMENTARY FIGURE 9: Upward Mobility, Economic Connectedness, and Measures of Inequality and Segregation across Counties

A. Upward Mobility vs. Income Segregation, by County

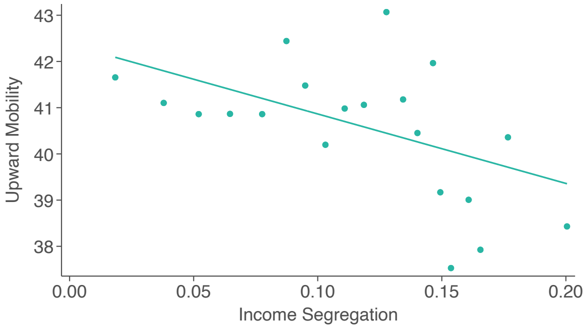

B. Upward Mobility vs. Income Segregation Controlling for EC, by County

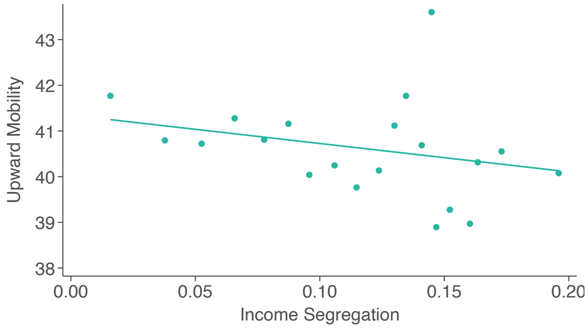

C. Upward Mobility vs. Racial Segregation, by County

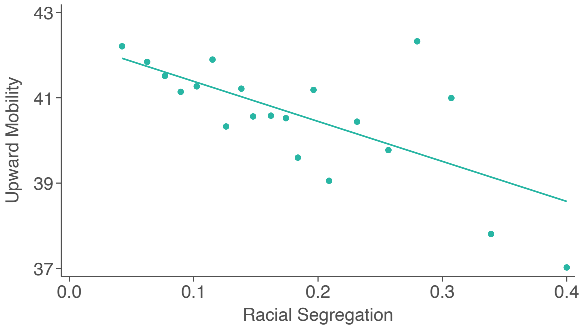

D. Upward Mobility vs. Racial Segregation Controlling for EC, by County

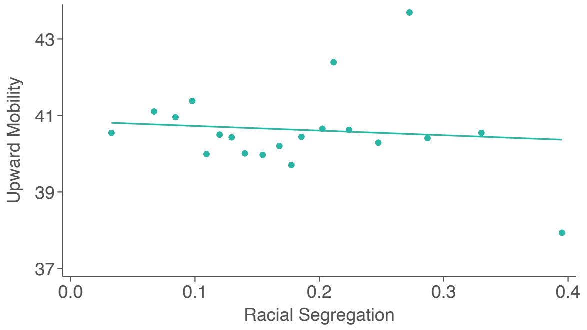

E. Upward Mobility vs. Gini Coefficient, by County

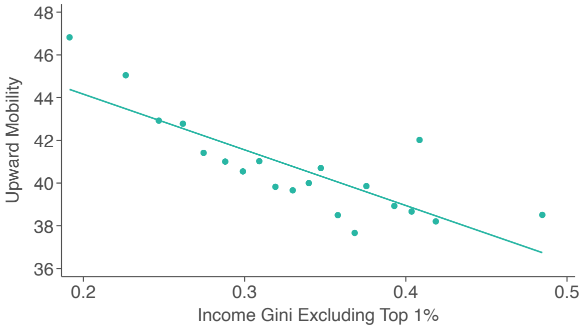

F. Upward Mobility vs. Gini Coefficient Controlling for EC, by County

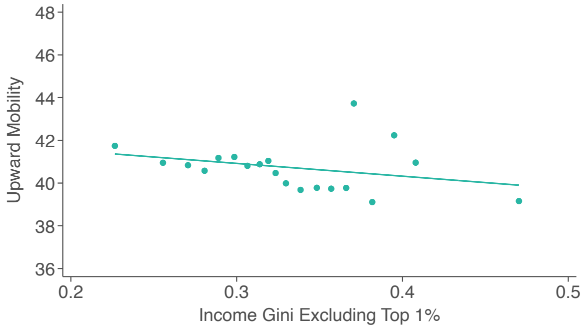

Notes: This figure presents county-level binned scatter plots of upward mobility vs. several measures of within-county inequality and segregation, corresponding to the linear regression specifications in Table 2b. The left-hand panels show raw binned scatter plots, while the right-hand panels show the same binned scatter plots controlling for economic connectedness (EC). See Supplementary Figure 7 for details on construction of these binned scatter plots, and the notes to Table 2 for variable definitions and other details.

SUPPLEMENTARY FIGURE 10: Race-Specific Upward Mobility, Economic Connectedness, and Share of Black Residents across ZIP Codes

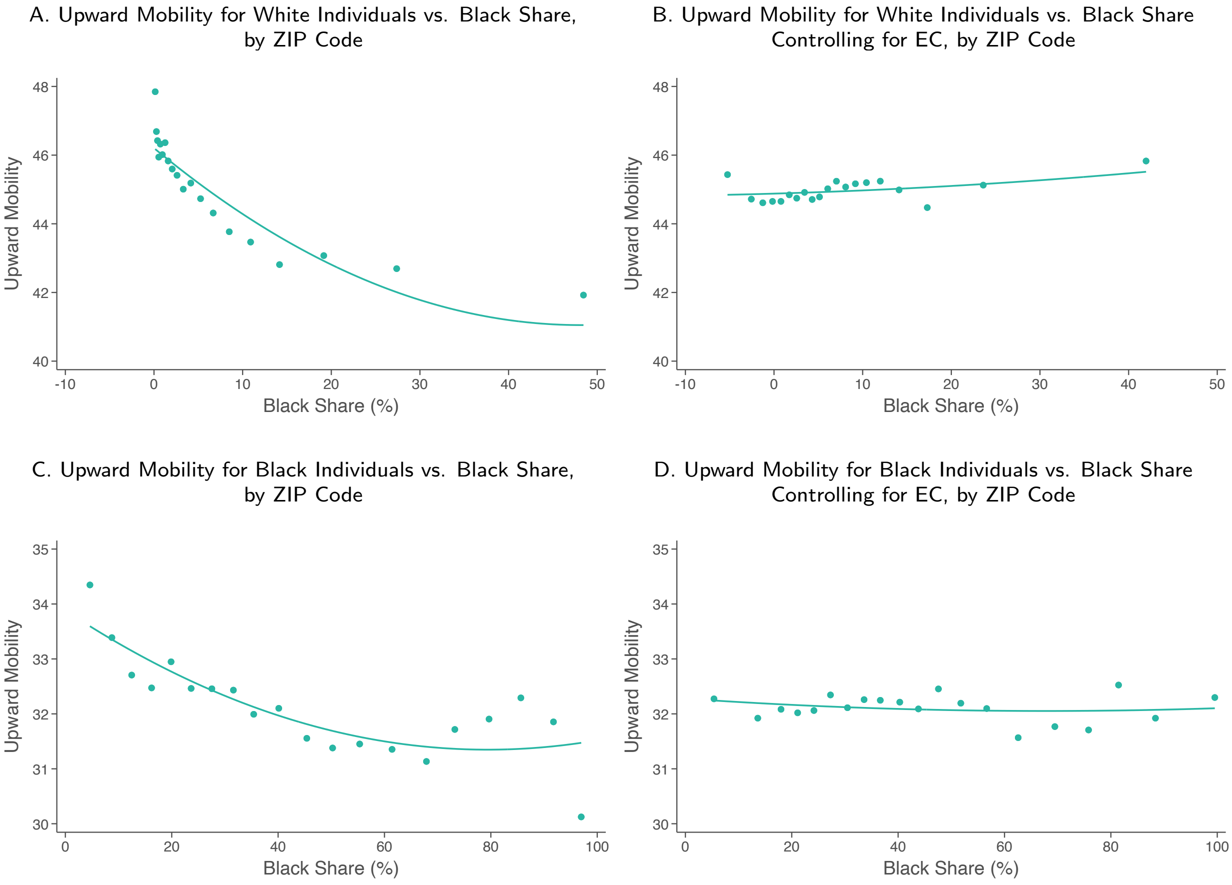

Notes: This figure presents ZIP code-level binned scatter plots of race-specific upward mobility vs. the share of Black individuals in the ZIP code, corresponding to the linear regression specifications in Table 2c. The left-hand panels show raw binned scatter plots, while the right-hand panels show the same binned scatter plots controlling for economic connectedness (EC). See Supplementary Figure 7 for details on construction of these binned scatter plots, and the notes to Table 2 for variable definitions and other details.

SUPPLEMENTARY FIGURE 11: Correlations between Social Capital and Life Expectancy

A. Correlations of Social Capital with Life Expectancy at Age 40 for Bottom-Income-Quartile Men Across Counties

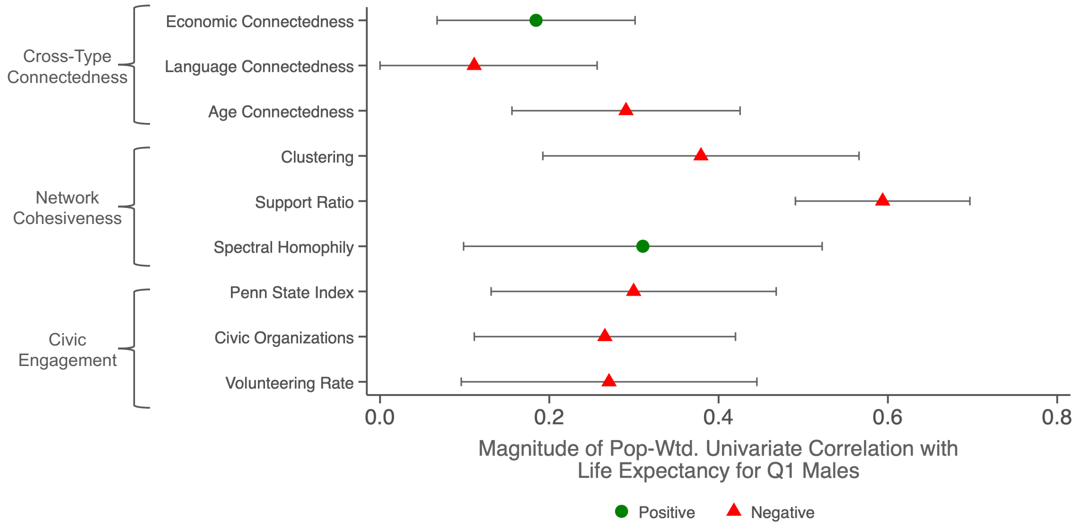

B. Life Expectancy for Bottom-Income-Quartile Men vs. Clustering Coefficient, by County

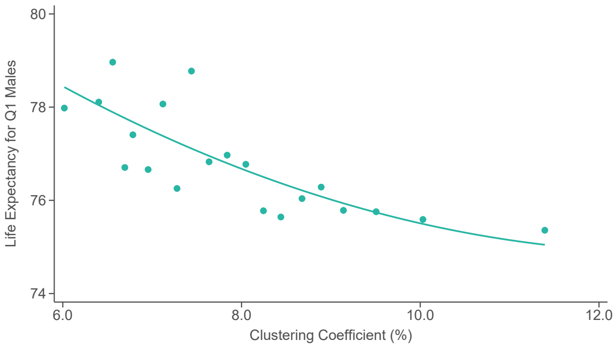

C. Life Expectancy for Bottom-Income-Quartile Men vs. Support Ratio, by County

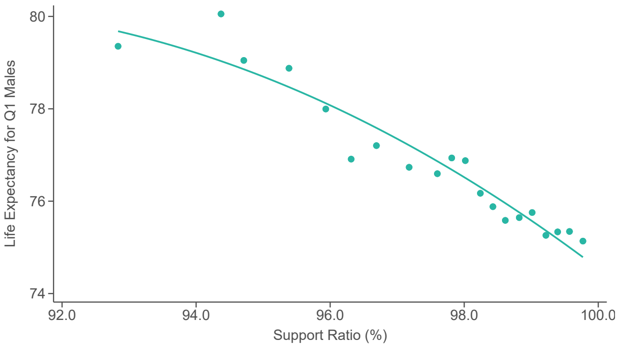

Notes: This figure shows correlations between our social capital measures and mean life expectancy at age 40 for men with income in the bottom income quartile across counties. Data on life expectancy is obtained from publicly available data released by Chetty et al. (2016), who construct these estimates using information from tax records linked to death certificate data for the U.S. population. Panel A replicates Figure 3a, using life expectancy for low-income men as the outcome instead of upward mobility, and weighting by the number of bottom-income-quartile men in each county. Panels B and C display binned scatter plots of life expectancy for low-income men vs. mean clustering coefficients and support ratios by county, respectively, again weighting by the number of bottom-income-quartile men in each county. See notes to Figure 3 and Figure 6 for details on the construction of these figures.

SUPPLEMENTARY FIGURE 12: Relative Geographic Coverage of Facebook Data

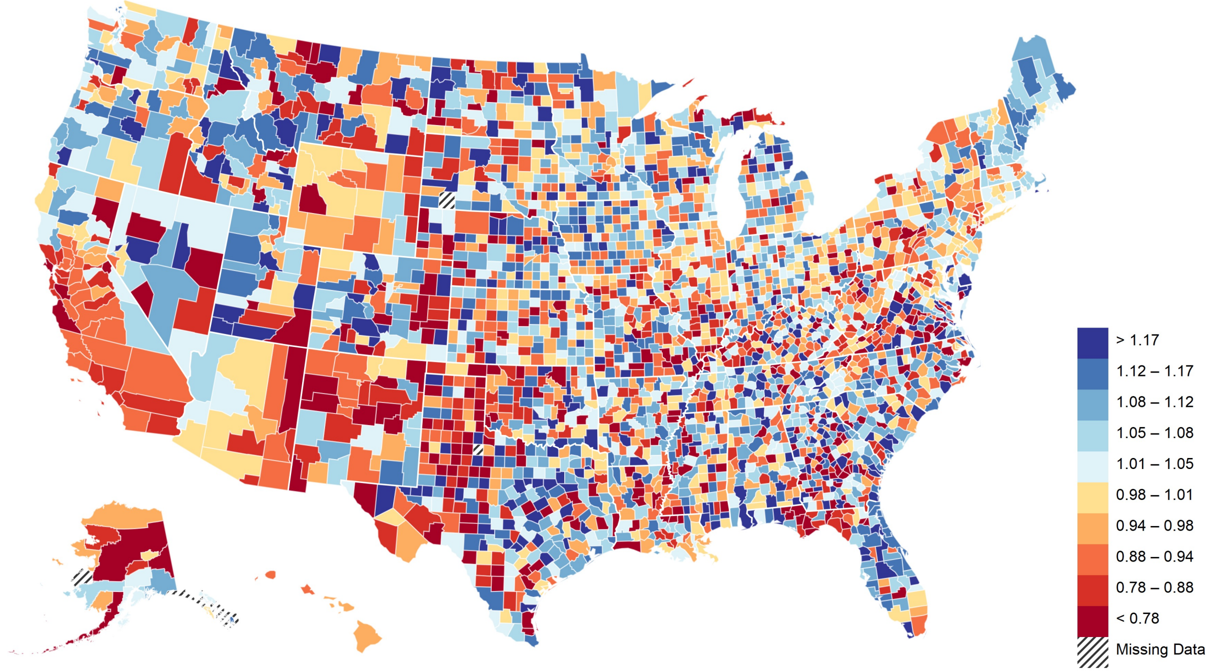

Notes: This figure shows the relative geographic variation in Facebook coverage across counties for our primary analysis sample. For each county, we compute the share of the Facebook users in our data relative to the number of 25–44 year-olds in the 2014–2018 American Community Survey (ACS). We normalize these county-level coverage estimates by the corresponding U.S.-wide coverage in our sample to obtain relative coverage estimates. For example, a value of 1.2 for a given county implies that our sample coverage rate is 20% higher in that county relative to the overall national sample coverage rate.

SUPPLEMENTARY FIGURE 13: Distribution of ZIP Code-Level Incomes in Facebook Data vs. ACS

A. Age 25 to 44

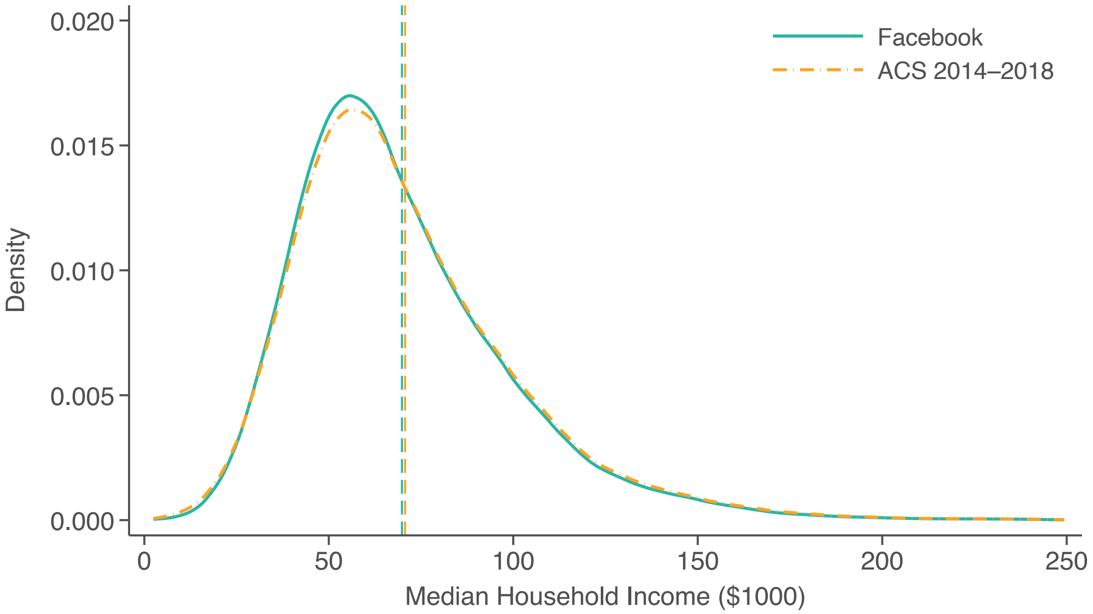

B. Age 45 to 64

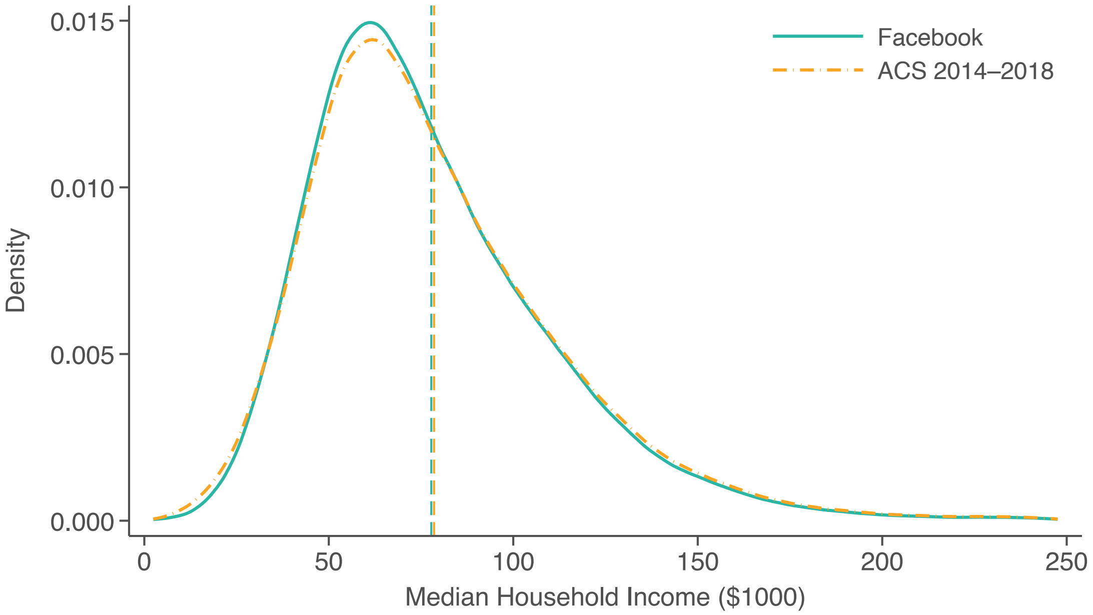

Notes: This figure evaluates the geographic representativeness of the Facebook sample in terms of median incomes by ZIP code for the 25 to 44 (Panel A) and 45 to 64 (Panel B) age groups. The dashed orange curve shows the distribution of median household income by ZIP code (as measured in the 2014–2018 American Community Survey (ACS)), weighting by the number of individuals in the relevant age group residing in each ZIP code according to the ACS. The solid green curve replicates the same density plot of ACS median household income by ZIP code, weighting instead by the number of individuals residing in each ZIP code in the relevant age group in the Facebook data. The dashed vertical lines show the median incomes in each sample (green for Facebook, orange for ACS).

SUPPLEMENTARY FIGURE 14: County-Level Correlations for Top 25% of Counties by Facebook Coverage Rates

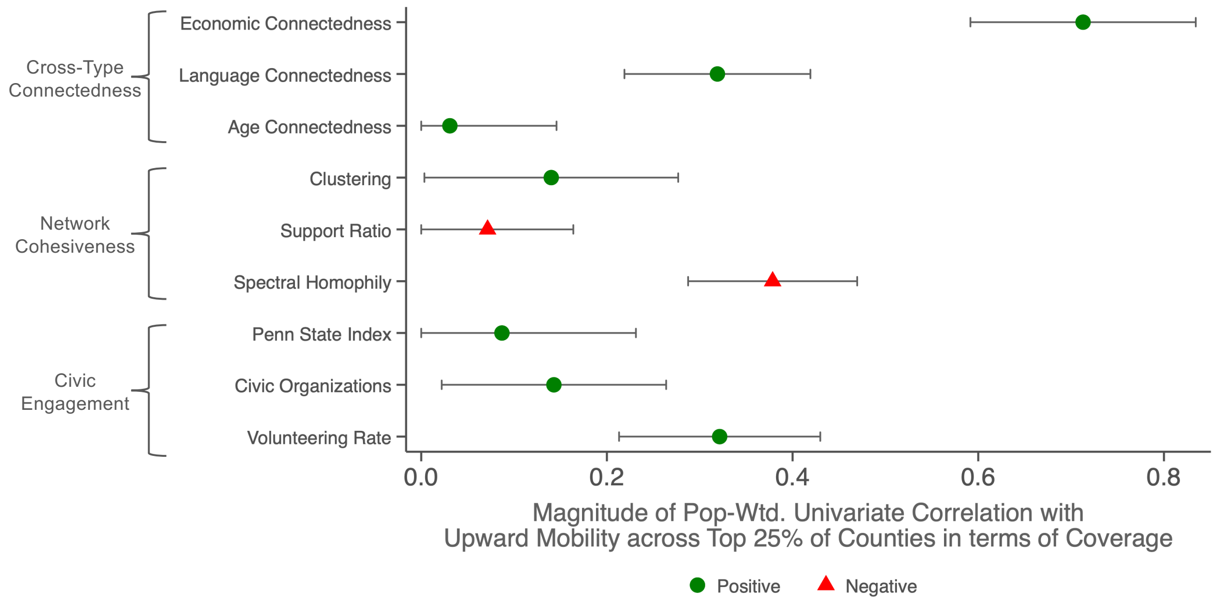

B. Upward Mobility vs. Neighbourhood Characteristics

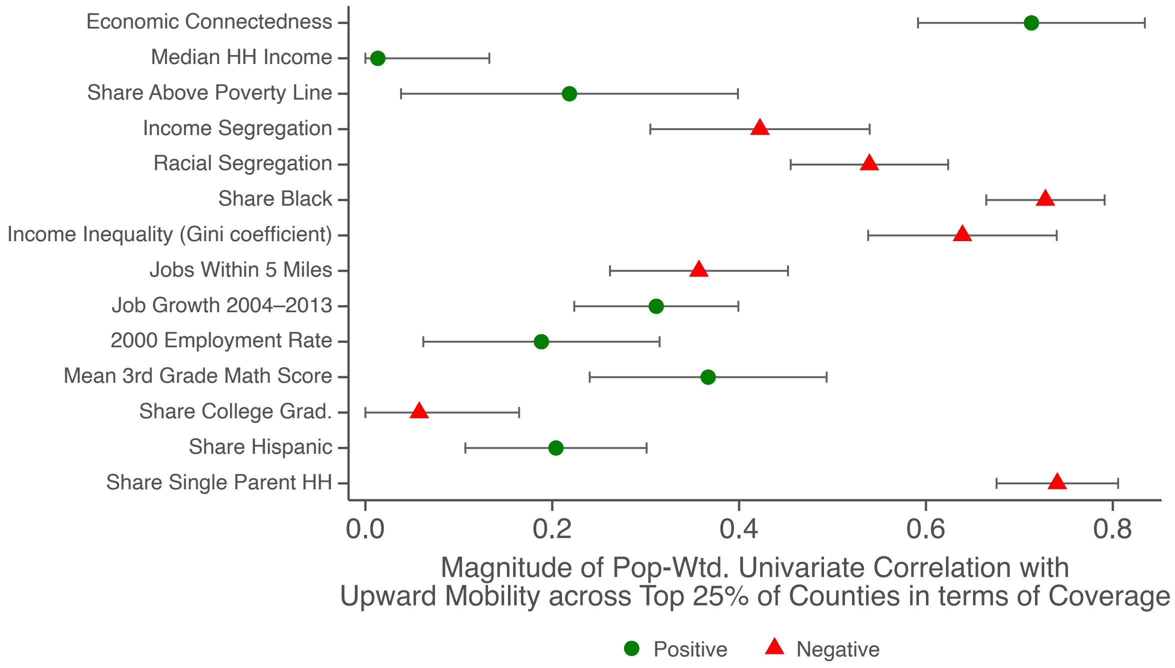

Notes: Panel A replicates Panel A of Figure 3, restricting the sample to the top 25% of counties (unweighted) in terms of Facebook coverage. Coverage is defined as the number of individuals in our primary analysis sample divided by the number of 25–44 year-olds in the 2014–2018 American Community Survey (ACS) in each county. Panel B replicates Panel A of Figure 5, again restricting the sample to the top 25% of counties in terms of coverage.

SUPPLEMENTARY FIGURE 15: Relationship Between Friends' and Own Socioeconomic Status on Instagram

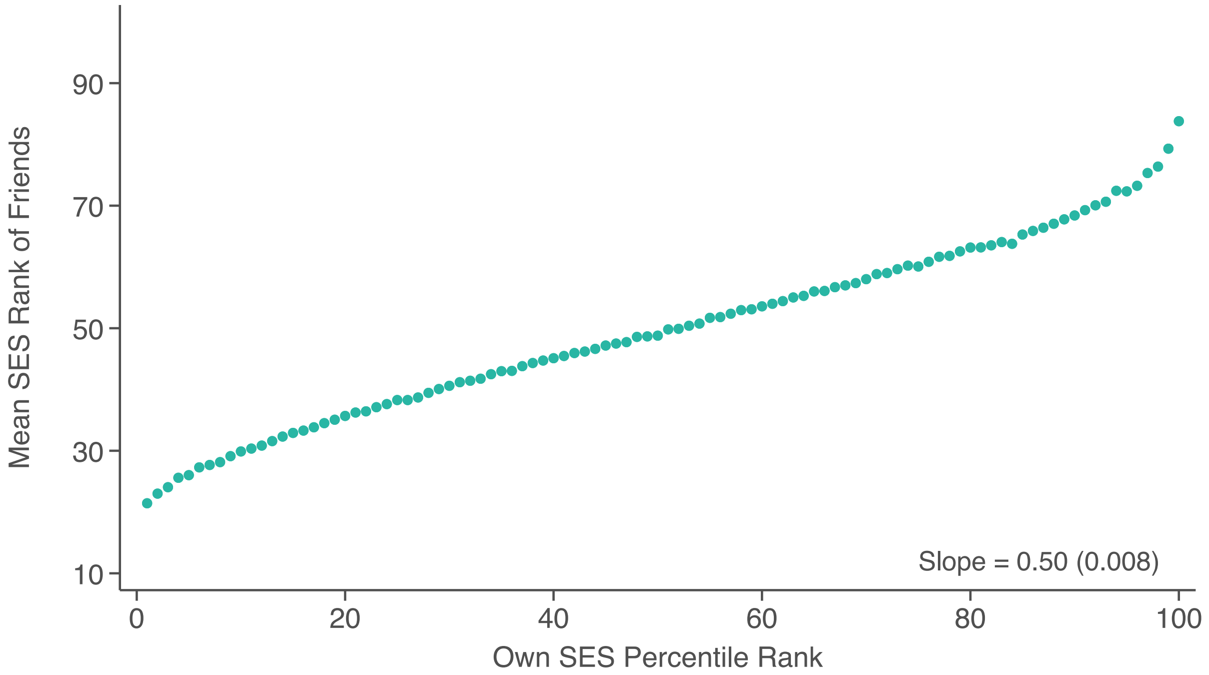

Notes: This figure replicates the series in circles in Figure 1a, plotting the mean SES rank of individuals' friends vs. individuals' own SES ranks in the Instagram sample. See Supplementary Information A.4 for details on the construction of the Instagram sample and definition of SES ranks in that sample.

SUPPLEMENTARY FIGURE 16: Proportion of Friendships by SES Percentile Rank

A. Friending Shares by Own and Friends' SES Ranks

B. Proportion of Friends by SES Percentile Rank for Individuals in the Upper Tail

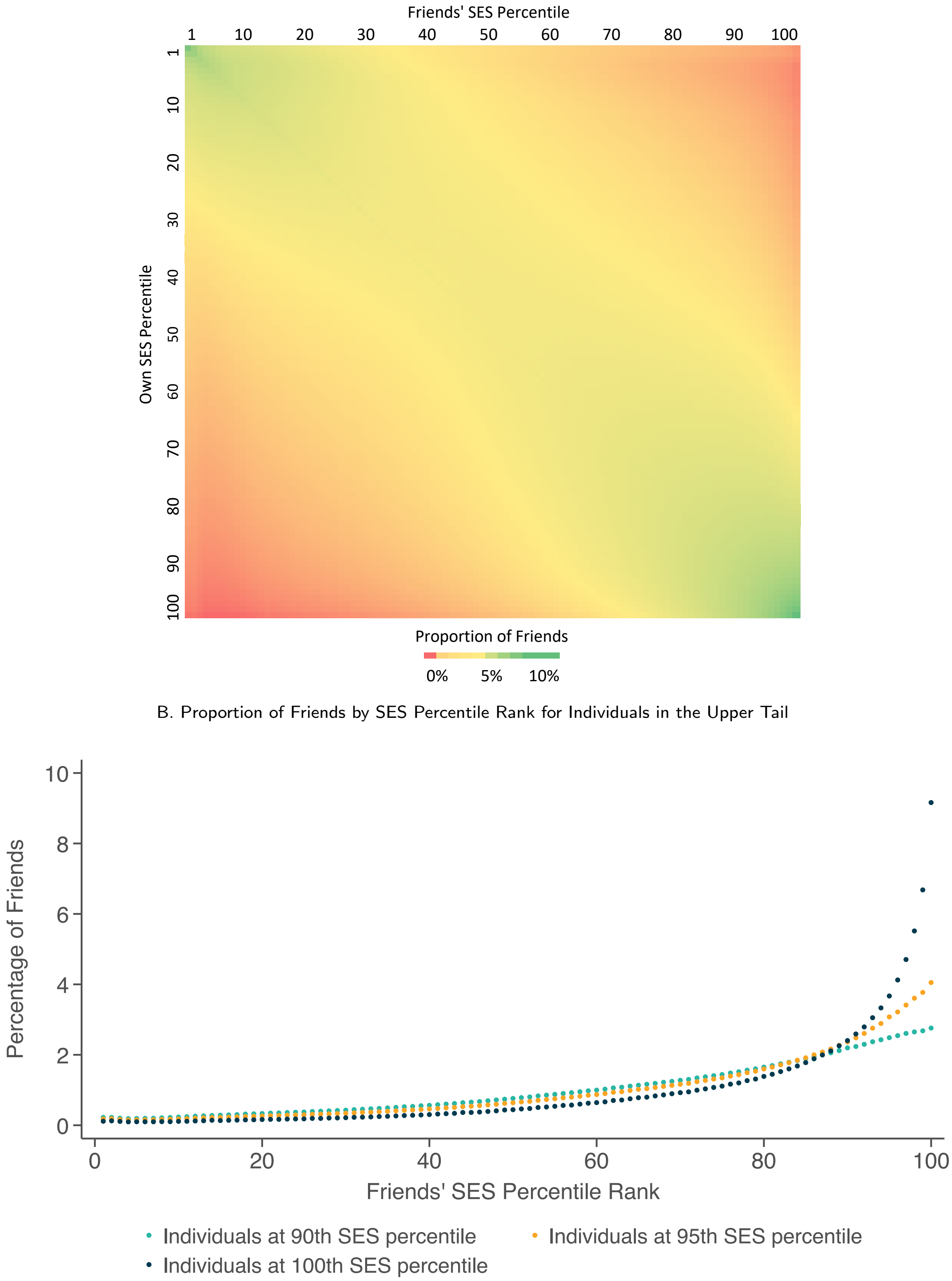

Notes: Panel A presents a heat map showing the proportion of friends in each SES percentile rank by an individual's own SES percentile rank. Red squares represent lower proportions of friends in a given SES percentile, whereas green squares represent higher proportions of friends in that percentile. Panel A replicates Extended Data Table 1a at the percentile level instead of the decile level. Panel B shows the proportion of friends by friends' SES percentile rank for individuals at the 90th, 95th, and 100th percentiles of the national SES distribution.
